# Symmetric projection optimizer: concise and efficient solving engineering problems using the fundamental wave of the Fourier series

**DOI:** 10.1038/s41598-024-56521-4

**Published:** 2024-03-12

**Authors:** Haoxiang Su, Zhenghong Dong, Yi Liu, Yao Mu, Sen Li, Lurui Xia

**Affiliations:** Spece Engineering University, Bayi Road, Huairou, Beijing, 101400 China

**Keywords:** Symmetric projection optimizer, Search mechanism, Fundamental wave, Projection plane, Engineering optimization, Applied mathematics, Computational science, Computer science, Information technology

## Abstract

The fitness function value is a kind of important information in the search process, which can be more targeted according to the guidance of the fitness function value. Most existing meta-heuristic algorithms only use the fitness function value as an indicator to compare the current variables as good or bad but do not use the fitness function value in the search process. To address this problem, the mathematical idea of the fitting is introduced into the meta-heuristic algorithm, and a symmetric projection optimizer (SPO) is proposed to solve numerical optimization and engineering problems more efficiently. The SPO algorithm mainly utilizes a new search mechanism, the symmetric projection search (SP) method. The SP method quickly completes the fitting of the projection plane, which is located through the symmetry of the two points and finds the minima in the projection plane according to the fitting result. Fitting by using the fitness function values allows the SP to find regions where extreme values may exist more quickly. Based on the SP method, exploration and exploitation strategies are constructed, respectively. The exploration strategy is used to find better regions, and the exploitation strategy is used to optimize the discovered regions continuously. The timing of the use of the two strategies is designed so that the SPO algorithm can converge faster while avoiding falling into local optima. The effectiveness of the SPO algorithm is extensively evaluated using seven test suites, including CEC2017, CEC2019, CEC2020, and CEC2022. It is also compared with two sets of 19 recent competitive algorithms. Statistical analyses are performed using five metrics such as the Wilcoxon test, the Friedman test, and variance. Finally, the practicality of the SPO algorithm is verified by four typical engineering problems and a real spacecraft trajectory optimization problem. The results show that the SPO algorithm can find superior results in 94.6% of the comparison tests and is a promising alternative for solving real-world problems.

## Introduction

Optimization is finding a suitable set of variable values to minimize (maximize) the value of some optimization objective under certain constraints. Optimization algorithms are widely used in engineering design^1–3^, engineering practice^4–6^, motion control^7–9^, and task scheduling in the real world^10–12^. At this stage, algorithms for solving optimization problems contain two main categories^13^. One is the traditional optimization method based on mathematical formulas^14–16^, and the other is the metaheuristic algorithm based on stochastic processes^17–19^.

Traditional optimization methods generally have rigorous mathematical proofs and a fixed set of computational formulas. Through the procedures, it is possible to solve low-dimensional problems effectively. However, for high-dimensional issues, using traditional methods is of high computational complexity, and it is easy to fall into local optimal solutions. Compared with conventional methods, heuristic algorithms do not have strict proofs, but through the design of stochastic processes, they can effectively solve complex real-world problems, so it has been widely researched and applied in various fields.

Currently, metaheuristic algorithms can be broadly classified into the following four categories including metaheuristic algorithms based on biogenetic information (BBAs), metaheuristic algorithms based on the behavior or organization of natural organisms (NBAs), metaheuristic algorithms based on physical or chemical phenomena (PCBAs), and metaheuristic algorithms based on mathematical methods (MBAs).

Metaheuristic algorithms based on biological genetic information use the changes in the genetic information of organisms during reproduction as inspiration for algorithm construction. The most typical of these algorithms are genetic algorithms (GA)^20^ and differential evolutionary algorithms (DE)^21^. These algorithms are mainly based on the evolutionary process of organisms in the natural world and adopt the "survival of the fittest" theory to realize the optimization of the search space. Cooperative co-evolutionary algorithms (CCEA)^22^, evolutionary mating algorithms (EMA)^23^, evolutionary field optimization algorithms (EFO)^24^, and quantum-based avian navigation optimizer algorithm (QANA)^25^ belong to this type of algorithm. The main search mechanisms of such algorithms are crossover and mutation. The crossover is the recombination of elements of different variables. The mutation is the random resetting of an element of a variable to another value. Benefiting from these powerful search mechanisms, as one of the originators of metaheuristic algorithms, the GA algorithm is still widely used in various fields due to its strong scalability and fast convergence^26–28^.

Metaheuristic algorithms based on the behavior or organization of natural organisms are the largest class of algorithms. The main idea is to use the behaviors of various types of organisms as the inspiration for building algorithms, such as animal predation behavior, plant reproduction process, and human social organization. Depending on the type of organisms, they can be further classified into categories such as animal-based metaheuristics, plant-based metaheuristics, and human behavior-based metaheuristics. Among them, animal-based metaheuristic algorithms were the first to be developed. For example, the particle swarm algorithm (PSO)^29^ performs optimization by simulating birds' feeding behavior, and the ant colony algorithm (ACO)^30^ conducts optimization by mimicking the foraging behavior of ants. It has been heavily studied in recent years. Starling murmuration optimizer (SMO)^31^, evolutionary crow search algorithm (ECSA)^32^, moth-flame optimization (MFO)^33,34^, and whale optimization algorithm (WOA)^35^ are very competitive algorithms in this category. Algorithms such as the dandelion optimizer (DO)^36^, the forest optimization algorithm (FOA)^37^, and the invasive weed optimization algorithm (IWO)^38^ are representatives of plant-based algorithms. As the preeminent representatives of intelligent creatures, humans and their group behavior have been heavily studied and applied to human-based algorithms. The human urbanization search algorithm (HUS)^39^, the human evolutionary optimization algorithm (HEOA)^40^, the human behavioral optimization algorithm (HBBO)^41^, the focus group algorithm (FG)^42^, the human learning optimization algorithm (HLO)^43^, and the brainstorming optimization algorithm (BSO)^44^ belong to this category. The main search mechanism of the NBAs is the linear combination, which is the formation of new variables by the linear combination of multiple variables, with different combinations and specific coefficients depending on the algorithm.

Metaheuristic algorithms based on physical or chemical phenomena use the laws of physics or chemical phenomena as the main inspiration for constructing the algorithm. One of the most representative algorithms is the simulated annealing algorithm(SA)^45^, which performs the search by simulating the property changes during the annealing of metals. Other competitive algorithms include the simultaneous heat transfer search (SHTS)^46^, the special relativity search algorithm (SRS)^47^, Young's double-slit experiment optimization (YDSE)^48^, the Fick's law algorithm (FLA)^49^, and the Franklin's law algorithm (FLIA)^50^. The main search mechanisms of these algorithms are weight-based combination and domain search. The weight-based combination computes the corresponding weights based on the current variables' function values and recombines the variables based on the weights. And the calculation of the weights is usually related to the laws of physics. Domain search is a random search in a small area around the current variable.

Metaheuristics based on mathematical methods are a relatively new class of metaheuristics. The main feature of this class of algorithms is the use of specific mathematical methods as inspiration for building the algorithm. Some of these algorithms are more competitive, but their search mechanisms are still essentially linear combinations or weight-based combinations, such as gradient-based optimizer (GBO)^51^, generalized normal distribution optimization (GNDO)^52^, geometric mean optimizer (GMO)^53^, arithmetic optimization algorithm (AOA)^54^, subtractive averaging base optimizer (SABO)^55^. And some algorithms contribute more unique search mechanisms. The quadratic interpolation optimization algorithm (QIO)^56^, for example, proposes to use interpolation to generate new variables, and the Triangulation topology aggregation optimizer algorithm (TTOA)^57^ creates new variables by rotation.

The specific algorithm classifications and the main search mechanisms are summarized in Table 1.

**Table 1:** Comparison of the main search mechanisms.

Category	Main search mechanisms	Global search capability	Localized search capability	Robustness	Convergence speed	Information used	Computational complexity
MBAS	Mutation	Strong	Weak	Weak	Low	Low	Low
	Crossover	General	General	Weak	General	Low	Low
NBAs	Linear combination	General	Strong	General	General	General	General
PBAs	Weight-based combination	General	Strong	General	General	Strong	High
	Neighborhood search	Weak	Extremely strong	Weak	High	General	Low
MBAs	Interpolation	Strong	Strong	Strong	General	High	High
	Rotation	Strong	General	General	General	Low	High

Table 1 compares the various types of search mechanisms regarding global search capability, local search capability, robustness, convergence speed, use of known information, and computational complexity methods. As seen from the table, each search mechanism has advantages and limitations. Therefore, when designing meta-heuristic algorithms, the ability of the algorithm is improved by using multiple search mechanisms together. One of the more specific indicators is the use of known information, mainly the use of the values of the fitness function. In most search mechanisms, this is used only as an indicator of the merit of the variables and is not involved in generating new variables. Weight-based combination and interpolation are two search mechanisms that use known information entirely. The results show that the full use of known information helps enhance the search capability and convergence speed, but the computational complexity of both search mechanisms is high.

Therefore, this paper profoundly researches the search mechanism and proposes one that can fully use the known information with low computational complexity. Fourier series are introduced into metaheuristic algorithms, the properties of the Fourier series are analysed, and a search mechanism using three symmetry points to realize the optimal position search within a specific projection plane is proposed. Based on the search mechanism, a symmetric projection optimizer (SPO) is constructed with strong search capability, fast convergence speed, and high robustness. The proposed search mechanism does not rely on a complex search process and can realize global and local search modes through the same computational formulas, making the SPO algorithm's search process concise and efficient.

Two sets of experiments are designed to validate the SPO algorithms and the search mechanisms for them. One set of experiments selects eight powerful mathematics-based algorithms for comparative validation; the other set selects nine powerful algorithms from other classes. These two sets of algorithms are compared and experimented with in seven test suites, including CEC2017 with 30-dimensional, CEC2017 with 50-dimensional, CEC2017 with 100-dimensional^58^, CEC2019^59^, CEC2020^60^, CEC2022 with 10-dimensional, and CEC2022 with 20-dimensional^61^. The effectiveness of the SPO algorithm is also verified on four engineering problems and a spacecraft trajectory optimization problem. The results show that the SPO algorithm finds results closer to the optimum than the other algorithms under the same conditions.

The main contributions of this paper can be summarized as follows:

1. Introducing fitting into the search process enhances the purposefulness and efficiency of the search. Using the fitness function value as the output and the distance within the projected plane as the input, a fast fitting of the projected plane using the Fourier function is realized by two symmetry points, which enables the meta-heuristic algorithm to find the extreme points that can exist within the projected plane based on the fitting results.
2. A new optimizer called symmetric projection optimizer is constructed. Two search strategies based on the SP search mechanism are presented: the exploration and exploitation strategies. The SPO algorithm's overall performance is improved by combining the two sets of strategies. In the exploration strategy, two individuals far apart are used to perform the SP search, thus realizing a global search of the entire projective surface. Two closely spaced individuals implement the local search using the SP mechanism in the exploitation strategy.
3. The effectiveness of the SPO algorithm is confirmed by seven test suites, including CEC2017, CEC2019, CEC2020, and CEC2022. The results were evaluated using the Wilcoxon test, the Friedman test, and three metrics and compared with two groups of 19 recent competitive algorithms.
4. The practicality of the SPO algorithm is verified by four classical engineering cases and a real-world spacecraft trajectory optimization problem.

The remainder of the paper is structured as follows: in “Symmetric projection optimizer” section, the Fourier series and symmetric projection search method are analysed and derived in detail, and the specific procedure of the SPO algorithm is given. “Performance tests” section explains two sets of comparison algorithms and test parameters, and the experimental results of the two comparison algorithms in the seven test suites are presented and analysed. “Engineering problems tests” section validates the SPO algorithm through four practical engineering problems. A real spacecraft trajectory optimization problem is solved in “Spacecraft trajectory optimization using SPO” section and compared with 11 more recent competitive algorithms. Finally, in “Conclusion and outlook” section, the research is summarized, the specific advantages of the proposed algorithm are analysed, and future research directions are given.

## Symmetric projection optimizer

### The Fourier series

The French mathematician Fourier proposed that any periodic function satisfying the Dirichlet conditions could be made by superimposing a sequence of sine and cosine functions of different frequencies. These infinite series composed of sine and cosine functions are called Fourier series. Simultaneously, for nonperiodic functions with finite intervals, it is also possible to make them decomposable using Fourier series through period extensions^62^. Fourier series is widely used as an essential mathematical tool in signal processing and mathematical analysis^63^. In data analysis, Fourier series are used to fit and predict trends and cyclical variations in data to support decision-making and forecasting. The fitting equation is

1 $$ f(x) = \frac{{a_{0} }}{2} + \sum\limits_{1}^{\infty } {\left[ {a_{n} \cos (n\omega x) + b_{n} \sin (n\omega x)} \right]} $$

**Figure 1:**
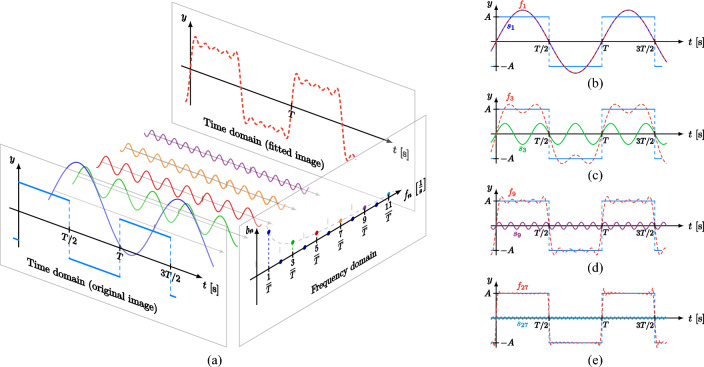
The process of fitting a curve using Fourier series. (**a**) Observed curves from different dimensions. (**b**) Fitting a curve using the fundamental wave. (**c**) Fitting a curve using 1-3th harmonic. (**d**) Fitting a curve using 1-9th harmonic. (**e**) Fitting a curve using 1-27th harmonic.

In Eq. (1), the waveform produced by the superposition of the sine and cosine functions when n = 1 is called the fundamental wave or 1st harmonic, and the waveform they have when n > 1 is called the nth harmonic. As can be seen from the formula, fitting a function using the Fourier series makes it as close as possible to the original function when superimposed by adjusting the amplitude, frequency, and phase of these fundamental and harmonics. The process of fitting is shown in Fig. 1.

From Fig. 1a, it is clear that the Fourier series decomposes the function from the frequency domain. Figure 1b–e demonstrates the fitting effect using different order harmonics, where *f*_1_-*f*_27_ represents the fit results after the superposition of varying order harmonics, and *s*_1_-*s*_27_ means the nth harmonic. As can be seen from the figure, the higher the order harmonic used, the better the fit to the curve. It is worth noting that the fundamental wave has sufficiently captured the function's broad trend, with the higher orders of the Fourier series serving only to fine-tune the fitted curve's specifics—a feature that has minimal bearing on the trend. Therefore, this paper proposes to estimate the curve's trend only using the fundamental wave of the Fourier series. Then, the formula is calculated as

2 $$ f(x) = p_{0} + p_{1} \sin (\omega x) + p_{2} \cos (\omega x) $$

Meanwhile, using the relationship between the trigonometric functions, Eq. (2) can also be rewritten as

3 $$ f(x) = p_{0} + \sqrt {p_{1}^{2} + p_{2}^{2} } \sin (\omega t + \varphi ),\tan \varphi = \frac{{p_{2} }}{{p_{1} }} $$

**Figure 2:**
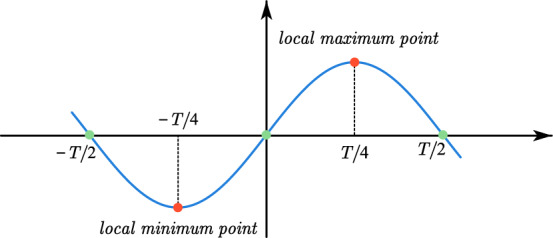
The extreme points of the sine function.

According to Eq. (3), the final fit can be rewritten as a sine function. Furthermore, its extreme points can easily be found for a sine function limited to one period. The extreme points of the sin function are shown in Fig. 2.

For Eq. (3), the extreme points are

4 $$ \left\{ {\begin{array}{*{20}c} {x_{min} = \frac{1}{\omega }\left[ {\arctan \left( {\frac{{p_{2} }}{{p_{1} }}} \right) - \frac{\pi }{2}} \right]} \\ {x_{\max } = \frac{1}{\omega }\left[ {\arctan \left( {\frac{{p_{2} }}{{p_{1} }}} \right) + \frac{\pi }{2}} \right]} \\ \end{array} ,} \right.T = 2\pi $$

More excitingly, for a given period *ω*, there are only three unknowns in Eq. (2), meaning we need only three points to estimate the overall trend of the function. Then, *p*_1_ and *p*_2_ can be obtained from the three known points by

5 $$ \left\{ {\begin{array}{*{20}c} {p_{0} + p_{1} \sin (\omega x_{0} ) + p_{2} \cos (\omega x_{0} ) = f_{0} } \\ {p_{0} + p_{1} \sin (\omega x_{1} ) + p_{2} \cos (\omega x_{1} ) = f_{1} } \\ {p_{0} + p_{1} \sin (\omega x_{2} ) + p_{2} \cos (\omega x_{2} ) = f_{2} } \\ \end{array} } \right. $$

Solving the above system of equations yields

6 $$ \left[ {\begin{array}{*{20}c} {p_{0} } \\ {p_{1} } \\ {p_{2} } \\ \end{array} } \right] = \left[ {\begin{array}{*{20}c} 1 & {\sin (\omega x_{0} )} & {\cos (\omega x_{0} )} \\ 1 & {\sin (\omega x_{1} )} & {\cos (\omega x_{1} )} \\ 1 & {\sin (\omega x_{2} )} & {\cos (\omega x_{2} )} \\ \end{array} } \right]^{ - 1} \left[ {\begin{array}{*{20}c} {f_{0} } \\ {f_{1} } \\ {f_{2} } \\ \end{array} } \right] $$

With the above formula, in the case of three points all the time, we can predict the trend of the curve and get its possible extreme points.

### Symmetric projection search method

In the previous subsection, the Fourier series and its fundamental wave were analysed, and a method for predicting the curve's trend and finding the extreme points using three points was discussed and given. However, there are two problems if one wants to use it in the metaheuristic algorithm: (1) In real optimization problems, the number of independent variables is usually tens or hundreds. If each dimension of the independent variables is dealt with separately, that will dramatically increase the computational complexity. (2) In the computational process, each prediction requires solving the inverse matrix of a third-order matrix, which is less computationally intensive each time but can take much time, considering that this process will be heavily used in the search process. Therefore, this paper proposes a concise and easy-to-compute method, namely the symmetric projection search method.

First, select any two points on the optimization function and use the direction where the first point points to the second point as the base direction, notated as

7 $$ \left\{ \begin{aligned} & X_{0}  = [x_{0}^{0} ,x_{0}^{1} ,...,x_{0}^{n} ] \hfill \\ & X_{1} = [x_{1}^{0} ,x_{1}^{1} ,...,x_{1}^{n} ] \hfill \\ & R = X_{1} - X_{0} \hfill \\ \end{aligned} \right. $$

Then, the point of symmetry of *X*_1_ about *X*_0_ is

8 $$ X_{2} = X_{0} - R $$

The distance between these two points from the first point is

9 $$ d_{10} = \sqrt {\sum\limits_{i = 1}^{n} {(x_{1} - x_{0} )^{2} } } = - d_{20} = - \sqrt {\sum\limits_{i = 1}^{n} {(x_{2} - x_{0} )^{2} } } $$

Then, regarding the first point as the origin of the coordinates, the curve is fitted with a fundamental wave of period ω to obtain

10 $$ \left\{ {\begin{array}{*{20}l} {p_{0} + p_{1} \sin (0) + p_{2} \cos (0) = f_{0} } \hfill \\ {p_{0} + p_{1} \sin (\omega d_{10} ) + p_{2} \cos (\omega d_{10} ) = f_{1} } \hfill \\ {p_{0} + p_{1} \sin (\omega d_{20} ) + p_{2} \cos (\omega d_{20} ) = f_{2} } \hfill \\ \end{array} } \right. $$

Considering the relationship between the trigonometric functions, the above equation can be written as

11 $$ \left[ {\begin{array}{*{20}c} 1 & 0 & 1 \\ 1 & a & b \\ 1 & { - a} & b \\ \end{array} } \right]\left[ {\begin{array}{*{20}c} {p_{0} } \\ {p_{1} } \\ {p_{2} } \\ \end{array} } \right] = \left[ {\begin{array}{*{20}c} {f_{0} } \\ {f_{1} } \\ {f_{2} } \\ \end{array} } \right] $$

where

12 $$ \left\{ \begin{gathered} a = \sin (\omega d_{10} ) \hfill \\ b = \cos (\omega d_{10} ) \hfill \\ \end{gathered} \right. $$

Using the Gaussian elimination method, one can obtain

13 $$ \left[ {\begin{array}{*{20}l} {p_{0} } \\ {p_{1} } \\ {p_{2} } \\ \end{array} } \right] = \left[ {\begin{array}{*{20}l} {f_{0} - p_{2} } \\ {\frac{{p_{0} + bp_{2} - f2}}{a}} \\ {\frac{{{{(f_{1} + f_{2} )} \mathord{\left/ {\vphantom {{(f_{1} + f_{2} )} 2}} \right. \kern-0pt} 2} - f0}}{b - 1}} \\ \end{array} } \right] $$

Then, the coordinate of the optimal position can be obtained by

14 $$ X_{new} = t_{min} \cdot R/d_{10} + X_{0} $$

The above case requires that the point of symmetry *x*_1_ about *x*_0_ is within the valid range. If *x*_2_ is outside the valid range, the midpoint of *x*_0_ and *x*_1_ can be taken as *x*_2_

15 $$ X_{2} = \frac{{X_{1} - X_{0} }}{2} $$

In this case, *x*_1_ and *x*_0_ are points of symmetry concerning each other and are symmetric about *x*_2_. Then, the coordinate of the optimal position can be obtained by

16 $$ X_{new} = t_{min} \cdot R/d_{10} + X_{2} $$

To further illustrate the effectiveness of the symmetric projection search method, the search results of a standard two-dimensional optimization function are shown. The function equation is shown below.

17 $$ f(x,y) = x^{2} - y^{2} ,x \in [ - 100,100],y \in [ - 100,100] $$

**Figure 3:**
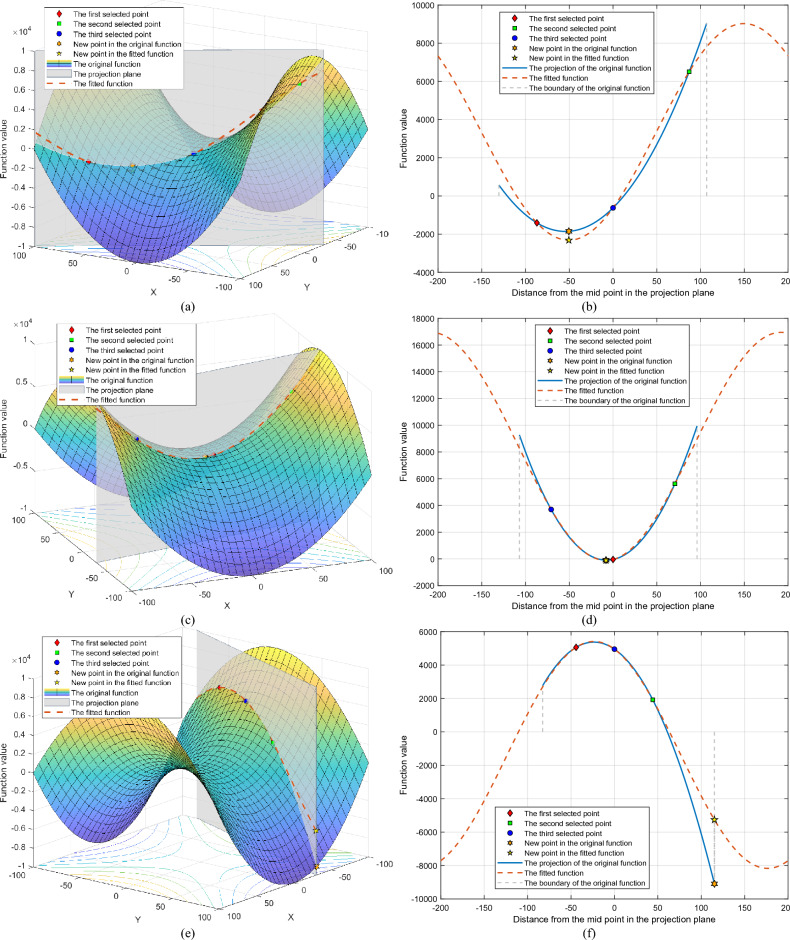

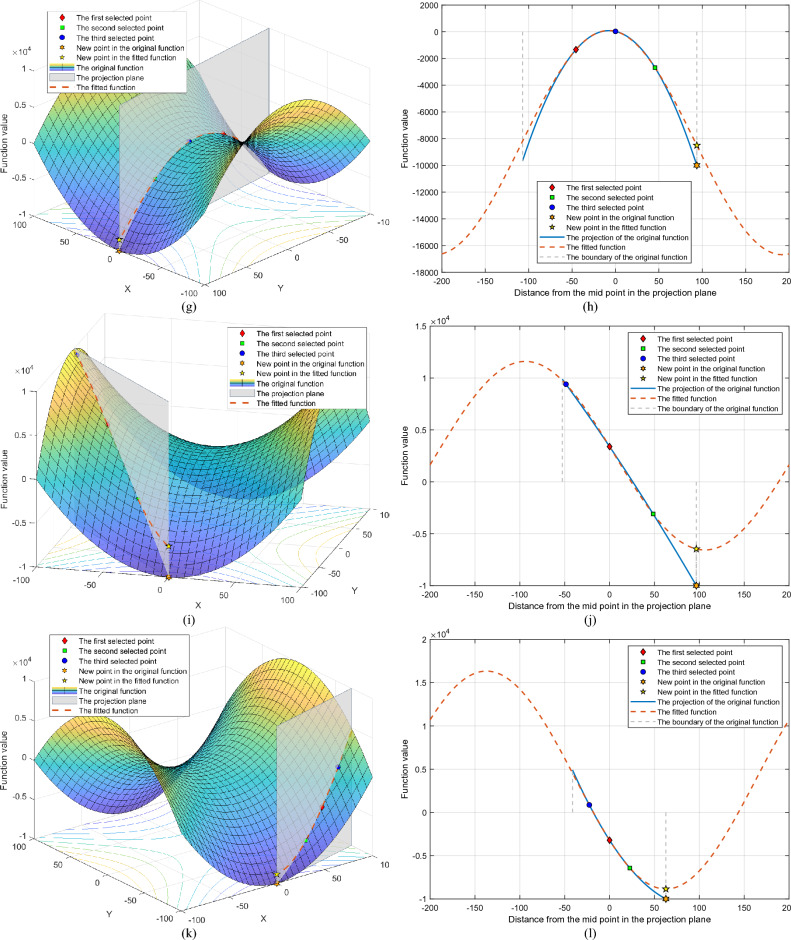
Search behaviors of the symmetric projection search method. (**a**) Case 1: search behaviors on *f*(*x*). (**b**) Case 1: search behaviors on the projection direction. (**c**) Case 2: search behaviors on *f*(*x*). (**d**) Case 2: search behaviors on the projection direction. (**e**) Case 3: search behaviors on *f*(*x*). (**f**) Case 3: search behaviors on the projection direction. (**g**) Case 4: search behaviors on *f*(*x*). (**h**) Case 4: search behaviors on the projection direction. (**i**) Case 5: search behaviors on *f*(*x*). (**j**) Case 5: search behaviors on the projection direction. (**k**) Case 6: search behaviors on *f*(*x*). (**l**) Case 6: search behaviors on the projection direction.

Some points were randomly selected, and the objective function was searched using the symmetric projection search method. The specific information on these points and the search situation is shown in Table 2. Meanwhile, the fitting and search results are shown in Fig. 3.

**Table 2:** Search result of the symmetric projection search method.

Case	Selected points	Function value	Projection direction	Period	Origin	Minimizer
1	*X*_0_ = [63.4606, 73.7389] *X*_1_ = [− 83.1128, − 20.0435] *X*_2_ = [− 9.8261, 36.8477]	*f*_0_ = − 1410.1781 *f*_1_ = 6506.0020 *f*_2_ = − 624.2488	R = [− 0.8423, − 0.5390]	0.0157	*X*_2_	*X*_3_ = [32.7137, 54.0061] *f*_3_ = − 1852.9528
2	*X*_0_ = [5.4285, − 8.5151] *X*_1_ = [75.0743, 3.6104] *X*_2_ = [− 64.2172, − 20.6407]	*f*_0_ = − 43.0382 *f*_1_ = 5623.1183 *f*_2_ = 3697.8143	R = [0.9852, 0.1715]	0.0157	*X*_0_	*X*_3_ = [− 2.4917, − 9.8941] *f*_3_ = − 91.6840
3	*X*_0_ = [− 86.4014, − 49.0420] *X*_1_ = [− 55.1920, 33.5665] *X*_2_ = [− 70.7967, − 7.7377]	*f*_0_ = 5060.0952 *f*_1_ = 1919.4432 *f*_2_ = 4952.3034	R = [0.3534, 0.9355]	0.0157	*X*_2_	*X*_3_ = [− 30.0935, 100.0000] *f*_3_ = − 9094.3831
4	*X*_0_ = [12.5754, − 38.6844] *X*_1_ = [4.3491, 52.0369] *X*_2_ = [8.4623, 6.6762]	*f*_0_ = − 1338.3414 *f*_1_ = − 2688.9223 *f*_2_ = 27.0374	R = [− 0.0903, 0.9959]	0.0157	*X*_2_	*X*_3_ = [0.0000, 100.0000] *f*_3_ = − 10,000.0000
5	*X*_0_ = [− 64.7295, − 28.2079] *X*_1_ = [− 32.1939, − 64.2934] *X*_2_ = [− 97.2651, 7.8777]	*f*_0_ = 3394.2211 *f*_1_ = − 3097.1980 *f*_2_ = 9398.4323	R = [0.6696, − 0.7427]	0.0157	*X*_0_	*X*_3_ = [0.0000, − 100.0000] *f*_3_ = − 10,000.0000
6	*X*_0_ = [60.3161, − 82.7648] *X*_1_ = [38.6075, − 88.9680] *X*_2_ = [82.0247, − 76.5617]	*f*_0_ = − 3211.9899 *f*_1_ = − 6424.7714 *f*_2_ = 866.3597	R = [− 0.9615, − 0.2748]	0.0157	*X*_0_	*X*_3_ = [− 0.0000, − 100.0000] *f*_3_ = − 10,000.0000

Cases 1 and 2 show the result of the symmetric projection search method when the projection direction is a convex function. From Fig. 3b and d, it can be seen that the symmetric projection search method can fit the function in the projection direction a lot and find its minima. From Fig. 3a,c and Table 1, it can be seen that the points found are indeed the minima of the optimization function in the current projection direction.

Cases 3 and 4 show the result of the symmetric projection search method approach under the projection direction, which is a concave function. From Fig. 3f and h, it can be seen that the fundamental wave gives a better estimation of the variation of the function and finds its minima. Figure 3e,g and Table 2 show that the points found are also the minima of the optimized function in the current projection direction.

Cases 5 and 6 show the search results of the symmetric projection search method when the projection plane is a monotone function. Figure 3i–l shows that the symmetric projection method enables an efficient search in such situations.

It is particularly noteworthy that in cases 4–6, the extreme points searched for in the projection direction are likewise the optimal points of the optimized function in the domain of definition. The above results show that when the search direction is correct, only one search is needed to find the most valuable point of the function by the projective symmetry method.

### Search strategy under the symmetric projection search method

During the search process, many algorithms categorize search processes into multiple types and use different update formulas to update the position of each individual. For example, a typical animal-based algorithm may contain various update procedures such as hunting, moving, exploring, and attacking^64^. Some algorithms divide the search process into two phases, exploration and exploitation, but still use different formulas for the update^65^. The exploration phase searches in the global scope, thus preventing the algorithm from converging to a local optimal solution. The exploitation phase is used to perform a local search around the already found solution, thus obtaining the local optimal solution. It has been proved by a large number of algorithms that dividing the algorithm into these two cases can effectively enhance the efficiency of the search. Therefore, in symmetric projection optimization, we also split the search into two phases, but different from the conventional method, we use the same set of formulas to update them and only differentiate in the selection of points. The specific update formulas are:

18 $$ \left\{ \begin{aligned} & X_{3} = SP(\omega ,X_{i}^{loop} ,X_{rand}^{\prime} ,X_{2} ,fit(X_{i} ),fit(X_{rand}^{\prime} ),fit(X_{2} )) \hfill \\ & fit_{i}^{loop + 1} = \min (fit(X_{i} ),fit(X_{2} ),fit(X_{3} )) \hfill \\ & X_{i}^{loop + 1} = X_{i} ,fit_{i}^{loop + 1} { = = }fit(X_{i} ) \hfill \\ & X_{i}^{loop + 1} = X_{2} ,fit_{i}^{loop + 1} { = = }fit(X_{2} ) \hfill \\ & X_{i}^{loop + 1} = X_{3} ,fit_{i}^{loop + 1} { = = }fit(X_{3} ) \hfill \\ \end{aligned} \right. $$

where

19 $$ X_{1} { = }\left\{ {\begin{array}{*{20}l} {X_{rand} + r \cdot (rand - 0.5) \cdot (ub - lb)} & {rand \le ep} \\ {X_{i} + r \cdot (rand - 0.5) \cdot (ub - lb)} & {rand > ep} \\ \end{array} } \right. $$

and

20 $$ \omega = \frac{\pi }{{\sqrt {\sum\limits_{i = 1}^{n} {(ub_{i} - lb_{i} )^{2} } } }} $$

21 $$ r = \frac{1.6}{{loop}} \cdot \frac{{1 + \sqrt {Dim} }}{{1 + e^{{10 \cdot (\frac{loop}{{Maxloop}} - \frac{1}{4})}} }} $$

22 $$ ep = \frac{0.92}{{1 + e^{{1.6 \cdot (loop - \frac{1.4}{{Maxloop}}) \cdot Maxloop}} }} $$

**Figure 4:**
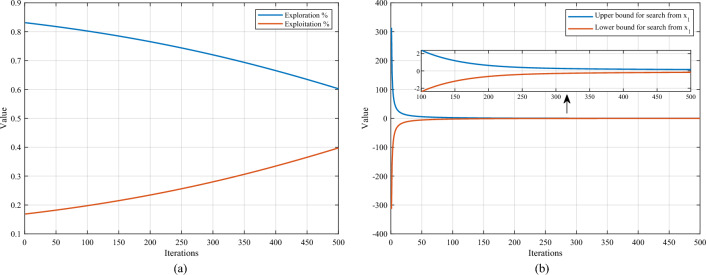
Convergence curve graphs with increasing number of iterations. (**a**) *ep* value. (**b**) *r* value.

For the *x*_*i,*_ whose coordinates are to be updated, an arbitrary known point is chosen in the exploratory phase, and then a random point is selected around this point by Eq. (19). If in the exploitation phase, start with *x*_*i*_ and pick a random point around this. The parameter *ep* controls the selection of modes. As shown in Fig. 4a, as the number of iterations increases, the percentage of points in the exploratory phase gradually decreases, while the rate of points in the development phase gradually increases. In this way, the efficiency and avoiding local optimum can be balanced well. Since more regions or projection surfaces are not explored in the pre-iteration period, more individuals need to be devoted to exploring unknown regions or projection surfaces to find more promising regions. As the number of iterations increases, the more suitable regions that have been identified need to be explored to find the optimal result. Therefore, as the number of iterations increases, the proportion of individuals using the exploration strategy gradually decreases, and the proportion of individuals using the exploitation strategy gradually increases.

The parameter *r* controls the range of selected points. From Fig. 4b, it can be seen that the chosen range converges rapidly with the number of iterations and then maintains a slower rate to continue the convergence. After determining the second point *x*_1_, the third point can be calculated using Eqs. (8) and (15). Then, the fundamental wave with period *ω* fits the function. Much practice has proved that choosing the period parameter through Eq. (21) is more appropriate, as it can effectively estimate the global and local variations.

The pseudocode of SPO is provided in Algorithm 1.

**Algorithm 1:**
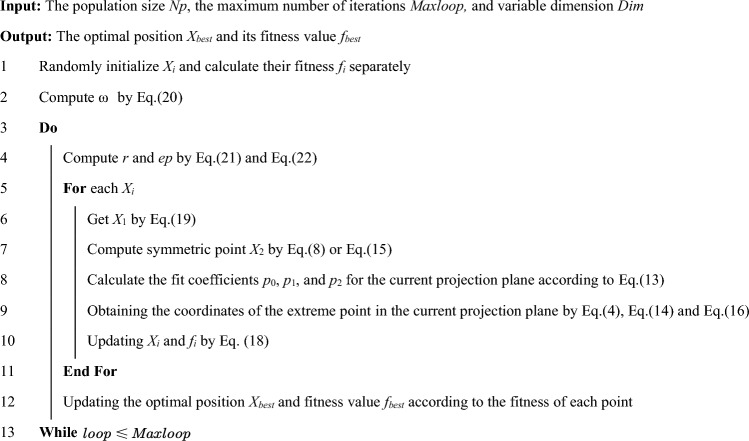
Symmetric projection optimizer

### Computational complexity analysis

For heuristic algorithms, the complexity is mainly related to the size of the population *Np*, the dimensions of the independent variable *dim*, the number of iterations *maxloop,* and the number of its parameters. The computational complexity of the SPO method is significantly reduced compared to other algorithms since the optimal position is updated only by a 1-dimensional projection at each update.

1. Time complexity
  The time complexity of the SPO method consists of three main components: the initialization of the population, the fitness calculation, and the position update. In the initialization process, its time complexity is mainly related to the population size, which is O(*Np*). The fitness calculation's time complexity is O(3·*Np*·*Maxloop*) because three new points are calculated for each iteration. Only the functions of the three positions must be compared in position updating, so the time complexity is O(3·*Np*·*Maxloop*). In summary, the time complexity of the SPO method is
  23 $$ \begin{aligned} O(SPO) & = O(initialization) + O(function{\kern 1pt} {\kern 1pt} evaluation) + O(position{\kern 1pt} {\kern 1pt} updating) \\ & = O\left( {Np} \right) + O\left( {3 \cdot Np \cdot Maxloop} \right) + O\left( {Np \cdot Maxloop} \right) \\ & \approx O\left( {4 \cdot Np \cdot Maxloop} \right) \\ \end{aligned} $$
2. Space complexity
  The SPO method must use O(*Np*·*Dim*) space to save the current population's position. At the same time, it needs to use O(*Np*·*Maxloop*) space to save the optimal position during the whole iteration process. Therefore, the space complexity of the SPO method is
  24 $$ \begin{aligned} O(SPO) & = O(current{\kern 1pt} {\kern 1pt} population) + O(optimal{\kern 1pt} {\kern 1pt} position) \\ & = O\left( {Np \cdot dim} \right) + O\left( {dim \cdot Maxloop} \right) + O\left( {Np \cdot Maxloop} \right) \\ & = O\left( {\left( {Np + Maxloop} \right) \cdot dim} \right) \\ \end{aligned} $$

## Performance tests

In this section, the optimization performance of SPO is verified and evaluated using extensive test suites and compared with the same and different classes of algorithms, respectively. The test suites used in the experimentation and the evaluation criteria are given first. Then, the selected algorithms in each set are introduced separately. Finally, the experiments' results are given, and the convergence performance and stability of the SPO algorithm are quantitatively and qualitatively analysed from several perspectives.

### Experimental design

To verify the performance of the SPO method more comprehensively, the CEC2017, CEC2019, CEC2020, and CEC2022 test suites are selected for comprehensive testing in this section, and the CEC2017 suite is tested with 30, 50, and 100 dimensions, respectively, and the CEC2022 is also tested with 10 and 20 dimensions.

The CEC2017 test suite includes 30 single-objective optimization functions, including three unimodal functions, seven simple multimodal functions, ten hybrid functions, and ten composition functions. Compared with the standard test functions, the CEC2017 test functions are more complex and test the algorithms' optimization capabilities well. At the same time, the challenge of solving these functions rises gradually as the dimensionality increases. The CEC2019 test suite contains ten single-objective test functions, and the dimensionality of the variables in each function is fixed. CEC2020 and CEC2021 test suites use the same test functions, and CEC2020 is chosen for testing in this paper. CEC2020 also contains ten unimodal functions, and 20 dimensions have been selected for the testing. CEC2022 has 12 unimodal functions. There are one unimodal function, four basic functions, three hybrid functions, and four composition functions. Compared to other test suites, the CEC2022 test functions are more traditional.

As seen from the above presentation, the seven types of test suites chosen encompass the vast majority of cases, thus allowing for a comprehensive evaluation of the algorithms from various perspectives. In order to minimize the random factor in the tests, 50 rounds of tests were conducted in the experiment.

#### The mathematical-based algorithms selected for comparisons (MBAs)

Firstly, some mathematical-based algorithms with excellent results in recent years were selected to test the above test suite. The specific parameter settings are shown in Table 3. The selected algorithms include:

- Sine Cosine Algorithm (SCA)^66^: The sine and cosine functions are introduced into the metaheuristic algorithm, and the search for the fitness function is realized by fluctuating outward through the mathematical models of the sine and cosine functions. The experiment proved that the search effect of SCA on 19 basic functions outperforms the other six algorithms.
- RUN beyond the metaphor (RUN)^67^: The Runge Kutta algorithm for integral operations is introduced into the metaheuristic algorithm, which searches for the fitness function by changing the slope. RUN has been experimentally proven to be more efficient than the other eight algorithms on the CEC2017 test suite.
- Arithmetic Optimization Algorithm (AOA)^54^: Four basic operations are introduced into the metaheuristic algorithm, and the search for fitness functions is realized by four operations: addition, subtraction, multiplication, and division. Experiments prove that the search effect of AOA on 29 test functions outperforms the other 11 algorithms.
- Weighted mean of vectors (INFO)^68^: Weighted mean of vectors is introduced into the metaheuristic algorithm, and it searches for fitness functions by composition functions between vectors. Experiments demonstrate that INFO outperforms the other six algorithms on the CEC2017 test suite.
- Sinh Cosh Optimizer (SCHO)^69^: The hyperbolic sine and hyperbolic cosine functions are introduced into the metaheuristic algorithm, and the properties of hyperbolic sine and hyperbolic cosine functions realize the search for the fitness function. Experiments prove that the search effect of SCHO on the CEC2014 test suite is better than the other eight algorithms.
- Exponential distribution optimizer (EDO)^70^: The exponential probability distribution model is introduced into the metaheuristic algorithm, and the exponential distribution model simulation searches the optimization strategy. Experiments prove EDO has some advantages over the other ten algorithms in CEC2014, CEC2017, CEC2020, and CEC2022.
- Triangulation topology aggregation optimizer (TTAO)^71^: A similar triangle topology from mathematics is introduced into the metaheuristic algorithm. Construct multiple topological units through generalized aggregation and local aggregation to enable the search of fitness functions. Experiments demonstrate that TTAO has the best results on average on the CEC2017 test suite compared to ten other algorithms.
- Quadratic Interpolation Optimization (QIO)^56^: The quadratic interpolation to find the minimum value method is introduced into the metaheuristic algorithm, and the search for the optimal position is realized by interpolating three points in each direction separately. Experiments prove that the search effect of QIO is better than the other 12 algorithms on the CEC2014 test suite.

**Table 3:** Parameter settings of MBAs.

Method	Proposed year	Parameters	Value
All		Population size (Np)	30
		Maximum number of iterations (Maxloop)	500
SCA	2016	a	2
RUN	2021	a	1
		b	2
AOA	2021	$\boldsymbol{\alpha }$	5
		${\varvec{\mu}}$	0.5
INFO	2022	c	2
		d	4
SCHO	**2023**	${\varvec{c}}{\varvec{t}},{\varvec{u}},{\varvec{m}}$	3.6,0.388,0.45
		${\varvec{\epsilon}},{\varvec{n}}$	0.003,0.5
		$\boldsymbol{\alpha },{\varvec{\beta}}$	4.6,1.55
		${\varvec{p}},{\varvec{q}}$	10,9
EDO	**2023**	Switch parameter	0.5
TTAO	**2023**	${{\varvec{r}}}_{0},{{\varvec{r}}}_{1},{{\varvec{r}}}_{2},{{\varvec{r}}}_{3},{{\varvec{r}}}_{4}$	[0,1]
QIO	**2023**		

#### The other-based algorithms selected for comparisons (OBAs)

Two widely used metaheuristic algorithms and seven recently introduced algorithms were chosen for testing in the second set of experiments. The specific parameter settings are shown in Table 4. The chosen algorithms include:

- Particle Swarm Optimization (PSO)^29^: The animal-based metaheuristic algorithm. The algorithm has been widely used in practical engineering, which proves its reliability and practicality. The test results of the PSO algorithm can be used as a benchmark for comparison.
- Artificial gorilla troops optimizer (GTO)^72^: The animal-based metaheuristic algorithm. The optimization space is searched by simulating collective life among gorillas. Experiments prove that the performance of GTO outperforms eight other algorithms on 52 test functions, such as CEC2017. It has been widely used in practical engineering in recent years.
- Dandelion Optimizer (DO)^36^: The natural-based metaheuristic algorithm. The search for the optimization space is realized by simulating the flight process of dandelion seeds. Experiments demonstrate that the DO outperforms the other nine algorithms on the CEC2017 test suite.
- Snake Optimizer (SO)^73^: The animal-based algorithm. The search is achieved by simulating the behaviors of snakes, such as predation and mating. Experiments demonstrate that SO is superior to the other nine algorithms on the CEC2017 test suite.
- Fick's Law Algorithm (FLA)^49^: The physics-based metaheuristic algorithm. The search of the optimization space is implemented using Fick's diffusion law. Experiments demonstrate that the search performance of FLA outperforms the other 12 algorithms on the CEC2017 test suite.
- Human Evolutionary Optimization Algorithm (HEOA)^40^: The human-based metaheuristic algorithm. The optimization space is searched by simulating human behavior during global search. Experiments demonstrate that the search efficiency of HEOA outperforms the other ten algorithms on 23 test functions.
- Kepler Optimization Algorithm (KOA)^74^: The physics-based metaheuristic algorithm. The optimization space is searched by updating the candidate solutions using Kepler motion laws. Experiments show that KOA is more efficient than the other 12 algorithms on four test suites, including CEC2014, CEC2017, CEC2020, and CEC2022.
- Young's double-slit experiment optimizer (YDSE)^48^: The physics-based metaheuristic algorithm. The search of the search space is achieved by simulating the behavior of light in Young's double-slit experiment. Experiments prove that the optimization performance of YDSW outperforms the other 12 algorithms on CEC2014, CE2017, and CEC2022.
- Genghis Khan shark optimizer (GKSE)^75^: The animal-based metaheuristic algorithm. The search for the optimal position is achieved by simulating the predation process of Genghis Khan sharks. Experiments demonstrate that GKSE is more substantial than the other eight fish algorithms and the other nine algorithms on two test suites, CEC2019 and CEC2022.

**Table 4:** Parameter settings of OBAs.

Method	Proposed year	Parameters	Value
All		Population size (Np)	30
		Maximum number of iterations (Maxloop)	500
PSO	1995	Inertia factor	[0.2,0.9]
		*vMax*	0.2 × (ub-lb)
		c_1_	2
		c_2_	2
GTO	2021	${\varvec{\beta}}$	3
		W	0.8
		*p*	0.03
DO	2022	$\boldsymbol{\alpha }$	[0,1]
		*k*	[0,1]
SO	2022	c_1_	0.5
FLA	2022	c_1_	1
		c_2_	2
		c_3_	0.1
		c_4_	0.2
		c_5_	2
HEOA	**2023**	–	–
KOA	**2023**	Number of cycles $\overline{{\varvec{T}} }$	3
		Depth of the parameter ${\varvec{\gamma}}$	15
		The beginning value for the parameter ${{\varvec{\mu}}}_{0}$	0.1
YDSE	**2023**	Wavelength (${\varvec{\lambda}}$)	5 × 10^−6^
		Distance between two slits (d)	5 × 10^−3^
		Distance between the barrier and the projection screen (L)	1
		Distance between light source and barrier (I)	0.01
		Constant value (δ)	0.38
GKSO	**2023**	*m*	1.5

#### Performance indices

1. *Mean*_*i*_ refers to the mean fitness of the algorithm after 50 tests. Since heuristic algorithms are mostly randomly initialized, certain exceptional cases make the algorithm's performance demonstrate better or worse than the actual performance. Therefore, the mean fitness is usually taken to evaluate the algorithm's capability. Its calculation formula is
  25 $$ Mean_{i} = \frac{1}{50}\sum\limits_{n = 1}^{50} {fitness_{i}^{n} } $$
2. *Std*_*i*_ refers to the standard deviation of all best results of the algorithm after 50 tests. The smaller the standard deviation, the better the stability of the algorithm. Its calculation formula is
  26 $$ Std_{i} = \sqrt {\frac{1}{49}\sum\limits_{n = 1}^{50} {\left( {fitness_{i}^{n} - Mean_{i} } \right)^{2} } } $$
3. *MeanRank*_*i*_ refers to the mean ranking of the algorithm on each test function of the current test suite. The mean rank measures the algorithm's overall performance on the test suite. Its calculation formula is
  27 $$ MeanRank_{i} = \frac{1}{k}\sum\limits_{k = 1}^{K} {rank_{i}^{k} } $$
4. Wilcoxon's rank sum test^76^ is a non-parametric hypothesis testing method mainly used to test whether the distributions of two data sets are the same. In this paper, the 50 experimental results of the SPO algorithm are rank-sum tested against the 50 test results of other algorithms. If the two fitness sets do not satisfy the rank sum test, that proves the SPO algorithm has a significant advantage over the comparison algorithm.
5. The Friedman test is a multiple comparison test that compares the performance of several algorithms simultaneously by using various functions. Its formula is shown in Eq. (28).
  28 $$ FT = \frac{12N}{{K(K + 1)}}\left( {\sum\limits_{k = 1}^{K} {R_{k}^{2} } - \frac{{K(K + 1)^{2} }}{4}} \right) $$
  where *N* is the number of test functions, *K* is the number of algorithms, and *R*_*k*_ is the average ranking of the kth algorithm. For the Friedman test with degree of freedom 1, the smaller the final value obtained, the better the algorithm's performance.

### General performance analysis

**Figure 5:**
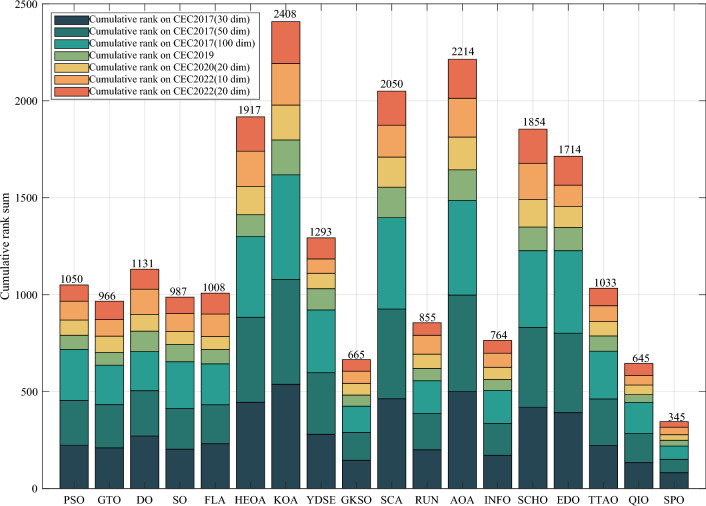
Comparison of the cumulative rank sum of all algorithms on all tests.

Table 5 and Fig. 5 show the cumulative rank of two sets of algorithms in each test suit. It can be seen that the SPO algorithm has the best cumulative ranking in all tests. On the CEC2017 test suite, the cumulative rank of SPO decreases as the dimensionality of the variables increases. The cumulative rank was 82 when the variable dimension was 30 dimensions and 69 when the variable dimension was 100 dimensions, a 15.8% decrease in cumulative rank. The same occurred on the CEC2022 test suite, where the cumulative rank decreased by 23.7%. The results indicate that as the dimensionality increases, the performance of the SPO algorithm improves significantly compared to the other algorithm.

**Table 5:** Cumulative rank across all tests.

Methods		CEC2017 (30 dim)	CEC2017 (50 dim)	CEC2017 (100 dim)	CEC2019	CEC2020 (20 dim)	CEC2022 (10 dim)	CEC2022 (20 dim)	Mean rank	Final ranking
OBAs	PSO	223	232	262	73	79	97	84	7.74	9
	GTO	210	223	203	66	84	85	95	7.31	6
	DO	271	234	201	106	86	130	103	8.88	11
	SO	203	210	241	90	66	92	85	7.45	7
	FLA	231	201	211	74	68	115	108	7.75	10
	HEOA	445	438	417	113	145	182	177	14.15	15
	KOA	538	540	540	180	180	214	216	17.97	18
	YDSE	280	318	323	110	79	74	109	9.26	12
	GKSO	146	144	135	57	60	63	60	5.16	3
MBAs	SCA	463	463	471	157	156	164	176	15.17	16
	RUN	200	187	169	64	73	97	65	6.53	5
	AOA	500	498	488	158	169	200	201	16.52	17
	INFO	171	164	171	56	63	73	66	5.76	4
	SCHO	419	412	396	122	141	187	177	13.93	14
	EDO	392	409	426	119	109	110	149	12.18	13
	TTAO	222	240	246	79	74	82	90	7.60	8
	QIO	134	149	161	41	49	49	62	4.72	2
	SPO	82	68	69	30	29	38	29	2.68	1

From the overall ranks, the SPO algorithm is ranked first in all test suites with a mean rank value of 2.68, meaning that in most cases, the SPO algorithm is ranked in the top three regarding search performance on all test functions. The algorithm with the second-best mean rank is QIO, which has a mean rank value of 4.72. The mean rank value of the SPO algorithm is 43.2% lower compared to QIO, which can be considered a significant advantage compared to the second place.

### Quantitative analysis

#### Comparative analysis of the SPO algorithm and the other MBAs

Tables 6, 7, 8, 9, 10, 11,12 show the results of the SPO algorithm and other MBAs in different test suites. The best result has been marked in bold.

**Table 6:** Comparison of results between MBAs and SPO on CEC2017 with 30 dimensional.

F	Type	SCA	RUN	AOA	INFO	SCHO	EDO	TTAO	QIO	SPO
F1	Mean	2.06E+10	1.50E+04	5.33E+10	8.29E+05	1.42E+10	2.41E+10	2.35E+06	2.09E+06	**1.97E+03**
	Std	3.82E+09	9.24E+03	9.46E+09	5.55E+06	6.31E+09	7.13E+09	2.27E+06	1.74E+06	1.12E+03
F3	Mean	8.60E+04	9.31E+03	8.18E+04	1.60E+04	8.31E+04	4.40E+04	5.51E+04	5.54E+04	**7.58E+03**
	Std	1.89E+04	2.79E+03	8.04E+03	5.19E+03	1.34E+04	8.55E+03	1.48E+04	1.03E+04	2.99E+03
F4	Mean	2.88E+03	5.13E+02	1.60E+04	**5.06E+02**	2.32E+03	3.14E+03	5.30E+02	5.29E+02	5.09E+02
	Std	6.59E+02	1.85E+01	5.72E+03	3.22E+01	1.41E+03	1.30E+03	3.07E+01	2.96E+01	1.42E+01
F5	Mean	8.34E+02	7.06E+02	8.94E+02	6.67E+02	7.70E+02	8.31E+02	7.08E+02	6.20E+02	**5.74E+02**
	Std	2.49E+01	3.74E+01	3.55E+01	4.13E+01	3.80E+01	3.37E+01	4.33E+01	3.15E+01	1.19E+01
F6	Mean	6.63E+02	6.49E+02	6.80E+02	6.29E+02	6.61E+02	6.67E+02	6.43E+02	**6.17E+02**	6.18E+02
	Std	6.73E+00	7.01E+00	8.07E+00	8.79E+00	1.26E+01	9.45E+00	1.38E+01	7.13E+00	6.77E+00
F7	Mean	1.26E+03	1.07E+03	1.40E+03	9.93E+02	1.16E+03	1.24E+03	9.42E+02	9.07E+02	**7.93E+02**
	Std	5.82E+01	6.26E+01	5.76E+01	6.28E+01	8.38E+01	6.46E+01	5.08E+01	4.86E+01	1.27E+01
F8	Mean	1.10E+03	9.51E+02	1.12E+03	9.21E+02	1.03E+03	1.09E+03	9.72E+02	9.05E+02	**8.61E+02**
	Std	2.29E+01	2.37E+01	3.35E+01	3.25E+01	4.41E+01	2.27E+01	2.89E+01	2.03E+01	1.09E+01
F9	Mean	8.67E+03	4.06E+03	7.77E+03	3.30E+03	1.20E+04	8.99E+03	6.01E+03	2.59E+03	**1.34E+03**
	Std	1.69E+03	6.26E+02	1.22E+03	6.79E+02	4.55E+03	1.70E+03	2.30E+03	1.11E+03	2.66E+02
F10	Mean	8.92E+03	5.20E+03	7.90E+03	5.29E+03	6.99E+03	7.82E+03	5.98E+03	6.56E+03	**4.37E+03**
	Std	3.19E+02	1.32E+03	6.30E+02	9.07E+02	6.43E+02	4.36E+02	4.50E+02	1.62E+03	3.77E+02
F11	Mean	4.07E+03	**1.21E+03**	9.48E+03	1.28E+03	3.97E+03	1.82E+03	1.26E+03	1.24E+03	1.24E+03
	Std	8.98E+02	3.35E+01	2.79E+03	6.46E+01	1.79E+03	2.03E+02	4.96E+01	4.77E+01	3.06E+01
F12	Mean	2.75E+09	6.76E+06	1.45E+10	**7.98E+05**	1.40E+09	4.35E+08	2.83E+06	2.67E+06	2.99E+06
	Std	8.44E+08	3.68E+06	4.21E+09	7.28E+05	1.71E+09	2.26E+08	2.36E+06	2.06E+06	2.23E+06
F13	Mean	1.25E+09	2.61E+04	1.43E+10	2.24E+04	6.92E+08	7.90E+06	1.97E+04	**9.79E+03**	2.04E+04
	Std	5.36E+08	1.34E+04	6.34E+09	2.36E+04	1.59E+09	5.02E+06	1.57E+04	9.36E+03	5.09E+03
F14	Mean	1.04E+06	2.42E+04	6.65E+06	7.74E+03	8.27E+05	2.56E+03	2.55E+04	1.50E+04	**1.94E+03**
	Std	8.25E+05	1.87E+04	1.94E+07	7.43E+03	8.36E+05	6.47E+02	2.16E+04	2.08E+04	3.49E+02
F15	Mean	5.89E+07	1.43E+04	4.24E+08	9.04E+03	5.97E+06	6.94E+04	6.06E+03	**5.77E+03**	8.18E+03
	Std	4.29E+07	2.46E+03	4.67E+08	8.73E+03	1.38E+07	5.25E+04	5.10E+03	5.92E+03	2.00E+03
F16	Mean	4.14E+03	2.89E+03	5.11E+03	2.73E+03	3.32E+03	3.93E+03	2.97E+03	2.60E+03	**2.53E+03**
	Std	2.54E+02	3.26E+02	1.05E+03	2.91E+02	3.81E+02	2.69E+02	2.52E+02	3.05E+02	1.60E+02
F17	Mean	2.82E+03	2.24E+03	4.78E+03	2.38E+03	2.54E+03	2.67E+03	2.20E+03	2.06E+03	**1.93E+03**
	Std	1.65E+02	2.37E+02	2.28E+03	2.29E+02	2.07E+02	1.62E+02	1.63E+02	1.92E+02	8.09E+01
F18	Mean	1.44E+07	1.24E+05	4.78E+07	1.50E+05	9.12E+06	1.08E+05	3.11E+05	2.87E+05	**4.04E+04**
	Std	1.02E+07	6.05E+04	1.01E+08	1.77E+05	1.09E+07	6.18E+04	2.04E+05	2.90E+05	1.50E+04
F19	Mean	1.18E+08	3.53E+04	4.78E+08	1.05E+04	3.20E+07	2.85E+05	**7.47E+03**	8.15E+03	6.15E+04
	Std	8.17E+07	6.00E+04	4.34E+08	1.14E+04	9.64E+07	1.68E+05	5.84E+03	7.39E+03	7.94E+04
F20	Mean	2.92E+03	2.53E+03	2.85E+03	2.64E+03	2.86E+03	2.95E+03	2.60E+03	2.41E+03	**2.37E+03**
	Std	1.66E+02	1.43E+02	1.96E+02	1.93E+02	2.75E+02	1.59E+02	1.28E+02	1.54E+02	8.50E+01
F21	Mean	2.61E+03	2.45E+03	2.68E+03	2.43E+03	2.57E+03	2.60E+03	2.47E+03	2.39E+03	**2.37E+03**
	Std	2.51E+01	5.09E+01	5.39E+01	3.21E+01	4.86E+01	2.88E+01	3.23E+01	2.66E+01	1.06E+01
F22	Mean	9.83E+03	3.57E+03	9.01E+03	4.86E+03	7.87E+03	6.23E+03	3.84E+03	2.31E+03	**2.30E+03**
	Std	1.51E+03	2.10E+03	6.71E+02	2.17E+03	1.54E+03	1.67E+03	2.34E+03	3.00E+00	3.88E−01
F23	Mean	3.08E+03	2.79E+03	3.59E+03	2.84E+03	3.06E+03	3.14E+03	2.85E+03	2.78E+03	**2.74E+03**
	Std	4.38E+01	4.35E+01	2.09E+02	5.61E+01	8.31E+01	6.89E+01	5.20E+01	3.69E+01	2.33E+01
F24	Mean	3.25E+03	2.96E+03	3.89E+03	2.99E+03	3.29E+03	3.28E+03	3.01E+03	2.96E+03	**2.89E+03**
	Std	3.54E+01	3.65E+01	2.24E+02	5.67E+01	9.30E+01	6.36E+01	4.86E+01	3.74E+01	1.71E+01
F25	Mean	3.59E+03	2.92E+03	5.68E+03	**2.91E+03**	3.28E+03	3.54E+03	2.94E+03	2.92E+03	2.94E+03
	Std	2.63E+02	2.62E+01	9.18E+02	2.29E+01	1.96E+02	2.53E+02	2.56E+01	2.29E+01	1.04E+01
F26	Mean	7.85E+03	5.95E+03	1.09E+04	6.08E+03	7.47E+03	7.25E+03	5.71E+03	4.84E+03	**3.92E+03**
	Std	4.64E+02	1.42E+03	9.83E+02	1.10E+03	8.55E+02	8.12E+02	1.11E+03	1.16E+03	1.04E+03
F27	Mean	3.55E+03	3.29E+03	4.67E+03	3.26E+03	3.57E+03	3.35E+03	3.28E+03	3.27E+03	**3.25E+03**
	Std	7.22E+01	3.42E+01	3.80E+02	3.95E+01	1.35E+02	4.47E+01	2.95E+01	2.55E+01	1.42E+01
F28	Mean	4.56E+03	3.25E+03	6.95E+03	**3.24E+03**	4.08E+03	4.37E+03	3.28E+03	3.29E+03	3.26E+03
	Std	5.14E+02	2.21E+01	8.99E+02	2.42E+01	4.65E+02	4.38E+02	2.49E+01	2.73E+01	2.10E+01
F29	Mean	5.29E+03	4.32E+03	7.89E+03	4.13E+03	4.60E+03	4.83E+03	4.22E+03	**3.85E+03**	3.92E+03
	Std	2.99E+02	2.79E+02	2.41E+03	2.41E+02	3.49E+02	2.70E+02	2.52E+02	2.15E+02	1.26E+02
F30	Mean	2.01E+08	6.06E+05	2.31E+09	**1.81E+04**	4.45E+07	5.87E+06	4.89E+04	2.46E+04	2.96E+05
	Std	9.18E+07	6.57E+05	1.24E+09	1.20E+04	1.48E+08	4.53E+06	4.91E+04	1.78E+04	2.48E+05
Mean rank		7.83	3.57	8.67	3.2	6.7	6.47	4.07	2.73	**1.77**
Final ranking		8	4	9	3	7	6	5	2	1
+/=/−		29/0/0	25/0/4	29/0/0	23/0/6	29/0/0	29/0/0	24/0/5	21/0/8	

+ indicates that SPO obtains better results than others, = indicates that SPO obtains equal results than others, − indicates that SPO is worse than others.

**Table 7:** Comparison of results between MBAs and SPO on CEC2017 with 50 dimensional.

F	Type	SCA	RUN	AOA	INFO	SCHO	EDO	TTAO	QIO	SPO
F1	Mean	6.69E+10	1.58E+06	1.11E+11	2.14E+08	3.76E+10	7.66E+10	3.63E+07	1.68E+08	**5.38E+04**
	Std	6.91E+09	1.12E+06	1.07E+10	6.02E+08	8.92E+09	1.14E+10	1.42E+07	5.41E+07	1.26E+04
F3	Mean	2.18E+05	**6.09E+04**	1.83E+05	1.09E+05	2.40E+05	1.38E+05	1.71E+05	1.62E+05	6.18E+04
	Std	3.86E+04	1.25E+04	2.43E+04	2.85E+04	4.41E+04	2.04E+04	3.19E+04	2.26E+04	1.11E+04
F4	Mean	1.36E+04	6.36E+02	3.28E+04	6.43E+02	6.78E+03	1.90E+04	7.09E+02	7.47E+02	**6.34E+02**
	Std	3.03E+03	5.15E+01	7.66E+03	6.54E+01	2.00E+03	3.50E+03	4.71E+01	8.05E+01	3.40E+01
F5	Mean	1.13E+03	8.36E+02	1.18E+03	8.17E+02	1.02E+03	1.12E+03	9.60E+02	7.69E+02	**6.66E+02**
	Std	3.34E+01	2.64E+01	4.70E+01	5.11E+01	5.52E+01	3.92E+01	6.67E+01	3.80E+01	1.91E+01
F6	Mean	6.84E+02	6.59E+02	6.97E+02	6.44E+02	6.79E+02	6.90E+02	6.64E+02	6.34E+02	**6.34E+02**
	Std	6.66E+00	4.98E+00	6.39E+00	8.96E+00	1.11E+01	7.95E+00	1.28E+01	7.35E+00	5.40E+00
F7	Mean	1.89E+03	1.49E+03	1.98E+03	1.41E+03	1.68E+03	1.87E+03	1.28E+03	1.18E+03	**9.57E+02**
	Std	1.07E+02	1.04E+02	6.66E+01	1.17E+02	1.29E+02	9.45E+01	8.72E+01	9.91E+01	3.06E+01
F8	Mean	1.45E+03	1.14E+03	1.51E+03	1.12E+03	1.34E+03	1.42E+03	1.23E+03	1.06E+03	**9.63E+02**
	Std	2.91E+01	2.97E+01	4.08E+01	5.62E+01	6.66E+01	3.48E+01	6.31E+01	3.80E+01	1.95E+01
F9	Mean	3.19E+04	1.22E+04	3.20E+04	9.55E+03	4.31E+04	3.48E+04	2.68E+04	1.38E+04	**5.18E+03**
	Std	4.95E+03	4.06E+03	3.48E+03	1.92E+03	9.54E+03	3.85E+03	6.47E+03	6.22E+03	1.03E+03
F10	Mean	1.54E+04	8.29E+03	1.39E+04	8.42E+03	1.27E+04	1.47E+04	1.11E+04	1.10E+04	**7.89E+03**
	Std	5.37E+02	1.93E+03	6.33E+02	9.71E+02	1.03E+03	9.04E+02	6.37E+02	2.92E+03	5.72E+02
F11	Mean	1.34E+04	**1.36E+03**	2.42E+04	1.45E+03	1.13E+04	7.55E+03	1.58E+03	1.58E+03	1.49E+03
	Std	2.90E+03	6.22E+01	3.66E+03	1.44E+02	3.36E+03	2.85E+03	1.12E+02	1.65E+02	6.71E+01
F12	Mean	2.18E+10	4.29E+07	7.83E+10	6.20E+07	1.27E+10	1.23E+10	4.23E+07	**3.09E+07**	3.94E+07
	Std	4.38E+09	2.89E+07	1.59E+10	3.11E+08	7.79E+09	7.99E+09	2.64E+07	1.75E+07	1.50E+07
F13	Mean	6.72E+09	3.64E+04	4.37E+10	3.08E+04	4.04E+09	7.78E+08	1.67E+05	**1.56E+04**	3.16E+04
	Std	2.19E+09	1.33E+04	1.47E+10	2.94E+04	4.63E+09	4.89E+08	1.52E+05	8.41E+03	7.59E+03
F14	Mean	8.62E+06	7.72E+04	1.17E+08	1.19E+05	5.59E+06	1.48E+05	3.05E+05	2.56E+05	**1.76E+04**
	Std	4.41E+06	5.11E+04	9.44E+07	1.12E+05	5.98E+06	1.03E+05	2.17E+05	3.53E+05	1.22E+04
F15	Mean	1.09E+09	2.38E+04	8.73E+09	1.24E+04	6.36E+08	2.34E+07	2.39E+04	**6.42E+03**	1.10E+04
	Std	4.22E+08	4.20E+03	3.82E+09	6.98E+03	1.02E+09	1.43E+07	2.62E+04	4.73E+03	3.76E+03
F16	Mean	6.38E+03	3.54E+03	8.34E+03	3.53E+03	4.72E+03	6.02E+03	3.97E+03	3.31E+03	**2.97E+03**
	Std	4.53E+02	5.16E+02	1.43E+03	4.85E+02	6.52E+02	3.95E+02	4.86E+02	4.06E+02	2.80E+02
F17	Mean	5.16E+03	3.35E+03	2.00E+04	3.34E+03	4.08E+03	4.42E+03	3.21E+03	3.05E+03	**2.82E+03**
	Std	3.41E+02	2.88E+02	1.27E+04	4.26E+02	6.59E+02	2.60E+02	2.55E+02	3.22E+02	2.15E+02
F18	Mean	5.79E+07	7.58E+05	1.79E+08	5.92E+05	2.23E+07	1.63E+06	2.36E+06	1.80E+06	**2.43E+05**
	Std	2.79E+07	3.47E+05	1.20E+08	4.29E+05	2.22E+07	9.99E+05	1.78E+06	1.41E+06	1.66E+05
F19	Mean	6.19E+08	1.86E+05	5.81E+09	2.19E+04	2.39E+08	1.21E+07	2.12E+04	**1.65E+04**	8.34E+04
	Std	3.23E+08	9.21E+04	2.09E+09	1.94E+04	4.73E+08	6.79E+06	1.27E+04	8.44E+03	5.35E+04
F20	Mean	4.29E+03	3.07E+03	4.01E+03	3.40E+03	3.73E+03	3.96E+03	3.24E+03	3.02E+03	**2.88E+03**
	Std	2.08E+02	2.58E+02	2.58E+02	3.15E+02	3.18E+02	2.19E+02	2.03E+02	3.67E+02	1.44E+02
F21	Mean	2.96E+03	2.62E+03	3.10E+03	2.62E+03	2.87E+03	2.99E+03	2.68E+03	2.54E+03	**2.44E+03**
	Std	3.37E+01	5.37E+01	8.15E+01	5.81E+01	6.55E+01	4.95E+01	7.03E+01	4.73E+01	1.94E+01
F22	Mean	1.72E+04	9.76E+03	1.63E+04	9.97E+03	1.44E+04	1.62E+04	1.26E+04	9.99E+03	**8.26E+03**
	Std	4.54E+02	1.64E+03	6.29E+02	1.03E+03	8.07E+02	6.37E+02	1.59E+03	4.07E+03	3.04E+03
F23	Mean	3.72E+03	3.16E+03	4.47E+03	3.22E+03	3.68E+03	3.75E+03	3.24E+03	3.09E+03	**2.96E+03**
	Std	6.44E+01	6.98E+01	2.67E+02	1.03E+02	1.62E+02	1.22E+02	1.03E+02	6.66E+01	4.10E+01
F24	Mean	3.91E+03	3.23E+03	5.08E+03	3.34E+03	3.88E+03	4.03E+03	3.41E+03	3.28E+03	**3.09E+03**
	Std	8.36E+01	7.37E+01	2.82E+02	1.29E+02	1.36E+02	1.38E+02	9.71E+01	8.25E+01	3.00E+01
F25	Mean	9.15E+03	3.19E+03	1.58E+04	3.18E+03	6.01E+03	9.83E+03	3.20E+03	3.21E+03	**3.14E+03**
	Std	1.40E+03	2.89E+01	1.80E+03	4.75E+01	9.96E+02	1.35E+03	3.96E+01	5.12E+01	2.19E+01
F26	Mean	1.38E+04	1.18E+04	1.70E+04	9.34E+03	1.21E+04	1.36E+04	9.48E+03	8.24E+03	**7.23E+03**
	Std	1.03E+03	9.90E+02	9.63E+02	1.69E+03	1.02E+03	1.20E+03	2.17E+03	2.23E+03	1.55E+03
F27	Mean	4.92E+03	3.86E+03	7.04E+03	3.71E+03	4.73E+03	4.09E+03	3.81E+03	3.70E+03	**3.62E+03**
	Std	2.07E+02	1.21E+02	5.46E+02	1.85E+02	4.26E+02	1.86E+02	1.43E+02	1.19E+02	7.48E+01
F28	Mean	9.00E+03	3.54E+03	1.24E+04	3.54E+03	6.40E+03	9.17E+03	3.54E+03	3.65E+03	**3.43E+03**
	Std	1.05E+03	6.09E+01	1.33E+03	1.36E+02	8.69E+02	1.09E+03	6.43E+01	1.17E+02	4.56E+01
F29	Mean	9.07E+03	5.60E+03	1.47E+05	5.18E+03	7.30E+03	7.62E+03	5.42E+03	**4.61E+03**	4.94E+03
	Std	1.07E+03	5.34E+02	2.39E+05	4.72E+02	1.27E+03	5.65E+02	6.15E+02	4.04E+02	2.90E+02
F30	Mean	1.36E+09	4.98E+07	7.74E+09	**1.93E+06**	6.60E+08	1.75E+08	4.69E+06	4.15E+06	4.19E+07
	Std	6.30E+08	9.98E+06	3.54E+09	7.69E+05	1.01E+09	6.87E+07	2.15E+06	1.27E+06	1.08E+07
Mean rank		7.77	3.4	8.73	3.07	6.53	6.77	4.33	2.93	**1.47**
Final ranking		8	4	9	3	6	7	5	2	1
+/=/−		29/0/0	27/0/2	29/0/0	25/0/4	29/0/0	29/0/0	27/0/2	23/0/6	

+ indicates that SPO obtains better results than others, = indicates that SPO obtains equal results than others, − indicates that SPO is worse than others.

**Table 8:** Comparison of results between MBAs and SPO on CEC2017 with 100 dimensional.

F	Type	SCA	RUN	AOA	INFO	SCHO	EDO	TTAO	QIO	SPO
F1	Mean	2.14E+11	3.30E+09	2.72E+11	1.62E+10	1.23E+11	2.27E+11	1.12E+09	6.09E+09	**8.40E+07**
	Std	1.59E+10	1.59E+09	1.28E+10	7.03E+09	1.50E+10	1.63E+10	1.78E+08	1.09E+09	1.98E+07
F3	Mean	6.20E+05	3.01E+05	3.78E+05	3.65E+05	4.55E+05	3.15E+05	5.15E+05	4.27E+05	**2.80E+05**
	Std	1.12E+05	3.19E+04	5.40E+04	7.53E+04	1.12E+05	2.35E+04	6.46E+04	5.89E+04	2.69E+04
F4	Mean	5.21E+04	1.69E+03	9.45E+04	2.08E+03	1.98E+04	6.53E+04	1.43E+03	2.06E+03	**9.85E+02**
	Std	9.57E+03	2.16E+02	1.50E+04	4.72E+02	5.60E+03	1.48E+04	1.33E+02	3.26E+02	4.07E+01
F5	Mean	2.04E+03	1.33E+03	2.06E+03	1.31E+03	1.83E+03	2.01E+03	1.71E+03	1.32E+03	**1.00E+03**
	Std	7.33E+01	3.06E+01	6.59E+01	8.75E+01	9.86E+01	4.22E+01	1.31E+02	7.05E+01	6.11E+01
F6	Mean	7.05E+02	6.66E+02	7.09E+02	6.60E+02	6.97E+02	7.07E+02	6.87E+02	6.57E+02	**6.49E+02**
	Std	5.71E+00	2.15E+00	4.94E+00	4.50E+00	1.05E+01	4.15E+00	8.70E+00	4.98E+00	4.84E+00
F7	Mean	4.10E+03	2.96E+03	3.98E+03	2.87E+03	3.33E+03	3.74E+03	2.31E+03	2.44E+03	**1.62E+03**
	Std	1.86E+02	1.33E+02	8.01E+01	2.10E+02	2.06E+02	1.17E+02	1.77E+02	2.11E+02	1.06E+02
F8	Mean	2.42E+03	1.77E+03	2.53E+03	1.69E+03	2.20E+03	2.46E+03	2.10E+03	1.69E+03	**1.33E+03**
	Std	7.52E+01	6.60E+01	8.74E+01	8.88E+01	1.14E+02	5.29E+01	1.57E+02	8.10E+01	6.52E+01
F9	Mean	8.84E+04	2.67E+04	7.27E+04	2.63E+04	9.64E+04	7.71E+04	8.08E+04	5.69E+04	**2.24E+04**
	Std	1.05E+04	1.56E+03	6.49E+03	3.00E+03	5.95E+03	3.87E+03	1.11E+04	1.21E+04	2.64E+03
F10	Mean	3.30E+04	**1.78E+04**	3.12E+04	1.82E+04	2.87E+04	3.17E+04	2.57E+04	2.59E+04	1.99E+04
	Std	6.27E+02	3.49E+03	1.23E+03	1.75E+03	1.55E+03	7.89E+02	8.62E+02	5.45E+03	8.38E+02
F11	Mean	1.76E+05	**1.18E+04**	1.71E+05	3.09E+04	1.35E+05	1.30E+05	5.12E+04	7.04E+04	1.31E+04
	Std	3.44E+04	2.82E+03	2.25E+04	8.75E+03	2.67E+04	2.21E+04	1.30E+04	1.36E+04	2.86E+03
F12	Mean	1.05E+11	5.43E+08	1.90E+11	1.19E+09	5.25E+10	1.18E+11	6.31E+08	7.60E+08	**5.36E+08**
	Std	1.19E+10	1.98E+08	1.98E+10	2.19E+09	1.42E+10	2.56E+10	2.23E+08	2.63E+08	7.89E+07
F13	Mean	1.80E+10	3.94E+04	4.65E+10	2.95E+05	1.11E+10	2.22E+10	9.67E+05	6.69E+05	**2.93E+04**
	Std	3.69E+09	7.64E+03	6.63E+09	1.05E+06	4.79E+09	6.93E+09	8.05E+05	4.28E+05	4.08E+03
F14	Mean	6.96E+07	7.27E+05	1.22E+08	1.54E+06	1.63E+07	3.49E+06	3.31E+06	2.17E+06	**5.20E+05**
	Std	2.96E+07	2.50E+05	7.28E+07	6.77E+05	7.75E+06	1.63E+06	1.67E+06	9.90E+05	2.71E+05
F15	Mean	6.25E+09	2.31E+04	2.66E+10	1.79E+04	4.19E+09	4.86E+09	2.34E+05	**1.63E+04**	2.00E+04
	Std	2.25E+09	4.95E+03	4.79E+09	1.10E+04	3.27E+09	2.94E+09	7.11E+05	7.42E+03	3.64E+03
F16	Mean	1.51E+04	7.36E+03	2.14E+04	6.59E+03	1.13E+04	1.49E+04	7.90E+03	**6.19E+03**	6.46E+03
	Std	1.06E+03	7.84E+02	3.41E+03	6.39E+02	1.44E+03	1.56E+03	1.10E+03	7.31E+02	4.92E+02
F17	Mean	6.55E+04	5.59E+03	7.87E+06	6.16E+03	3.87E+04	2.82E+04	5.87E+03	5.13E+03	**4.39E+03**
	Std	9.40E+04	5.17E+02	8.81E+06	7.35E+02	5.77E+04	4.33E+04	7.66E+02	4.45E+02	3.55E+02
F18	Mean	1.41E+08	1.25E+06	2.11E+08	2.48E+06	1.89E+07	5.60E+06	4.81E+06	3.74E+06	**7.01E+05**
	Std	6.70E+07	5.04E+05	1.19E+08	1.55E+06	1.08E+07	2.99E+06	2.52E+06	1.75E+06	2.82E+05
F19	Mean	5.66E+09	9.74E+05	2.38E+10	4.96E+04	2.94E+09	5.16E+09	4.03E+05	**3.03E+04**	8.26E+05
	Std	1.54E+09	6.72E+05	5.54E+09	5.61E+04	2.99E+09	3.29E+09	4.23E+05	1.81E+04	1.12E+06
F20	Mean	8.04E+03	5.25E+03	7.56E+03	5.70E+03	6.78E+03	7.78E+03	5.94E+03	5.45E+03	**4.78E+03**
	Std	3.08E+02	5.48E+02	2.56E+02	5.68E+02	5.25E+02	3.17E+02	3.29E+02	7.84E+02	2.96E+02
F21	Mean	4.20E+03	3.16E+03	4.74E+03	3.31E+03	4.03E+03	4.25E+03	3.50E+03	3.16E+03	**2.78E+03**
	Std	1.10E+02	1.16E+02	1.68E+02	1.48E+02	1.35E+02	1.28E+02	1.47E+02	9.67E+01	5.89E+01
F22	Mean	3.55E+04	2.44E+04	3.41E+04	**2.15E+04**	3.21E+04	3.45E+04	2.89E+04	2.67E+04	2.16E+04
	Std	5.72E+02	5.11E+03	9.52E+02	1.78E+03	1.37E+03	8.27E+02	8.46E+02	6.11E+03	3.11E+03
F23	Mean	5.21E+03	3.69E+03	7.33E+03	3.99E+03	5.30E+03	5.54E+03	4.14E+03	3.91E+03	**3.53E+03**
	Std	1.15E+02	1.04E+02	5.37E+02	1.60E+02	3.44E+02	2.09E+02	2.17E+02	1.17E+02	1.03E+02
F24	Mean	7.38E+03	4.74E+03	1.17E+04	4.85E+03	6.97E+03	7.30E+03	4.73E+03	4.66E+03	**4.00E+03**
	Std	2.80E+02	1.78E+02	1.09E+03	3.38E+02	3.18E+02	3.64E+02	2.37E+02	1.50E+02	8.97E+01
F25	Mean	2.23E+04	3.98E+03	2.90E+04	4.52E+03	1.11E+04	2.11E+04	4.02E+03	4.57E+03	**3.72E+03**
	Std	2.57E+03	8.51E+01	3.46E+03	4.59E+02	1.67E+03	2.04E+03	9.94E+01	2.68E+02	4.13E+01
F26	Mean	4.17E+04	2.88E+04	5.32E+04	2.58E+04	3.18E+04	3.83E+04	2.38E+04	2.15E+04	**1.86E+04**
	Std	3.25E+03	2.18E+03	3.35E+03	3.67E+03	1.89E+03	4.22E+03	3.47E+03	3.09E+03	1.42E+03
F27	Mean	8.57E+03	4.72E+03	1.35E+04	4.20E+03	6.68E+03	6.27E+03	4.70E+03	4.17E+03	**4.13E+03**
	Std	8.05E+02	3.35E+02	1.30E+03	3.46E+02	7.23E+02	6.72E+02	2.81E+02	1.67E+02	9.81E+01
F28	Mean	2.75E+04	4.64E+03	3.44E+04	5.49E+03	1.55E+04	2.33E+04	4.23E+03	5.20E+03	**3.93E+03**
	Std	3.08E+03	2.79E+02	3.29E+03	8.25E+02	2.02E+03	1.59E+03	1.38E+02	2.88E+02	5.34E+01
F29	Mean	3.13E+04	1.09E+04	7.13E+05	8.62E+03	2.28E+04	2.92E+04	9.45E+03	**8.00E+03**	9.08E+03
	Std	9.49E+03	9.75E+02	6.38E+05	6.55E+02	1.95E+04	1.60E+04	8.40E+02	6.10E+02	4.64E+02
F30	Mean	1.32E+10	4.76E+07	4.00E+10	**3.70E+06**	9.01E+09	8.56E+09	1.52E+07	7.22E+06	9.26E+07
	Std	2.87E+09	2.38E+07	7.47E+09	3.12E+06	3.82E+09	5.43E+09	7.95E+06	3.46E+06	2.24E+07
Mean rank		7.9	3.17	8.47	3.27	6.37	7	4.2	3.1	**1.53**
Final ranking		8	3	9	4	6	7	5	2	1
+/=/−		29/0/0	26/0/3	29/0/0	23/0/6	29/0/0	29/0/0	27/0/2	24/0/5	

+ indicates that SPO obtains better results than others, = indicates that SPO obtains equal results than others, − indicates that SPO is worse than others.

**Table 9:** Comparison of results between MBAs and SPO on CEC2019.

F	Type	SCA	RUN	AOA	INFO	SCHO	EDO	TTAO	QIO	SPO
F1	Mean	5.01E+06	1.00E+00	3.13E+06	**1.00E+00**	**1.00E+00**	**1.00E+00**	3.53E+05	1.00E+00	4.51E+03
	Std	9.03E+06	7.79E−14	1.07E+07	0.00E+00	0.00E+00	0.00E+00	4.37E+05	1.50E−08	4.01E+03
F2	Mean	4.56E+03	**4.30E+00**	1.09E+04	4.35E+00	5.00E+00	4.97E+00	4.63E+02	4.33E+00	8.98E+01
	Std	1.62E+03	1.75E−01	3.86E+03	1.44E−01	1.35E−03	1.27E−01	4.91E+02	1.26E−01	3.44E+01
F3	Mean	9.45E+00	1.61E+00	1.03E+01	2.18E+00	6.89E+00	6.29E+00	3.75E+00	2.82E+00	**1.43E+00**
	Std	1.34E+00	5.30E−01	1.15E+00	2.17E+00	2.72E+00	8.54E−01	1.65E+00	8.03E−01	2.16E−01
F4	Mean	5.08E+01	3.14E+01	6.12E+01	2.63E+01	4.76E+01	4.67E+01	2.43E+01	1.72E+01	**1.00E+01**
	Std	8.36E+00	1.05E+01	1.39E+01	9.31E+00	1.79E+01	6.95E+00	8.39E+00	6.85E+00	2.90E+00
F5	Mean	9.40E+00	1.39E+00	8.04E+01	1.14E+00	1.15E+01	2.28E+00	1.14E+00	1.08E+00	**1.00E+00**
	Std	2.67E+00	3.15E−01	2.53E+01	7.88E−02	1.58E+01	1.97E−01	1.03E−01	4.97E−02	1.37E−03
F6	Mean	7.72E+00	6.30E+00	1.09E+01	3.61E+00	6.37E+00	7.03E+00	3.59E+00	2.51E+00	**1.12E+00**
	Std	1.09E+00	1.31E+00	1.23E+00	1.43E+00	1.71E+00	1.27E+00	1.23E+00	1.07E+00	7.34E−02
F7	Mean	1.62E+03	6.90E+02	1.42E+03	8.57E+02	1.16E+03	1.49E+03	9.64E+02	7.96E+02	**6.33E+02**
	Std	2.63E+02	2.79E+02	2.88E+02	3.06E+02	3.76E+02	1.75E+02	1.87E+02	3.61E+02	1.64E+02
F8	Mean	4.57E+00	**3.73E+00**	4.75E+00	4.01E+00	4.68E+00	4.69E+00	4.04E+00	3.86E+00	3.74E+00
	Std	2.29E−01	5.03E−01	4.18E−01	5.03E−01	3.37E−01	1.67E−01	2.84E−01	4.56E−01	2.65E−01
F9	Mean	1.65E+00	1.35E+00	3.06E+00	1.19E+00	1.52E+00	1.44E+00	1.29E+00	1.23E+00	**1.14E+00**
	Std	1.75E−01	5.59E−02	6.99E−01	8.61E−02	4.51E−01	9.28E−02	1.00E−01	7.02E−02	2.61E−02
F10	Mean	2.15E+01	2.10E+01	2.11E+01	2.11E+01	2.15E+01	2.14E+01	2.04E+01	1.96E+01	**1.70E+01**
	Std	9.74E−02	2.40E+00	8.94E−02	9.88E−02	1.02E−01	2.29E−01	4.05E+00	5.76E+00	8.06E+00
Mean rank		8	3.4	8.3	3.5	6.2	5.9	4.5	2.8	**2.1**
Final ranking		8	3	9	4	7	6	5	2	1
+/=/−		10/0/0	7/0/3	10/0/0	8/0/2	8/0/2	8/0/2	10/0/0	8/0/2	

+ indicates that SPO obtains better results than others, = indicates that SPO obtains equal results than others, − indicates that SPO is worse than others.

**Table 10:** Comparison of results between MBAs and SPO on CEC2020.

F	Type	SCA	RUN	AOA	INFO	SCHO	EDO	TTAO	QIO	SPO
F1	Mean	8.58E+09	3.34E+03	3.15E+10	1.55E+03	5.43E+09	5.52E+09	1.87E+05	9.39E+03	**3.11E+02**
	Std	1.93E+09	4.73E+03	6.30E+09	2.67E+03	3.88E+09	3.72E+09	3.83E+05	8.87E+03	3.16E+02
F2	Mean	5.42E+03	**2.41E+03**	4.95E+03	2.54E+03	3.64E+03	4.66E+03	3.17E+03	2.45E+03	2.60E+03
	Std	3.03E+02	4.16E+02	4.32E+02	4.00E+02	3.96E+02	3.24E+02	4.33E+02	7.22E+02	2.37E+02
F3	Mean	9.59E+02	8.58E+02	1.01E+03	8.14E+02	8.88E+02	9.35E+02	8.10E+02	7.88E+02	**7.40E+02**
	Std	2.26E+01	2.64E+01	2.75E+01	3.07E+01	4.10E+01	2.78E+01	2.48E+01	2.26E+01	5.13E+00
F4	Mean	4.65E+03	1.92E+03	3.61E+05	1.91E+03	1.41E+04	2.51E+03	1.91E+03	1.91E+03	**1.90E+03**
	Std	2.24E+03	1.14E+01	2.29E+05	9.25E+00	4.52E+04	1.33E+03	2.92E+00	3.08E+00	9.52E−01
F5	Mean	3.04E+06	5.03E+04	7.28E+06	5.95E+04	1.60E+06	**8.88E+03**	2.02E+05	1.00E+05	1.33E+04
	Std	1.86E+06	2.63E+04	4.37E+06	3.96E+04	1.44E+06	3.59E+03	1.16E+05	1.03E+05	9.92E+03
F6	Mean	2.50E+03	1.97E+03	3.09E+03	1.86E+03	2.24E+03	2.42E+03	1.92E+03	**1.84E+03**	1.94E+03
	Std	1.97E+02	2.29E+02	3.37E+02	1.71E+02	2.10E+02	1.50E+02	1.55E+02	1.24E+02	1.27E+02
F7	Mean	1.04E+06	3.96E+04	4.09E+06	2.23E+04	9.36E+05	**4.59E+03**	5.23E+04	3.57E+04	5.14E+03
	Std	6.12E+05	2.74E+04	4.89E+06	2.50E+04	1.02E+06	5.41E+02	4.61E+04	4.50E+04	1.63E+03
F8	Mean	5.42E+03	2.55E+03	6.03E+03	3.22E+03	4.72E+03	3.08E+03	2.63E+03	2.30E+03	**2.30E+03**
	Std	1.94E+03	7.83E+02	8.15E+02	1.33E+03	1.52E+03	3.75E+02	9.89E+02	8.81E−01	5.23E−02
F9	Mean	3.02E+03	2.86E+03	3.44E+03	2.88E+03	3.07E+03	3.02E+03	2.87E+03	2.88E+03	**2.83E+03**
	Std	2.81E+01	2.08E+01	1.52E+02	3.42E+01	8.17E+01	4.37E+01	6.45E+01	2.91E+01	1.14E+01
F10	Mean	3.29E+03	2.99E+03	5.49E+03	2.98E+03	3.19E+03	3.20E+03	2.98E+03	2.97E+03	**2.95E+03**
	Std	9.46E+01	3.16E+01	1.02E+03	3.13E+01	1.86E+02	1.70E+02	2.82E+01	2.65E+01	2.85E+01
Mean rank		7.9	3.7	8.9	3.6	6.7	5.4	4.2	2.8	**1.8**
Final ranking		8	4	9	3	7	6	5	2	1
+/=/−		10/0/0	9/0/1	10/0/0	8/0/2	10/0/0	8/0/2	9/0/1	8/0/2	

+ indicates that SPO obtains better results than others, = indicates that SPO obtains equal results than others, − indicates that SPO is worse than others.

**Table 11:** Comparison of results between MBAs and SPO on CEC2022 10 dimensional.

F	Type	SCA	RUN	AOA	INFO	SCHO	EDO	TTAO	QIO	SPO
F1	Mean	2.31E+03	3.00E+02	1.33E+04	3.00E+02	5.11E+03	4.45E+02	3.00E+02	3.08E+02	**3.00E+02**
	Std	1.44E+03	1.08E−02	6.70E+03	3.86E−06	3.30E+03	8.19E+01	5.14E−01	1.29E+01	7.99E−08
F2	Mean	4.76E+02	4.08E+02	1.60E+03	4.10E+02	4.66E+02	4.17E+02	4.12E+02	4.05E+02	**4.00E+02**
	Std	1.52E+01	1.42E+01	7.41E+02	1.82E+01	8.06E+01	1.05E+01	2.49E+01	1.36E+01	1.06E−01
F3	Mean	6.22E+02	6.14E+02	6.40E+02	6.01E+02	6.22E+02	6.16E+02	6.04E+02	**6.00E+02**	6.01E+02
	Std	5.07E+00	7.28E+00	7.75E+00	1.64E+00	1.09E+01	6.34E+00	4.14E+00	1.20E+00	1.10E+00
F4	Mean	8.48E+02	8.23E+02	8.39E+02	8.20E+02	8.44E+02	8.35E+02	8.17E+02	8.13E+02	**8.05E+02**
	Std	7.69E+00	6.65E+00	9.90E+00	9.83E+00	1.63E+01	5.90E+00	7.17E+00	5.00E+00	1.56E+00
F5	Mean	1.05E+03	1.02E+03	1.39E+03	9.61E+02	1.48E+03	9.91E+02	9.01E+02	9.09E+02	**9.00E+02**
	Std	8.18E+01	7.99E+01	1.82E+02	6.73E+01	4.41E+02	7.55E+01	1.22E+00	1.45E+01	1.06E−04
F6	Mean	4.77E+06	3.41E+03	6.43E+07	2.02E+03	2.39E+04	2.10E+03	3.15E+03	**1.83E+03**	1.87E+03
	Std	4.64E+06	1.24E+03	2.05E+08	5.84E+02	2.67E+04	2.21E+02	1.64E+03	5.59E+01	1.74E+01
F7	Mean	2.06E+03	2.04E+03	2.11E+03	2.02E+03	2.08E+03	2.05E+03	2.03E+03	**2.02E+03**	2.03E+03
	Std	1.02E+01	1.13E+01	3.80E+01	9.79E+00	3.90E+01	7.57E+00	8.57E+00	1.08E+01	8.03E+00
F8	Mean	2.24E+03	2.22E+03	2.30E+03	2.22E+03	2.26E+03	2.23E+03	2.23E+03	2.22E+03	**2.22E+03**
	Std	3.86E+00	2.58E+00	1.15E+02	5.53E+00	5.44E+01	2.45E+00	5.29E+00	7.38E+00	4.94E+00
F9	Mean	2.58E+03	2.53E+03	2.75E+03	2.53E+03	2.61E+03	2.53E+03	**2.53E+03**	2.53E+03	2.53E+03
	Std	2.02E+01	2.08E+01	8.39E+01	2.08E+01	4.96E+01	1.56E+00	3.40E−07	3.42E−03	6.81E−04
F10	Mean	2.52E+03	2.54E+03	2.75E+03	2.57E+03	2.69E+03	2.51E+03	2.56E+03	2.51E+03	**2.51E+03**
	Std	5.15E+01	5.39E+01	2.30E+02	6.30E+01	2.51E+02	2.92E+01	1.32E+02	2.81E+01	2.52E+01
F11	Mean	3.01E+03	**2.69E+03**	3.39E+03	2.86E+03	3.30E+03	2.73E+03	2.90E+03	2.71E+03	2.90E+03
	Std	2.74E+02	1.36E+02	4.08E+02	1.24E+02	4.37E+02	2.28E+01	1.13E+02	1.30E+02	3.87E+01
F12	Mean	2.87E+03	2.86E+03	3.07E+03	2.86E+03	2.92E+03	2.87E+03	2.86E+03	2.87E+03	**2.86E+03**
	Std	2.11E+00	1.46E+00	8.18E+01	5.43E+00	4.65E+01	5.82E+00	1.20E+00	3.90E+00	1.37E+00
Mean rank		7.17	4.42	8.75	3.58	7.83	4.92	3.83	2.67	**1.83**
Final ranking		7	5	9	3	8	6	4	2	1
+/=/−		12/0/0	11/0/1	12/0/0	10/0/2	12/0/0	11/0/1	10/0/2	8/0/4	

+ indicates that SPO obtains better results than others, = indicates that SPO obtains equal results than others, − indicates that SPO is worse than others.

**Table 12:** Comparison of results between MBAs and SPO on CEC2022 20 dimensional.

F	Type	SCA	RUN	AOA	INFO	SCHO	EDO	TTAO	QIO	SPO
F1	Mean	2.13E+04	**3.06E+02**	3.51E+04	1.10E+03	2.27E+04	1.27E+04	1.57E+03	9.82E+03	3.06E+02
	Std	6.06E+03	5.82E+00	1.01E+04	8.55E+02	7.65E+03	4.92E+03	9.35E+02	3.49E+03	5.33E+00
F2	Mean	8.42E+02	4.57E+02	2.48E+03	**4.53E+02**	6.83E+02	7.07E+02	4.63E+02	4.68E+02	4.54E+02
	Std	1.16E+02	1.13E+01	6.31E+02	1.70E+01	1.86E+02	1.45E+02	1.66E+01	2.81E+01	9.80E+00
F3	Mean	6.51E+02	6.34E+02	6.66E+02	6.13E+02	6.52E+02	6.48E+02	6.26E+02	**6.07E+02**	6.08E+02
	Std	7.52E+00	9.89E+00	9.24E+00	8.21E+00	1.40E+01	1.05E+01	1.33E+01	5.21E+00	5.97E+00
F4	Mean	9.59E+02	8.79E+02	9.61E+02	8.67E+02	9.32E+02	9.43E+02	8.82E+02	8.48E+02	**8.27E+02**
	Std	1.30E+01	1.22E+01	1.62E+01	1.86E+01	2.83E+01	1.54E+01	1.84E+01	1.52E+01	7.00E+00
F5	Mean	2.77E+03	1.91E+03	3.07E+03	1.56E+03	3.59E+03	2.82E+03	1.89E+03	1.15E+03	**9.14E+02**
	Std	6.48E+02	2.81E+02	4.22E+02	3.26E+02	9.88E+02	5.90E+02	9.40E+02	2.05E+02	5.71E+01
F6	Mean	1.59E+08	3.63E+03	1.40E+09	5.61E+03	1.35E+07	1.43E+06	3.87E+03	3.92E+03	**2.08E+03**
	Std	1.12E+08	8.35E+02	1.27E+09	5.45E+03	2.48E+07	1.04E+06	2.22E+03	2.42E+03	3.58E+02
F7	Mean	2.17E+03	2.11E+03	2.26E+03	2.11E+03	2.20E+03	2.15E+03	2.11E+03	**2.05E+03**	2.08E+03
	Std	2.75E+01	3.03E+01	1.05E+02	4.64E+01	7.69E+01	2.68E+01	3.05E+01	1.53E+01	1.86E+01
F8	Mean	2.29E+03	2.24E+03	2.84E+03	2.28E+03	2.34E+03	2.25E+03	2.26E+03	2.23E+03	**2.23E+03**
	Std	3.89E+01	4.93E+01	9.78E+02	6.76E+01	9.97E+01	9.12E+00	4.72E+01	1.74E+01	1.13E+00
F9	Mean	2.62E+03	2.48E+03	3.25E+03	**2.48E+03**	2.60E+03	2.53E+03	2.48E+03	2.48E+03	2.48E+03
	Std	3.30E+01	5.41E−01	3.10E+02	4.45E−05	6.35E+01	2.56E+01	2.43E−01	3.02E+00	5.16E−02
F10	Mean	4.24E+03	2.69E+03	5.96E+03	3.25E+03	4.72E+03	3.68E+03	3.68E+03	**2.51E+03**	2.74E+03
	Std	1.91E+03	3.65E+02	9.85E+02	6.03E+02	6.62E+02	1.57E+03	9.46E+02	4.54E+01	4.99E+02
F11	Mean	6.96E+03	2.91E+03	1.08E+04	**2.90E+03**	5.23E+03	4.88E+03	2.91E+03	2.92E+03	2.91E+03
	Std	9.74E+02	3.26E+01	7.95E+03	4.47E−03	1.10E+03	6.41E+02	9.92E+00	1.24E+02	5.36E−01
F12	Mean	3.10E+03	2.98E+03	3.81E+03	2.98E+03	3.18E+03	3.01E+03	2.98E+03	2.99E+03	**2.96E+03**
	Std	3.73E+01	2.34E+01	2.40E+02	2.89E+01	9.93E+01	3.63E+01	3.28E+01	1.86E+01	1.28E+01
Mean rank		7.33	3	8.92	3.17	7.5	6	4.17	3.17	**1.75**
Final ranking		6	2	8	3	7	5	4	3	1
+/=/−		12/0/0	9/0/3	12/0/0	9/0/3	12/0/0	12/0/0	12/0/0	9/0/3	

+ indicates that SPO obtains better results than others, = indicates that SPO obtains equal results than others, − indicates that SPO is worse than others.

As seen in Tables 6, 7, 8, on the 30-dimensional CEC2017 test, the SPO algorithm obtained a mean rank of 1.77 and ranked first overall. The SPO algorithm achieves the best results on 63.3% of all functions. On the 50-dimensional CEC2017 test, the SPO algorithm ranked first with a mean of 1.47. Furthermore, it achieved the best results on 73.3% of all functions. On the 100-dimensional CEC2017 test, the SPO algorithm also ranked best with a mean of 1.53 and achieved the best results on 73.3% of all functions. The above results show that the SPO algorithm has a dominant performance in all dimensions of CEC2017 compared to the other eight MBAs and is more dominant in high dimensions.

As seen in Tables 9 and 10, On the CEC2019 and CEC2020, the SPO algorithm got the mean rankings of 2.1 and 1.8 separately. It was also ranked first on both test suits as well. Furthermore, SPO had the best search results on 70% of cec2019 and 60% of cec2020 functions.

As evidenced in Tables 11 and 12, The SPO algorithm ranked first on the 10-dimensional and the 20-dimensional CEC2022 tests with a mean rank of 1.83 and 1.75, respectively. The SPO algorithm achieved the best results on 58.3% and 41.7% of CEC2022. Similar to the test on CEC2017, SPO had a better mean rank on the higher dimensions.

Overall, the SPO algorithm achieves the best results in 65.7% of the functions tested compared to the other MBAs and performs better in higher dimensional tests.

#### Comparative analysis of SPO algorithm and OBAs algorithm

Tables 13, 14, 15, 16, 17, 18, 19 show the results of the SPO algorithm and other MBAs in seven types of tests. The best result has been marked in bold.

**Table 13:** Comparison of results between OBAs and SPO on CEC2017 with 30 dimensional.

F	Type	PSO	DO	SO	FLA	GOA	HEOA	KOA	YDSE	GKSO	SPO
F1	Mean	2.36E+09	9.19E+05	9.95E+06	1.94E+07	4.19E+10	1.83E+10	9.61E+10	1.40E+09	1.43E+04	**1.97E+03**
	Std	2.86E+09	8.03E+05	8.86E+06	5.91E+06	7.14E+09	5.87E+09	1.58E+10	5.90E+08	1.03E+04	1.12E+03
F3	Mean	7.05E+04	3.48E+04	7.18E+04	5.94E+04	8.47E+04	7.48E+04	3.25E+06	3.31E+04	**6.85E+03**	7.58E+03
	Std	2.15E+04	1.02E+04	9.28E+03	1.04E+04	6.15E+03	5.82E+03	1.35E+07	9.37E+03	2.21E+03	2.99E+03
F4	Mean	6.36E+02	5.16E+02	5.61E+02	5.29E+02	1.03E+04	2.46E+03	3.31E+04	6.42E+02	**5.05E+02**	5.09E+02
	Std	1.84E+02	2.88E+01	3.95E+01	3.81E+01	2.53E+03	1.19E+03	7.97E+03	4.10E+01	2.51E+01	1.42E+01
F5	Mean	5.90E+02	6.86E+02	6.00E+02	6.33E+02	8.50E+02	8.74E+02	1.10E+03	7.15E+02	6.77E+02	**5.74E+02**
	Std	2.50E+01	4.78E+01	2.32E+01	3.34E+01	2.78E+01	3.49E+01	3.16E+01	2.08E+01	4.01E+01	1.19E+01
F6	Mean	6.07E+02	6.49E+02	6.17E+02	**6.04E+02**	6.74E+02	6.79E+02	7.22E+02	6.45E+02	6.41E+02	6.18E+02
	Std	4.08E+00	1.34E+01	5.85E+00	3.54E+00	6.09E+00	6.68E+00	9.77E+00	7.96E+00	8.50E+00	6.77E+00
F7	Mean	8.45E+02	1.02E+03	9.13E+02	8.98E+02	1.31E+03	1.40E+03	3.10E+03	1.02E+03	9.54E+02	**7.93E+02**
	Std	4.45E+01	7.43E+01	3.80E+01	3.38E+01	5.98E+01	6.17E+01	1.96E+02	3.28E+01	6.77E+01	1.27E+01
F8	Mean	8.90E+02	9.54E+02	8.94E+02	9.23E+02	1.08E+03	1.09E+03	1.34E+03	1.00E+03	9.50E+02	**8.61E+02**
	Std	2.73E+01	4.35E+01	2.24E+01	2.52E+01	2.51E+01	3.10E+01	4.13E+01	1.90E+01	2.40E+01	1.09E+01
F9	Mean	2.10E+03	5.58E+03	2.44E+03	4.62E+03	8.58E+03	9.05E+03	3.00E+04	4.98E+03	3.73E+03	**1.34E+03**
	Std	1.78E+03	1.93E+03	8.41E+02	2.07E+03	1.10E+03	1.24E+03	4.34E+03	1.36E+03	8.36E+02	2.66E+02
F10	Mean	4.80E+03	5.24E+03	**4.22E+03**	5.02E+03	7.77E+03	7.72E+03	1.02E+04	7.42E+03	4.85E+03	4.37E+03
	Std	8.06E+02	7.72E+02	6.94E+02	6.21E+02	5.99E+02	6.40E+02	4.19E+02	4.75E+02	6.77E+02	3.77E+02
F11	Mean	1.35E+03	1.25E+03	1.42E+03	1.81E+03	6.49E+03	5.28E+03	2.80E+04	1.38E+03	**1.24E+03**	1.24E+03
	Std	2.62E+02	5.13E+01	9.98E+01	6.38E+02	1.60E+03	1.83E+03	1.02E+04	3.69E+01	4.89E+01	3.06E+01
F12	Mean	2.52E+08	8.98E+06	4.57E+06	8.91E+06	9.34E+09	8.87E+08	1.91E+10	1.92E+07	**1.37E+06**	2.99E+06
	Std	4.99E+08	7.35E+06	4.43E+06	6.51E+06	2.15E+09	7.21E+08	4.41E+09	1.22E+07	1.43E+06	2.23E+06
F13	Mean	3.09E+07	1.49E+05	4.75E+04	4.70E+05	5.84E+09	5.17E+07	1.49E+10	1.18E+05	**1.78E+04**	2.04E+04
	Std	1.92E+08	1.93E+05	2.76E+04	5.92E+05	3.02E+09	1.14E+08	4.79E+09	5.04E+04	1.42E+04	5.09E+03
F14	Mean	1.32E+05	1.35E+05	8.96E+04	1.26E+06	3.17E+06	1.59E+06	1.97E+07	**1.59E+03**	5.92E+03	1.94E+03
	Std	4.60E+05	1.30E+05	1.78E+05	1.14E+06	4.37E+06	9.42E+05	1.40E+07	2.40E+01	5.49E+03	3.49E+02
F15	Mean	2.40E+04	4.85E+04	1.47E+04	8.57E+04	1.69E+08	3.27E+06	3.30E+09	**5.46E+03**	9.78E+03	8.18E+03
	Std	3.31E+04	3.76E+04	1.12E+04	5.84E+04	1.57E+08	3.83E+06	1.22E+09	1.68E+03	1.04E+04	2.00E+03
F16	Mean	2.54E+03	2.92E+03	2.56E+03	2.76E+03	4.88E+03	3.89E+03	6.73E+03	3.13E+03	2.60E+03	**2.53E+03**
	Std	3.69E+02	3.55E+02	2.69E+02	3.27E+02	6.60E+02	5.89E+02	7.71E+02	2.46E+02	2.85E+02	1.60E+02
F17	Mean	2.15E+03	2.27E+03	2.22E+03	2.30E+03	3.42E+03	2.64E+03	6.27E+03	2.24E+03	2.25E+03	**1.93E+03**
	Std	2.37E+02	2.08E+02	2.24E+02	2.83E+02	9.13E+02	3.94E+02	3.10E+03	1.27E+02	2.34E+02	8.09E+01
F18	Mean	2.03E+06	1.56E+06	8.09E+05	2.50E+06	1.60E+07	9.67E+06	2.43E+08	**1.40E+04**	1.48E+05	4.04E+04
	Std	4.02E+06	2.18E+06	6.82E+05	3.27E+06	1.38E+07	8.30E+06	1.37E+08	6.90E+03	1.16E+05	1.50E+04
F19	Mean	1.61E+06	1.72E+05	1.42E+04	7.83E+04	2.33E+08	9.16E+06	4.28E+09	**6.69E+03**	1.02E+04	6.15E+04
	Std	1.05E+07	1.40E+05	1.37E+04	5.78E+04	2.40E+08	8.08E+06	2.05E+09	3.97E+03	9.49E+03	7.94E+04
F20	Mean	2.42E+03	2.67E+03	2.50E+03	2.54E+03	2.82E+03	2.78E+03	3.58E+03	2.65E+03	2.50E+03	**2.37E+03**
	Std	2.14E+02	2.43E+02	1.47E+02	2.15E+02	1.94E+02	1.92E+02	1.60E+02	1.24E+02	1.76E+02	8.50E+01
F21	Mean	2.40E+03	2.47E+03	2.39E+03	2.44E+03	2.66E+03	2.62E+03	2.85E+03	2.49E+03	2.44E+03	**2.37E+03**
	Std	3.20E+01	4.97E+01	1.58E+01	4.42E+01	3.83E+01	4.95E+01	3.84E+01	1.81E+01	3.44E+01	1.06E+01
F22	Mean	4.44E+03	5.84E+03	4.06E+03	5.44E+03	8.52E+03	7.72E+03	1.15E+04	7.53E+03	2.80E+03	**2.30E+03**
	Std	1.76E+03	1.77E+03	1.74E+03	1.85E+03	8.96E+02	1.41E+03	5.31E+02	2.07E+03	1.40E+03	3.88E−01
F23	Mean	2.88E+03	2.91E+03	2.81E+03	2.81E+03	3.46E+03	3.19E+03	3.67E+03	2.90E+03	2.86E+03	**2.74E+03**
	Std	8.09E+01	7.16E+01	4.61E+01	3.61E+01	1.51E+02	1.05E+02	1.30E+02	2.51E+01	7.21E+01	2.33E+01
F24	Mean	3.08E+03	3.09E+03	2.96E+03	3.01E+03	3.83E+03	3.22E+03	4.01E+03	3.05E+03	3.01E+03	**2.89E+03**
	Std	8.70E+01	6.69E+01	3.25E+01	4.00E+01	1.89E+02	8.80E+01	1.70E+02	2.90E+01	5.40E+01	1.71E+01
F25	Mean	2.94E+03	2.91E+03	2.94E+03	2.92E+03	4.33E+03	3.33E+03	1.44E+04	3.01E+03	**2.91E+03**	2.94E+03
	Std	7.40E+01	1.79E+01	3.19E+01	2.92E+01	2.81E+02	1.78E+02	2.19E+03	2.76E+01	2.16E+01	1.04E+01
F26	Mean	5.17E+03	5.64E+03	5.59E+03	5.18E+03	9.93E+03	8.40E+03	1.38E+04	6.05E+03	5.72E+03	**3.92E+03**
	Std	6.59E+02	1.13E+03	5.11E+02	7.35E+02	6.52E+02	1.22E+03	1.12E+03	3.03E+02	1.24E+03	1.04E+03
F27	Mean	3.28E+03	3.29E+03	3.29E+03	**3.24E+03**	4.54E+03	3.53E+03	4.83E+03	3.29E+03	3.28E+03	3.25E+03
	Std	6.57E+01	4.37E+01	3.33E+01	1.67E+01	2.68E+02	1.93E+02	3.22E+02	2.31E+01	4.56E+01	1.42E+01
F28	Mean	3.41E+03	3.27E+03	3.37E+03	3.29E+03	6.20E+03	4.50E+03	1.05E+04	3.43E+03	**3.25E+03**	3.26E+03
	Std	1.75E+02	2.79E+01	5.58E+01	4.89E+01	6.43E+02	4.40E+02	1.32E+03	6.50E+01	2.56E+01	2.10E+01
F29	Mean	**3.81E+03**	4.16E+03	4.13E+03	3.91E+03	6.36E+03	5.54E+03	1.19E+04	4.33E+03	4.15E+03	3.92E+03
	Std	1.91E+02	2.43E+02	2.07E+02	2.12E+02	6.74E+02	5.02E+02	5.32E+03	1.81E+02	2.48E+02	1.26E+02
F30	Mean	5.08E+05	1.70E+06	3.66E+05	2.65E+05	8.46E+08	9.02E+07	2.20E+09	4.85E+05	**1.22E+05**	2.96E+05
	Std	1.53E+06	8.22E+05	1.08E+06	4.29E+05	6.57E+08	6.61E+07	8.76E+08	3.43E+05	2.05E+05	2.48E+05
Mean rank		4.27	5.33	3.83	4.33	8.83	8.17	10	5.4	3.03	**1.8**
Final ranking		4	6	3	5	9	8	10	7	2	1
+/=/−		27/0/2	28/0/1	26/0/3	24/0/5	29/0/0	29/0/0	29/0/0	25/0/4	20/0/9	

+ indicates that SPO obtains better results than others, = indicates that SPO obtains equal results than others, − indicates that SPO is worse than others.

**Table 14:** Comparison of results between OBAs and SPO on CEC2017 with 50 dimensional.

F	Type	PSO	DO	SO	FLA	GOA	HEOA	KOA	YDSE	GKSO	SPO
F1	Mean	7.48E+09	2.28E+07	6.93E+08	1.61E+08	9.67E+10	5.16E+10	2.20E+11	1.35E+10	2.31E+06	**5.38E+04**
	Std	5.57E+09	1.13E+07	2.76E+08	3.14E+07	8.93E+09	1.30E+10	2.25E+10	2.48E+09	1.39E+06	1.26E+04
F3	Mean	2.25E+05	1.66E+05	1.61E+05	1.86E+05	1.72E+05	1.86E+05	8.21E+07	1.13E+05	7.08E+04	**6.18E+04**
	Std	6.25E+04	4.09E+04	1.75E+04	2.53E+04	1.80E+04	1.77E+04	4.45E+08	1.93E+04	1.46E+04	1.11E+04
F4	Mean	1.15E+03	**6.15E+02**	8.39E+02	6.75E+02	2.94E+04	9.97E+03	8.46E+04	1.87E+03	6.59E+02	6.34E+02
	Std	5.06E+02	5.32E+01	1.02E+02	6.75E+01	5.60E+03	2.96E+03	1.41E+04	4.72E+02	5.55E+01	3.40E+01
F5	Mean	7.08E+02	8.36E+02	7.20E+02	8.09E+02	1.11E+03	1.15E+03	1.60E+03	9.67E+02	8.29E+02	**6.66E+02**
	Std	5.04E+01	5.71E+01	4.28E+01	4.94E+01	3.25E+01	4.45E+01	6.14E+01	3.24E+01	4.09E+01	1.91E+01
F6	Mean	6.19E+02	6.58E+02	6.31E+02	**6.19E+02**	6.91E+02	6.95E+02	7.42E+02	6.67E+02	6.53E+02	6.34E+02
	Std	8.17E+00	8.22E+00	6.45E+00	7.67E+00	5.55E+00	5.55E+00	7.43E+00	6.83E+00	6.89E+00	5.40E+00
F7	Mean	1.08E+03	1.40E+03	1.20E+03	1.20E+03	1.93E+03	2.01E+03	5.54E+03	1.48E+03	1.38E+03	**9.57E+02**
	Std	7.90E+01	1.29E+02	8.00E+01	4.96E+01	6.79E+01	6.55E+01	3.27E+02	6.63E+01	1.57E+02	3.06E+01
F8	Mean	1.00E+03	1.13E+03	1.02E+03	1.10E+03	1.41E+03	1.46E+03	1.88E+03	1.27E+03	1.12E+03	**9.63E+02**
	Std	4.35E+01	5.91E+01	3.42E+01	4.50E+01	3.52E+01	4.18E+01	5.60E+01	3.41E+01	3.94E+01	1.95E+01
F9	Mean	1.21E+04	1.73E+04	7.36E+03	2.09E+04	3.24E+04	3.12E+04	8.51E+04	2.24E+04	1.07E+04	**5.18E+03**
	Std	9.01E+03	4.22E+03	3.18E+03	7.94E+03	3.26E+03	3.78E+03	7.53E+03	4.54E+03	2.44E+03	1.03E+03
F10	Mean	8.07E+03	8.30E+03	1.00E+04	8.43E+03	1.40E+04	1.34E+04	1.72E+04	1.35E+04	7.93E+03	**7.89E+03**
	Std	9.86E+02	1.01E+03	2.54E+03	9.91E+02	6.29E+02	9.12E+02	4.56E+02	6.34E+02	9.47E+02	5.72E+02
F11	Mean	1.95E+03	1.45E+03	3.30E+03	2.37E+03	2.20E+04	1.10E+04	7.78E+04	3.10E+03	**1.39E+03**	1.49E+03
	Std	1.79E+03	8.83E+01	1.17E+03	9.65E+02	3.02E+03	3.34E+03	2.21E+04	6.68E+02	7.64E+01	6.71E+01
F12	Mean	2.60E+09	5.81E+07	7.25E+07	9.07E+07	6.37E+10	1.50E+10	1.08E+11	5.93E+08	**2.57E+07**	3.94E+07
	Std	3.54E+09	3.62E+07	5.47E+07	4.82E+07	1.10E+10	7.56E+09	1.76E+10	2.45E+08	1.96E+07	1.50E+07
F13	Mean	1.15E+09	2.60E+05	2.95E+05	4.02E+06	3.48E+10	9.41E+08	5.82E+10	1.23E+07	4.06E+04	**3.16E+04**
	Std	1.90E+09	4.59E+05	2.79E+05	3.76E+06	9.18E+09	1.18E+09	1.41E+10	5.70E+06	3.53E+04	7.59E+03
F14	Mean	1.04E+06	7.68E+05	6.66E+05	5.21E+06	7.61E+07	7.13E+06	1.86E+08	**6.06E+03**	7.35E+04	1.76E+04
	Std	1.24E+06	5.56E+05	5.20E+05	3.67E+06	5.19E+07	7.33E+06	9.11E+07	3.23E+03	6.79E+04	1.22E+04
F15	Mean	4.06E+07	5.64E+04	4.36E+04	6.87E+05	5.38E+09	5.12E+08	1.92E+10	3.15E+05	1.20E+04	**1.10E+04**
	Std	1.15E+08	3.45E+04	4.46E+04	5.34E+05	2.55E+09	7.18E+08	6.20E+09	1.67E+05	6.87E+03	3.76E+03
F16	Mean	3.43E+03	3.75E+03	3.45E+03	3.72E+03	7.68E+03	6.51E+03	1.17E+04	4.72E+03	3.59E+03	**2.97E+03**
	Std	4.37E+02	5.29E+02	4.13E+02	5.01E+02	8.43E+02	1.22E+03	1.45E+03	3.54E+02	4.25E+02	2.80E+02
F17	Mean	3.33E+03	3.38E+03	3.24E+03	3.22E+03	5.71E+03	4.60E+03	2.63E+05	3.73E+03	3.36E+03	**2.82E+03**
	Std	4.13E+02	3.21E+02	3.32E+02	3.94E+02	1.48E+03	7.36E+02	2.16E+05	2.30E+02	3.29E+02	2.15E+02
F18	Mean	5.67E+06	4.88E+06	3.84E+06	8.04E+06	1.05E+08	4.31E+07	5.76E+08	**2.13E+05**	5.22E+05	2.43E+05
	Std	5.00E+06	4.06E+06	3.37E+06	6.61E+06	5.18E+07	2.10E+07	2.34E+08	1.84E+05	3.70E+05	1.66E+05
F19	Mean	4.10E+06	4.15E+05	8.88E+04	1.95E+05	2.46E+09	7.33E+07	8.45E+09	3.52E+05	**2.07E+04**	8.34E+04
	Std	2.53E+07	2.49E+05	1.04E+05	1.34E+05	1.21E+09	7.51E+07	2.47E+09	2.36E+05	1.18E+04	5.35E+04
F20	Mean	3.11E+03	3.47E+03	3.26E+03	3.28E+03	3.86E+03	3.89E+03	5.12E+03	3.82E+03	3.21E+03	**2.88E+03**
	Std	3.51E+02	3.41E+02	4.20E+02	3.43E+02	2.39E+02	2.23E+02	2.43E+02	2.45E+02	2.67E+02	1.44E+02
F21	Mean	2.54E+03	2.65E+03	2.51E+03	2.62E+03	3.06E+03	2.99E+03	3.45E+03	2.76E+03	2.63E+03	**2.44E+03**
	Std	6.36E+01	5.90E+01	3.63E+01	5.87E+01	6.63E+01	6.72E+01	6.12E+01	3.10E+01	7.21E+01	1.94E+01
F22	Mean	9.98E+03	1.04E+04	1.21E+04	1.02E+04	1.61E+04	1.45E+04	1.87E+04	1.52E+04	9.57E+03	**8.26E+03**
	Std	1.12E+03	9.43E+02	2.37E+03	1.39E+03	8.13E+02	9.72E+02	5.48E+02	5.90E+02	1.35E+03	3.04E+03
F23	Mean	3.34E+03	3.26E+03	3.10E+03	3.11E+03	4.36E+03	3.79E+03	4.87E+03	3.29E+03	3.19E+03	**2.96E+03**
	Std	1.54E+02	8.43E+01	6.82E+01	6.44E+01	1.86E+02	1.66E+02	2.40E+02	4.60E+01	9.83E+01	4.10E+01
F24	Mean	3.56E+03	3.47E+03	3.22E+03	3.36E+03	5.01E+03	3.87E+03	5.27E+03	3.48E+03	3.37E+03	**3.09E+03**
	Std	1.84E+02	1.16E+02	6.67E+01	1.13E+02	2.78E+02	1.28E+02	2.48E+02	4.61E+01	1.20E+02	3.00E+01
F25	Mean	3.25E+03	3.15E+03	3.39E+03	3.15E+03	1.35E+04	7.13E+03	4.75E+04	4.24E+03	**3.14E+03**	3.14E+03
	Std	2.56E+02	3.20E+01	1.38E+02	3.74E+01	1.27E+03	1.03E+03	7.51E+03	3.35E+02	3.20E+01	2.19E+01
F26	Mean	**7.07E+03**	9.74E+03	8.00E+03	7.23E+03	1.57E+04	1.46E+04	2.70E+04	9.44E+03	8.31E+03	7.23E+03
	Std	1.61E+03	1.14E+03	7.77E+02	8.19E+02	6.24E+02	1.37E+03	2.68E+03	4.66E+02	2.70E+03	1.55E+03
F27	Mean	3.75E+03	3.85E+03	3.83E+03	**3.54E+03**	7.13E+03	4.51E+03	7.67E+03	3.88E+03	3.76E+03	3.62E+03
	Std	1.92E+02	1.81E+02	1.17E+02	1.23E+02	6.82E+02	4.79E+02	6.64E+02	1.01E+02	1.66E+02	7.48E+01
F28	Mean	4.60E+03	3.44E+03	4.26E+03	3.47E+03	1.13E+04	7.76E+03	2.13E+04	5.08E+03	3.44E+03	**3.43E+03**
	Std	1.22E+03	5.09E+01	4.80E+02	7.18E+01	1.03E+03	7.13E+02	1.95E+03	5.07E+02	5.13E+01	4.56E+01
F29	Mean	4.64E+03	5.20E+03	5.22E+03	**4.58E+03**	4.33E+04	9.25E+03	3.13E+05	6.05E+03	5.41E+03	4.94E+03
	Std	4.14E+02	4.06E+02	4.19E+02	4.37E+02	3.21E+04	1.58E+03	2.57E+05	3.26E+02	4.43E+02	2.90E+02
F30	Mean	1.43E+07	2.67E+07	1.67E+07	**5.50E+06**	4.41E+09	4.99E+08	1.21E+10	5.19E+07	1.87E+07	4.19E+07
	Std	6.17E+07	6.61E+06	6.78E+06	1.81E+06	1.87E+09	2.93E+08	3.62E+09	1.48E+07	7.56E+06	1.08E+07
Mean rank		4.5	4.7	4	3.97	8.73	8.07	10	6.13	3.2	**1.7**
Final ranking		5	6	4	3	9	8	10	7	2	1
+/=/−		25/0/4	26/0/3	27/0/2	24/0/5	29/0/0	29/0/0	29/0/0	27/0/2	24/0/5	

+ indicates that SPO obtains better results than others, = indicates that SPO obtains equal results than others, − indicates that SPO is worse than others.

**Table 15:** Comparison of results between OBAs and SPO on CEC2017 with 100 dimensional.

F	Type	PSO	DO	SO	FLA	GOA	HEOA	KOA	YDSE	GKSO	SPO
F1	Mean	3.85E+10	8.19E+08	1.50E+10	2.54E+09	2.49E+11	1.72E+11	5.52E+11	9.33E+10	6.33E+08	**8.40E+07**
	Std	1.90E+10	3.04E+08	3.90E+09	5.76E+08	1.22E+10	2.02E+10	4.24E+10	9.26E+09	1.77E+08	1.98E+07
F3	Mean	6.48E+05	5.87E+05	3.78E+05	3.90E+05	3.37E+05	3.53E+05	3.91E+09	2.93E+05	2.96E+05	**2.80E+05**
	Std	1.27E+05	1.05E+05	3.61E+04	6.39E+04	1.12E+04	1.09E+04	1.46E+10	3.11E+04	2.42E+04	2.69E+04
F4	Mean	4.74E+03	1.16E+03	2.68E+03	1.29E+03	8.27E+04	3.12E+04	2.45E+05	1.11E+04	1.21E+03	**9.85E+02**
	Std	3.12E+03	1.15E+02	5.80E+02	1.52E+02	8.84E+03	7.85E+03	3.31E+04	1.69E+03	1.33E+02	4.07E+01
F5	Mean	1.26E+03	1.42E+03	1.19E+03	1.45E+03	1.99E+03	1.97E+03	2.95E+03	1.77E+03	1.34E+03	**1.00E+03**
	Std	1.16E+02	1.18E+02	6.67E+01	1.00E+02	4.86E+01	6.93E+01	9.37E+01	5.53E+01	6.50E+01	6.11E+01
F6	Mean	**6.46E+02**	6.73E+02	6.49E+02	6.51E+02	7.01E+02	7.05E+02	7.56E+02	6.92E+02	6.66E+02	6.49E+02
	Std	8.16E+00	1.01E+01	4.92E+00	9.64E+00	4.21E+00	3.35E+00	5.53E+00	6.40E+00	5.18E+00	4.84E+00
F7	Mean	2.12E+03	2.85E+03	2.25E+03	2.47E+03	3.86E+03	3.98E+03	1.24E+04	3.23E+03	2.67E+03	**1.62E+03**
	Std	2.70E+02	2.31E+02	1.16E+02	1.42E+02	9.41E+01	8.88E+01	5.57E+02	1.68E+02	2.67E+02	1.06E+02
F8	Mean	1.57E+03	1.79E+03	1.52E+03	1.77E+03	2.46E+03	2.42E+03	3.39E+03	2.09E+03	1.74E+03	**1.33E+03**
	Std	1.11E+02	1.29E+02	5.61E+01	1.01E+02	5.10E+01	6.86E+01	1.08E+02	6.82E+01	8.40E+01	6.52E+01
F9	Mean	6.02E+04	5.07E+04	3.04E+04	6.81E+04	7.44E+04	6.60E+04	2.04E+05	7.17E+04	2.82E+04	**2.24E+04**
	Std	1.62E+04	1.25E+04	8.39E+03	1.25E+04	4.81E+03	5.12E+03	1.33E+04	8.16E+03	2.94E+03	2.64E+03
F10	Mean	2.14E+04	1.86E+04	3.13E+04	2.16E+04	3.10E+04	2.89E+04	3.54E+04	3.04E+04	**1.70E+04**	1.99E+04
	Std	3.71E+03	1.26E+03	1.55E+03	1.37E+03	1.19E+03	1.53E+03	6.74E+02	9.68E+02	1.32E+03	8.38E+02
F11	Mean	8.62E+04	3.03E+04	1.37E+05	8.30E+04	1.75E+05	1.98E+05	4.32E+06	7.86E+04	**1.17E+04**	1.31E+04
	Std	3.52E+04	8.59E+03	2.30E+04	2.42E+04	2.02E+04	3.02E+04	1.31E+07	1.32E+04	3.16E+03	2.86E+03
F12	Mean	1.33E+10	4.81E+08	1.53E+09	9.51E+08	1.70E+11	6.81E+10	3.11E+11	1.45E+10	**4.44E+08**	5.36E+08
	Std	8.96E+09	1.85E+08	5.64E+08	3.60E+08	1.95E+10	1.63E+10	2.66E+10	3.07E+09	2.35E+08	7.89E+07
F13	Mean	1.94E+09	1.18E+06	4.76E+06	1.70E+07	3.81E+10	1.27E+10	7.52E+10	8.23E+08	5.80E+04	**2.93E+04**
	Std	2.36E+09	2.63E+06	6.26E+06	1.59E+07	5.42E+09	5.84E+09	9.99E+09	3.55E+08	1.93E+04	4.08E+03
F14	Mean	5.82E+06	4.89E+06	7.66E+06	1.01E+07	3.23E+07	2.29E+07	4.79E+08	7.65E+05	6.65E+05	**5.20E+05**
	Std	3.89E+06	2.59E+06	4.10E+06	4.10E+06	1.74E+07	8.67E+06	1.91E+08	3.89E+05	3.65E+05	2.71E+05
F15	Mean	4.02E+08	1.44E+05	4.04E+05	3.16E+06	1.71E+10	4.46E+09	3.81E+10	5.61E+07	4.20E+04	**2.00E+04**
	Std	8.66E+08	1.37E+05	3.72E+05	4.20E+06	4.15E+09	3.27E+09	6.55E+09	2.51E+07	3.82E+04	3.64E+03
F16	Mean	6.50E+03	7.31E+03	6.89E+03	7.03E+03	1.99E+04	1.49E+04	3.29E+04	1.06E+04	6.62E+03	**6.46E+03**
	Std	7.62E+02	6.59E+02	1.33E+03	6.45E+02	1.97E+03	2.37E+03	3.90E+03	5.88E+02	8.10E+02	4.92E+02
F17	Mean	7.17E+03	5.66E+03	5.66E+03	6.14E+03	3.05E+06	1.61E+05	3.15E+07	7.56E+03	5.60E+03	**4.39E+03**
	Std	2.36E+03	6.24E+02	5.63E+02	6.71E+02	3.51E+06	2.32E+05	2.09E+07	4.29E+02	5.88E+02	3.55E+02
F18	Mean	9.61E+06	6.16E+06	1.06E+07	9.74E+06	9.62E+07	2.79E+07	9.15E+08	1.30E+06	1.29E+06	**7.01E+05**
	Std	4.34E+06	2.48E+06	5.08E+06	4.40E+06	6.43E+07	1.39E+07	3.10E+08	7.86E+05	7.79E+05	2.82E+05
F19	Mean	2.42E+08	2.52E+06	2.04E+06	2.87E+06	1.87E+10	3.81E+09	3.81E+10	6.38E+07	**3.53E+05**	8.26E+05
	Std	3.59E+08	1.37E+06	1.72E+06	2.75E+06	4.13E+09	2.57E+09	6.28E+09	2.56E+07	3.55E+05	1.12E+06
F20	Mean	5.89E+03	5.84E+03	7.30E+03	5.59E+03	7.40E+03	6.83E+03	9.28E+03	7.21E+03	5.42E+03	**4.78E+03**
	Std	9.82E+02	5.69E+02	3.41E+02	5.37E+02	4.42E+02	4.65E+02	3.28E+02	3.80E+02	4.84E+02	2.96E+02
F21	Mean	3.33E+03	3.43E+03	3.11E+03	3.35E+03	4.68E+03	4.23E+03	5.18E+03	3.64E+03	3.32E+03	**2.78E+03**
	Std	1.27E+02	1.47E+02	6.58E+01	1.37E+02	1.84E+02	1.86E+02	1.67E+02	6.80E+01	1.35E+02	5.89E+01
F22	Mean	2.42E+04	2.15E+04	3.23E+04	2.39E+04	3.35E+04	3.22E+04	3.80E+04	3.29E+04	**2.01E+04**	2.16E+04
	Std	3.67E+03	1.64E+03	2.43E+03	1.37E+03	1.02E+03	1.26E+03	8.18E+02	8.91E+02	1.49E+03	3.11E+03
F23	Mean	4.76E+03	3.99E+03	3.70E+03	3.71E+03	7.21E+03	5.54E+03	7.56E+03	4.41E+03	3.97E+03	**3.53E+03**
	Std	3.34E+02	1.58E+02	1.03E+02	8.61E+01	3.80E+02	3.13E+02	3.64E+02	8.47E+01	1.78E+02	1.03E+02
F24	Mean	6.15E+03	4.89E+03	4.76E+03	4.50E+03	1.18E+04	6.52E+03	1.27E+04	5.24E+03	4.84E+03	**4.00E+03**
	Std	5.41E+02	2.14E+02	1.78E+02	1.53E+02	8.32E+02	4.21E+02	9.07E+02	1.63E+02	2.89E+02	8.97E+01
F25	Mean	5.34E+03	3.85E+03	5.46E+03	4.05E+03	2.63E+04	1.73E+04	1.16E+05	1.01E+04	3.84E+03	**3.72E+03**
	Std	1.32E+03	8.15E+01	4.87E+02	1.17E+02	1.91E+03	2.53E+03	1.05E+04	1.06E+03	9.99E+01	4.13E+01
F26	Mean	2.07E+04	2.22E+04	1.95E+04	**1.79E+04**	4.78E+04	4.14E+04	8.13E+04	2.34E+04	2.38E+04	1.86E+04
	Std	4.25E+03	1.82E+03	1.58E+03	1.51E+03	2.07E+03	2.50E+03	5.48E+03	1.15E+03	4.24E+03	1.42E+03
F27	Mean	4.29E+03	4.23E+03	4.38E+03	**3.88E+03**	1.36E+04	6.51E+03	1.51E+04	4.91E+03	4.25E+03	4.13E+03
	Std	4.41E+02	2.84E+02	2.22E+02	1.51E+02	1.06E+03	1.05E+03	1.32E+03	2.64E+02	2.48E+02	9.81E+01
F28	Mean	8.92E+03	4.01E+03	9.98E+03	5.37E+03	2.80E+04	1.93E+04	6.65E+04	1.39E+04	4.03E+03	**3.93E+03**
	Std	3.00E+03	1.60E+02	1.57E+03	3.42E+03	1.66E+03	1.40E+03	4.92E+03	1.66E+03	1.66E+02	5.34E+01
F29	Mean	**7.89E+03**	8.99E+03	9.44E+03	8.78E+03	2.54E+05	3.45E+04	5.83E+06	1.24E+04	9.54E+03	9.08E+03
	Std	7.57E+02	6.51E+02	8.79E+02	6.99E+02	1.50E+05	2.61E+04	3.22E+06	7.52E+02	9.75E+02	4.64E+02
F30	Mean	1.12E+09	4.50E+07	2.11E+07	**1.95E+07**	3.35E+10	9.33E+09	5.99E+10	5.22E+08	2.37E+07	9.26E+07
	Std	1.52E+09	1.97E+07	1.48E+07	6.80E+06	5.35E+09	4.29E+09	1.12E+10	1.77E+08	1.04E+07	2.24E+07
Mean rank		5.07	4	4.5	4.23	8.7	7.73	10	6.27	2.87	**1.63**
Final ranking		6	3	5	4	9	8	10	7	2	1
+/=/−		27/0/2	24/0/5	27/0/2	25/0/4	29/0/0	29/0/0	29/0/0	29/0/0	23/0/6	

+ indicates that SPO obtains better results than others, = indicates that SPO obtains equal results than others, − indicates that SPO is worse than others.

**Table 16:** Comparison of results between OBAs and SPO on CEC2019.

F	Type	PSO	DO	SO	FLA	GOA	HEOA	KOA	YDSE	GKSO	SPO
F1	Mean	3.59E+06	1.27E+05	4.17E+04	**1.00E+00**	**1.00E+00**	**1.00E+00**	6.25E+08	2.56E+03	1.00E+00	4.51E+03
	Std	1.39E+07	1.74E+05	5.66E+04	0.00E+00	0.00E+00	0.00E+00	3.05E+08	1.38E+03	1.95E−15	4.01E+03
F2	Mean	6.71E+02	8.31E+02	2.13E+02	1.93E+02	4.98E+00	4.84E+00	2.11E+04	1.57E+02	**4.48E+00**	8.98E+01
	Std	1.03E+03	5.13E+02	1.67E+02	2.70E+02	5.56E−02	1.91E−01	4.98E+03	4.26E+01	3.38E−01	3.44E+01
F3	Mean	3.56E+00	6.32E+00	4.81E+00	4.06E+00	4.01E+00	3.30E+00	1.20E+01	8.95E+00	1.75E+00	**1.43E+00**
	Std	2.50E+00	2.94E+00	1.81E+00	2.26E+00	9.97E−01	1.40E+00	3.63E−01	9.01E−01	1.01E+00	2.16E−01
F4	Mean	1.26E+01	2.99E+01	1.84E+01	1.78E+01	5.97E+01	6.32E+01	1.20E+02	2.40E+01	2.52E+01	**1.00E+01**
	Std	4.67E+00	1.14E+01	5.75E+00	7.56E+00	1.51E+01	1.66E+01	1.71E+01	6.21E+00	9.74E+00	2.90E+00
F5	Mean	1.67E+00	1.26E+00	1.16E+00	1.46E+00	5.38E+01	5.06E+00	1.27E+02	1.64E+00	1.20E+00	**1.00E+00**
	Std	2.12E+00	1.29E−01	1.52E−01	1.10E−01	2.00E+01	2.05E+00	3.18E+01	9.52E−02	9.66E−02	1.37E−03
F6	Mean	1.97E+00	5.28E+00	4.64E+00	4.21E+00	8.62E+00	9.32E+00	1.39E+01	6.37E+00	4.38E+00	**1.12E+00**
	Std	1.22E+00	1.74E+00	1.20E+00	1.18E+00	1.06E+00	1.40E+00	7.40E−01	8.69E−01	1.51E+00	7.34E−02
F7	Mean	7.63E+02	9.82E+02	6.73E+02	6.92E+02	1.46E+03	1.24E+03	2.43E+03	1.01E+03	8.60E+02	**6.33E+02**
	Std	2.01E+02	3.08E+02	1.91E+02	2.08E+02	3.38E+02	3.33E+02	2.25E+02	2.01E+02	2.88E+02	1.64E+02
F8	Mean	3.92E+00	4.35E+00	4.00E+00	3.98E+00	4.59E+00	4.72E+00	5.27E+00	4.50E+00	3.90E+00	**3.74E+00**
	Std	4.98E−01	4.01E−01	2.83E−01	4.29E−01	3.49E−01	2.79E−01	2.02E−01	2.01E−01	3.47E−01	2.65E−01
F9	Mean	1.18E+00	1.29E+00	1.35E+00	1.40E+00	2.83E+00	1.33E+00	4.91E+00	1.31E+00	1.20E+00	**1.14E+00**
	Std	9.06E−02	1.21E−01	1.24E−01	1.17E−01	5.81E−01	1.27E−01	8.19E−01	7.26E−02	7.80E−02	2.61E−02
F10	Mean	2.10E+01	2.10E+01	2.15E+01	2.11E+01	2.12E+01	2.12E+01	2.19E+01	2.14E+01	2.08E+01	**1.70E+01**
	Std	2.85E+00	4.85E−02	1.02E−01	3.32E−02	2.87E−01	9.68E−02	1.39E−01	1.05E−01	2.57E+00	8.06E+00
Mean rank		4.4	6.2	5.5	4.4	6.6	6.2	10	6.4	3.2	**1.8**
Final ranking		3	5	4	3	7	5	8	6	2	1
+/=/−		10/0/0	10/0/0	10/0/0	9/0/1	8/0/2	8/0/2	10/0/0	9/0/1	8/0/2	

+ indicates that SPO obtains better results than others, = indicates that SPO obtains equal results than others, − indicates that SPO is worse than others.

**Table 17:** Comparison of results between OBAs and SPO on CEC2020.

F	Type	PSO	DO	SO	FLA	GOA	HEOA	KOA	YDSE	GKSO	SPO
F1	Mean	6.82E+08	1.45E+04	1.11E+05	3.06E+06	2.20E+10	6.17E+09	4.70E+10	6.96E+07	2.30E+03	**3.11E+02**
	Std	1.18E+09	8.53E+03	1.36E+05	1.35E+06	4.34E+09	3.50E+09	7.01E+09	3.56E+07	2.72E+03	3.16E+02
F2	Mean	2.25E+03	2.64E+03	2.15E+03	**1.93E+03**	4.56E+03	4.35E+03	6.60E+03	4.14E+03	2.96E+03	2.60E+03
	Std	3.74E+02	5.14E+02	2.83E+02	2.61E+02	4.42E+02	4.22E+02	2.79E+02	3.24E+02	5.80E+02	2.37E+02
F3	Mean	7.57E+02	8.20E+02	8.03E+02	7.53E+02	9.54E+02	1.00E+03	1.94E+03	8.45E+02	8.27E+02	**7.40E+02**
	Std	1.18E+01	2.76E+01	2.55E+01	1.04E+01	3.08E+01	3.45E+01	1.58E+02	2.04E+01	3.84E+01	5.13E+00
F4	Mean	2.70E+03	1.91E+03	1.91E+03	1.91E+03	2.07E+05	3.75E+03	2.97E+06	1.91E+03	1.91E+03	**1.90E+03**
	Std	2.52E+03	4.17E+00	2.77E+00	2.11E+00	1.25E+05	2.65E+03	1.92E+06	2.70E+00	3.52E+00	9.52E−01
F5	Mean	5.16E+05	4.73E+05	4.22E+05	1.11E+06	3.21E+06	1.24E+06	4.50E+07	**3.72E+03**	3.35E+04	1.33E+04
	Std	4.65E+05	3.56E+05	3.44E+05	8.52E+05	2.28E+06	8.15E+05	2.53E+07	3.16E+02	3.09E+04	9.92E+03
F6	Mean	1.89E+03	1.87E+03	1.92E+03	**1.70E+03**	2.78E+03	2.64E+03	4.02E+03	2.09E+03	1.97E+03	1.94E+03
	Std	1.83E+02	1.38E+02	2.06E+02	1.03E+02	3.11E+02	3.20E+02	3.52E+02	1.40E+02	1.99E+02	1.27E+02
F7	Mean	1.95E+05	1.49E+05	1.18E+05	5.85E+05	2.16E+06	4.35E+05	1.97E+07	**3.22E+03**	6.96E+03	5.14E+03
	Std	1.61E+05	1.16E+05	1.19E+05	5.22E+05	2.04E+06	4.33E+05	1.14E+07	1.75E+02	3.25E+03	1.63E+03
F8	Mean	3.04E+03	3.93E+03	2.85E+03	3.53E+03	5.17E+03	3.83E+03	7.56E+03	3.83E+03	2.30E+03	**2.30E+03**
	Std	1.07E+03	1.41E+03	9.42E+02	1.38E+03	7.00E+02	1.11E+03	7.38E+02	1.61E+03	6.32E+00	5.23E−02
F9	Mean	2.92E+03	2.96E+03	2.88E+03	2.91E+03	3.39E+03	3.02E+03	3.49E+03	2.91E+03	2.90E+03	**2.83E+03**
	Std	7.44E+01	4.61E+01	3.02E+01	3.64E+01	1.20E+02	7.66E+01	1.24E+02	1.71E+01	3.58E+01	1.14E+01
F10	Mean	**2.94E+03**	2.95E+03	2.97E+03	2.95E+03	4.51E+03	3.45E+03	8.65E+03	2.95E+03	2.96E+03	2.95E+03
	Std	3.63E+01	3.53E+01	3.15E+01	3.76E+01	5.73E+02	3.97E+02	1.51E+03	2.26E+01	3.65E+01	2.85E+01
Mean rank		4.6	4.9	3.7	4.1	8.9	7.9	10	4.7	4.1	**2.1**
Final ranking		4	6	2	3	8	7	9	5	3	1
+/=/−		7/0/3	9/0/1	8/0/2	8/0/2	10/0/0	10/0/0	10/0/0	7/0/3	10/0/0	

+ indicates that SPO obtains better results than others, = indicates that SPO obtains equal results than others, − indicates that SPO is worse than others.

**Table 18:** Comparison of results between OBAs and SPO on CEC2022 with 10 dimensional.

F	Type	PSO	DO	SO	FLA	GOA	HEOA	KOA	YDSE	GKSO	SPO
F1	Mean	3.00E+02	3.00E+02	9.11E+02	2.09E+03	8.55E+03	4.97E+03	2.99E+04	3.03E+02	3.00E+02	**3.00E+02**
	Std	1.14E−01	3.92E−01	5.98E+02	1.53E+03	2.92E+03	1.89E+03	1.03E+04	2.33E+00	1.15E−04	7.99E−08
F2	Mean	4.32E+02	4.17E+02	4.10E+02	4.19E+02	1.10E+03	4.34E+02	1.67E+03	4.06E+02	4.06E+02	**4.00E+02**
	Std	5.73E+01	2.63E+01	1.77E+01	2.71E+01	3.29E+02	3.76E+01	5.49E+02	2.34E+00	1.00E+01	1.06E−01
F3	Mean	**6.00E+02**	6.10E+02	6.01E+02	6.00E+02	6.36E+02	6.47E+02	6.73E+02	6.07E+02	6.04E+02	6.01E+02
	Std	1.18E−01	9.39E+00	1.85E+00	2.12E−01	8.03E+00	1.35E+01	1.06E+01	2.55E+00	4.59E+00	1.10E+00
F4	Mean	8.13E+02	8.27E+02	8.14E+02	8.29E+02	8.37E+02	8.36E+02	9.05E+02	8.21E+02	8.20E+02	**8.05E+02**
	Std	5.27E+00	8.86E+00	4.89E+00	1.26E+01	1.01E+01	8.69E+00	1.21E+01	5.79E+00	7.52E+00	1.56E+00
F5	Mean	9.05E+02	1.10E+03	9.38E+02	1.07E+03	1.22E+03	1.45E+03	3.29E+03	9.36E+02	9.14E+02	**9.00E+02**
	Std	3.55E+01	2.31E+02	4.46E+01	1.83E+02	1.45E+02	1.70E+02	6.44E+02	2.45E+01	3.49E+01	1.06E−04
F6	Mean	5.55E+03	4.29E+03	3.38E+03	6.47E+03	4.92E+07	7.17E+03	4.01E+08	**1.83E+03**	3.01E+03	1.87E+03
	Std	2.43E+03	1.76E+03	1.58E+03	7.15E+03	7.09E+07	6.48E+03	2.77E+08	1.45E+01	1.46E+03	1.74E+01
F7	Mean	**2.02E+03**	2.03E+03	2.03E+03	2.02E+03	2.07E+03	2.11E+03	2.17E+03	2.04E+03	2.02E+03	2.03E+03
	Std	8.49E+00	1.68E+01	1.26E+01	6.32E+00	2.28E+01	3.21E+01	3.60E+01	6.62E+00	8.79E+00	8.03E+00
F8	Mean	2.23E+03	2.23E+03	2.22E+03	2.22E+03	2.25E+03	2.23E+03	2.36E+03	2.23E+03	**2.22E+03**	2.22E+03
	Std	3.74E+01	2.37E+00	5.21E+00	1.57E+01	3.89E+01	8.32E+00	8.30E+01	3.80E+00	7.67E+00	4.94E+00
F9	Mean	2.55E+03	2.54E+03	2.53E+03	2.54E+03	2.72E+03	2.67E+03	2.86E+03	2.53E+03	2.53E+03	**2.53E+03**
	Std	4.41E+01	3.52E+01	4.00E+00	2.22E+01	3.91E+01	3.57E+01	8.85E+01	9.35E−02	2.08E+01	6.81E−04
F10	Mean	2.57E+03	2.59E+03	2.53E+03	2.54E+03	2.63E+03	2.63E+03	2.74E+03	**2.50E+03**	2.52E+03	2.51E+03
	Std	9.13E+01	1.07E+02	6.15E+01	6.37E+01	8.62E+01	3.63E+01	2.18E+02	1.29E−01	4.00E+01	2.52E+01
F11	Mean	2.78E+03	2.84E+03	2.90E+03	2.86E+03	3.45E+03	3.13E+03	5.30E+04	2.88E+03	**2.71E+03**	2.90E+03
	Std	1.59E+02	1.85E+02	4.48E+01	1.52E+02	4.29E+02	2.68E+02	9.99E+03	1.17E+02	1.48E+02	3.87E+01
F12	Mean	2.88E+03	2.87E+03	2.87E+03	2.87E+03	3.01E+03	2.88E+03	3.06E+03	**2.86E+03**	2.87E+03	2.86E+03
	Std	1.99E+01	1.86E+01	7.59E+00	1.59E+01	6.26E+01	2.27E+01	6.83E+01	9.66E−01	1.68E+00	1.37E+00
Mean rank		4.25	5.58	4.33	4.92	8.67	8.33	10	3.75	2.92	**2.25**
Final ranking		4	7	5	6	9	8	10	3	2	1
+/=/−		9/0/3	11/0/1	11/0/1	9/0/3	12/0/0	12/0/0	12/0/0	8/0/4	9/0/3	

+ indicates that SPO obtains better results than others, = indicates that SPO obtains equal results than others, − indicates that SPO is worse than others.

**Table 19:** Comparison of results between OBAs and SPO on CEC2022 with 20 dimensional.

F	Type	PSO	DO	SO	FLA	GOA	HEOA	KOA	YDSE	GKSO	SPO
F1	Mean	6.93E+03	1.47E+03	1.93E+04	2.61E+04	3.13E+04	2.74E+04	1.78E+06	4.12E+03	**3.04E+02**	3.06E+02
	Std	5.12E+03	1.38E+03	5.03E+03	9.66E+03	7.87E+03	7.84E+03	6.73E+06	2.26E+03	5.21E+00	5.33E+00
F2	Mean	4.88E+02	4.61E+02	4.64E+02	4.71E+02	1.71E+03	7.82E+02	5.78E+03	4.75E+02	4.55E+02	**4.54E+02**
	Std	5.65E+01	2.32E+01	2.46E+01	3.96E+01	4.43E+02	1.40E+02	1.51E+03	1.16E+01	1.50E+01	9.80E+00
F3	Mean	6.03E+02	6.37E+02	6.10E+02	**6.01E+02**	6.64E+02	6.68E+02	7.08E+02	6.27E+02	6.24E+02	6.08E+02
	Std	3.54E+00	1.50E+01	6.36E+00	5.75E−01	7.72E+00	1.34E+01	1.29E+01	6.05E+00	1.07E+01	5.97E+00
F4	Mean	8.47E+02	8.93E+02	8.44E+02	8.92E+02	9.37E+02	9.38E+02	1.08E+03	8.96E+02	8.69E+02	**8.27E+02**
	Std	1.60E+01	2.83E+01	1.47E+01	2.89E+01	1.62E+01	1.96E+01	2.17E+01	1.34E+01	1.78E+01	7.00E+00
F5	Mean	9.83E+02	2.35E+03	1.32E+03	2.44E+03	2.94E+03	3.16E+03	1.06E+04	1.65E+03	1.59E+03	**9.14E+02**
	Std	1.52E+02	6.61E+02	2.48E+02	1.36E+03	3.64E+02	3.85E+02	1.74E+03	3.24E+02	3.37E+02	5.71E+01
F6	Mean	5.94E+05	5.20E+03	8.61E+03	7.56E+04	7.75E+08	2.99E+07	4.91E+09	1.35E+04	6.28E+03	**2.08E+03**
	Std	3.94E+06	4.58E+03	8.01E+03	5.20E+04	5.94E+08	2.96E+07	1.58E+09	5.75E+03	4.48E+03	3.58E+02
F7	Mean	**2.07E+03**	2.15E+03	2.09E+03	2.09E+03	2.17E+03	2.22E+03	2.40E+03	2.11E+03	2.09E+03	2.08E+03
	Std	4.84E+01	6.34E+01	3.42E+01	4.77E+01	5.29E+01	7.24E+01	7.45E+01	1.99E+01	2.38E+01	1.86E+01
F8	Mean	2.28E+03	2.27E+03	2.24E+03	2.29E+03	2.39E+03	2.25E+03	3.32E+03	2.24E+03	2.24E+03	**2.23E+03**
	Std	6.64E+01	5.47E+01	2.92E+01	6.89E+01	1.38E+02	2.24E+01	7.43E+02	4.07E+00	3.38E+01	1.13E+00
F9	Mean	2.51E+03	2.48E+03	2.48E+03	2.49E+03	3.08E+03	2.66E+03	3.47E+03	2.49E+03	**2.48E+03**	2.48E+03
	Std	3.18E+01	6.84E−01	1.06E+00	7.38E+00	2.11E+02	6.51E+01	2.77E+02	2.34E+00	6.56E−02	5.16E−02
F10	Mean	2.96E+03	3.41E+03	3.18E+03	2.78E+03	5.08E+03	4.98E+03	6.78E+03	3.99E+03	2.76E+03	**2.74E+03**
	Std	4.16E+02	5.08E+02	4.39E+02	2.18E+02	1.25E+03	9.87E+02	1.54E+03	1.41E+03	5.79E+02	4.99E+02
F11	Mean	**2.90E+03**	2.90E+03	3.07E+03	3.13E+03	7.87E+03	5.12E+03	1.67E+05	4.75E+03	2.92E+03	2.91E+03
	Std	3.88E−01	4.50E+01	1.29E+02	9.70E+01	9.36E+02	8.89E+02	2.26E+04	3.30E+02	9.74E+01	5.36E−01
F12	Mean	3.01E+03	3.01E+03	3.02E+03	2.96E+03	3.82E+03	3.07E+03	3.94E+03	2.97E+03	2.98E+03	**2.96E+03**
	Std	5.53E+01	6.31E+01	3.82E+01	1.39E+01	2.24E+02	1.06E+02	1.93E+02	1.07E+01	3.02E+01	1.28E+01
Mean rank		4.33	4.75	4.17	5	8.67	8.08	10	5.25	3.17	**1.58**
Final ranking		4	5	3	6	9	8	10	7	2	1
+/=/−		9/0/3	11/0/1	12/0/0	11/0/1	12/0/0	12/0/0	12/0/0	12/0/0	10/0/2	

+ indicates that SPO obtains better results than others, = indicates that SPO obtains equal results than others, − indicates that SPO is worse than others.

Tables 13, 14, 15 show that the SPO algorithm achieved first rank on all three dimensions of the CEC2017 test. Moreover, as the dimensionality increased from 30 to 100 dimensions, the mean rank of SPO decreased from 1.8 to 1.63, and the number of functions achieving the best position rose from 46.6% to 63.3%. From the test results on CEC2022 in Tables 17 and 18, again, as the dimensionality increases from 10 to 20, the mean rank of the SPO algorithm improves from 2.25 to 1.58, and the function that achieves the optimal position improves from 41.6 to 58.3%. Furthermore, the SPO algorithm is also ranked first in both sets of tests. These results demonstrate that the SPO algorithm has excellent advantages in high-dimensional testing.

Naturally, the SPO algorithm also achieved first place on the tests on CEC2019 and CEC2020. As shown in Tables 16 and 17, the mean rank of SPO on the two sets of tests is 1.8 and 2.1, respectively, and achieves the best results on 70% of the CEC2019 and 50% of the CEC2020 tests, respectively.

Overall, the SPO algorithm achieves the best results in 60% of the functions tested compared to the other OBAs.

### Convergence analysis

The convergence and distribution of the SPO algorithm in different test suites are demonstrated in Figs. 6, 7, 8, 9, 10, 11, 12, 13.

**Figure 6:**
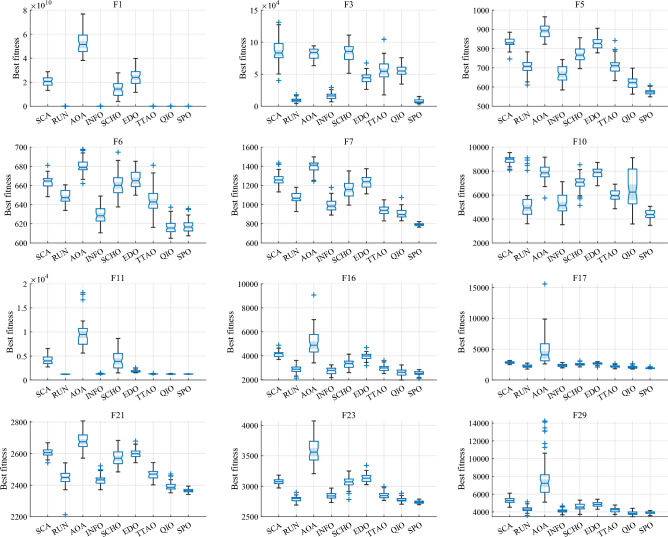
Boxplots of MBAs and SPO for solving 30-dimensional CEC2017 (portion).

**Figure 7:**
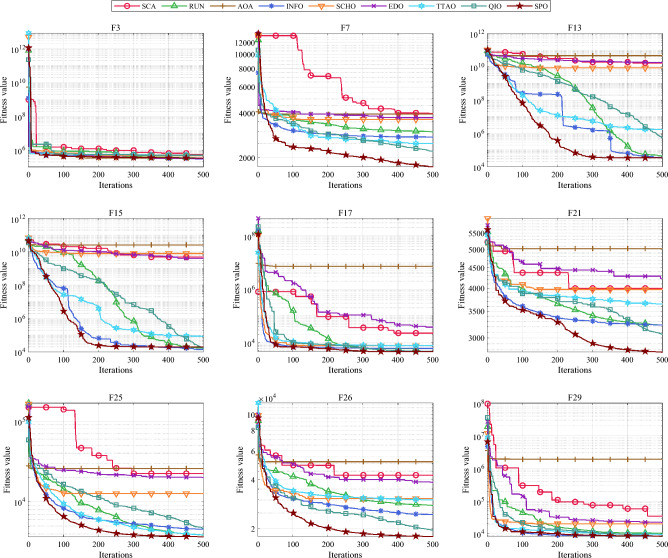
Convergence graphs of MBAs and SPO for solving 100-dimensional CEC2017 (portion).

**Figure 8:**
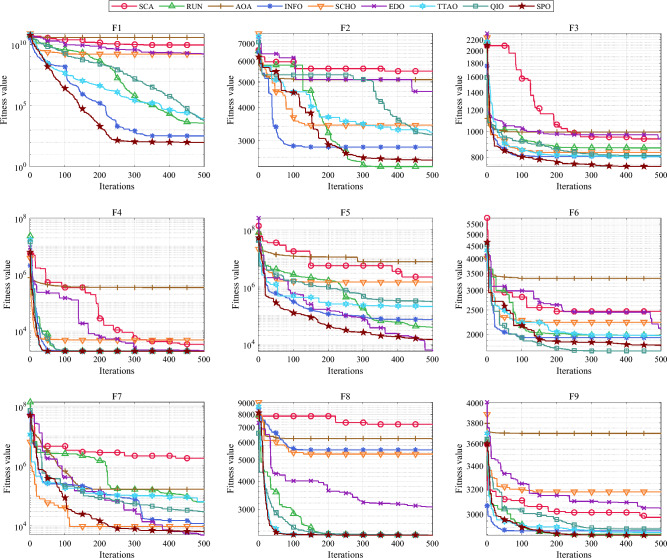
Convergence graphs of MBAs and SPO for solving 20-dimensional CEC2020 (portion).

**Figure 9:**
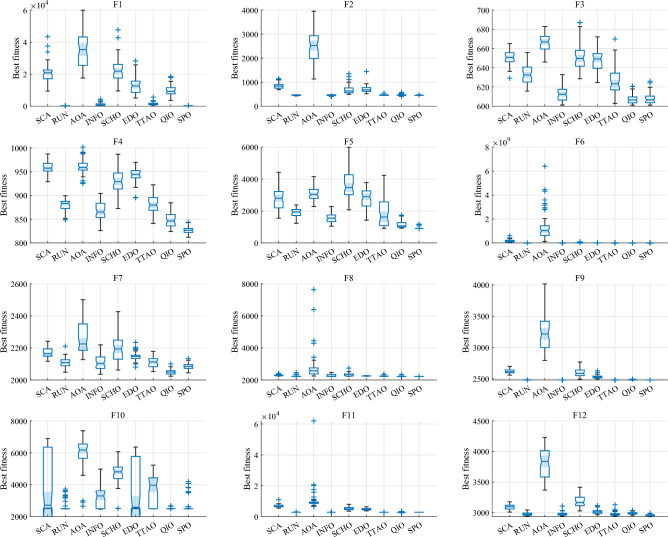
Boxplots of MBAs and SPO for solving 20-dimensional CEC2022.

**Figure 10:**
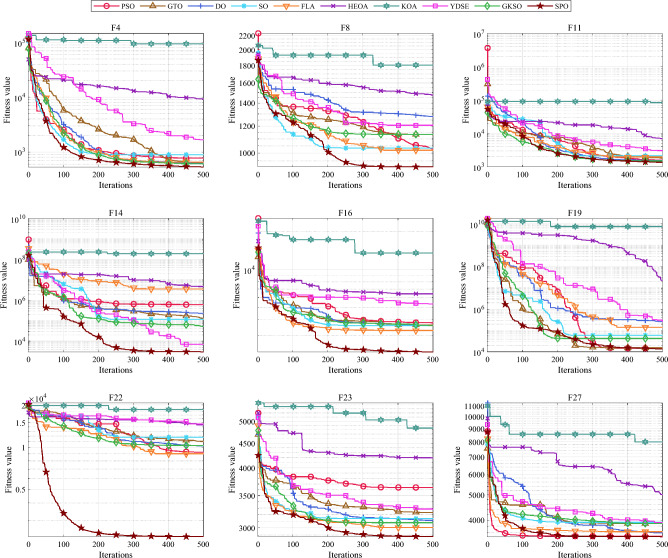
Convergence graphs of OBAs and SPO for solving 50-dimensional CEC2017 (portion).

**Figure 11:**
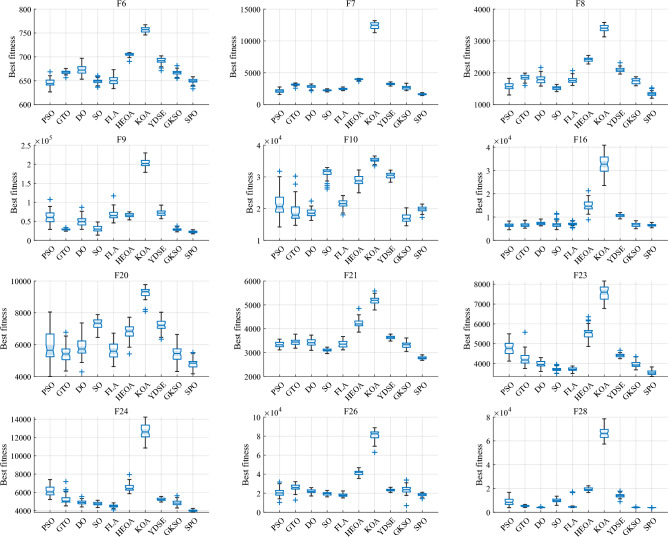
Boxplots of OBAs and SPO for solving 100-dimensional CEC2017 (portion).

**Figure 12:**
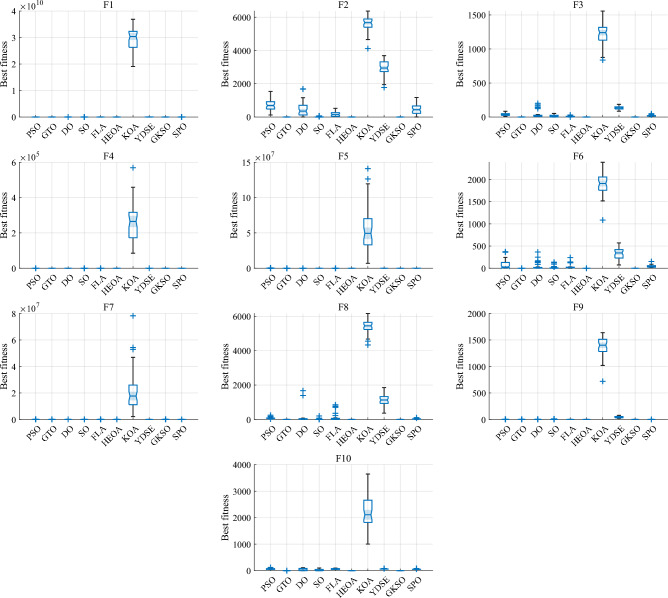
Boxplots of OBAs and SPO for solving 20-dimensional CEC2020.

**Figure 13:**
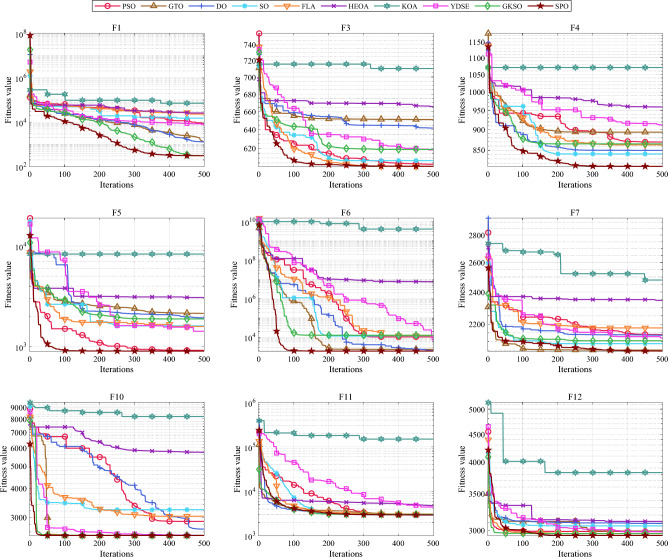
Convergence graphs of OBAs and SPO for solving 20-dimensional CEC2022 (portion).

From Fig. 7, 8, 10, and 13, it can be seen that the SPO algorithm can converge quickly in most of the test functions, including unimodal functions, multimodal functions, hybrid functions, and composition functions. The convergence curves in Figs. 8 and 13 show that on low-dimensional tests such as CEC2020 and CEC2022, SPO converges faster, essentially completing the global search within 200 rounds and being able to optimize the results locally in subsequent iterations. The convergence curves for the high-dimensional tests in Fig. 8 and 11 show that the SPO algorithm has a clear advantage with high-dimensional complex problems. F7, F13, F21, F25, and F26 in Fig. 8 and F8, F14, F16 and F22 in Fig. 11 show that SPO not only converges faster but also finds better results compared to other algorithms.

Figure 6, 9, 11 and 12 show the distribution of the results of each algorithm over 50 round tests. As can be seen from the figure, the SPO algorithm had a significantly smaller distribution compared to the other algorithms. This result indicates that SPO has higher stability than other algorithms and can better exclude the influence of random factors.

### Statistical analysis

#### Wilcoxon's rank sum test 

Tables 20, 21, 22, 23, 24, 25, 26, 27, 28, 29, 30, 31, 32, 33 demonstrate Wilcoxon's rank sum test results between the SPO algorithm versus the other algorithm at the 5% test level. A less than 0.05 indicates a significant difference between the two algorithms. Where the differences are not substantial, they are marked in bold.

**Table 20:** Wilcoxon's rank sum test results between MBAs and SPO on 30-dimensional CEC2017.

F	SCA	RUN	AOA	INFO	SCHO	EDO	TTAO	QIO
F1	7.07E−18	8.46E−18	7.07E−18	1.93E−11	7.07E−18	7.07E−18	7.07E−18	7.07E−18
F2	7.07E−18	**4.84E−01**	7.07E−18	7.56E−07	7.07E−18	7.07E−18	1.52E−07	7.07E−18
F3	7.07E−18	2.39E−03	7.07E−18	2.05E−13	7.07E−18	7.07E−18	7.07E−18	7.07E−18
F4	7.07E−18	6.14E−03	7.07E−18	**1.49E−01**	7.07E−18	7.07E−18	1.77E−07	7.55E−06
F5	7.07E−18	7.07E−18	7.07E−18	1.54E−17	7.07E−18	7.07E−18	7.07E−18	1.76E−13
F6	7.07E−18	8.46E−18	7.07E−18	1.96E−09	7.07E−18	7.07E−18	4.21E−15	**2.16E−01**
F7	7.07E−18	7.07E−18	7.07E−18	7.07E−18	7.07E−18	7.07E−18	7.07E−18	7.07E−18
F8	7.07E−18	7.97E−18	7.07E−18	3.58E−16	7.07E−18	7.07E−18	7.07E−18	3.38E−16
F9	7.07E−18	7.07E−18	7.07E−18	7.50E−18	7.07E−18	7.07E−18	2.07E−17	2.16E−13
F10	7.07E−18	3.37E−05	7.07E−18	9.14E−09	7.07E−18	7.07E−18	1.08E−17	2.81E−12
F11	7.07E−18	2.07E−05	7.07E−18	9.71E−04	7.07E−18	7.07E−18	9.44E−03	**8.01E−01**
F12	7.07E−18	3.69E−09	7.07E−18	2.22E−11	7.07E−18	7.07E−18	**5.06E−01**	**5.33E−01**
F13	7.07E−18	**1.55E−01**	7.07E−18	1.13E−02	7.07E−18	7.07E−18	4.60E−02	2.05E−09
F14	7.07E−18	7.07E−18	7.07E−18	3.10E−12	7.07E−18	2.32E−09	2.07E−17	1.03E−10
F15	7.07E−18	5.04E−16	7.07E−18	**7.59E−02**	7.07E−18	8.99E−18	1.95E−05	1.43E−05
F16	7.07E−18	3.54E−09	7.07E−18	1.70E−04	1.60E−16	7.07E−18	3.65E−14	**1.87E−01**
F17	7.07E−18	2.02E−11	7.07E−18	1.01E−16	7.97E−18	7.97E−18	1.29E−13	2.69E−04
F18	7.07E−18	2.02E−16	7.07E−18	8.68E−10	7.07E−18	1.12E−14	7.97E−18	4.76E−16
F19	7.07E−18	1.63E−06	7.07E−18	7.44E−12	3.97E−17	1.16E−13	3.46E−14	2.26E−14
F20	8.99E−18	3.16E−08	1.95E−17	1.34E−12	7.68E−15	1.01E−17	1.67E−13	**6.93E−02**
F21	7.07E−18	6.69E−16	7.07E−18	2.20E−17	7.07E−18	7.07E−18	7.07E−18	1.80E−09
F22	7.07E−18	1.26E−07	7.07E−18	2.75E−09	7.07E−18	7.07E−18	7.07E−18	7.07E−18
F23	7.07E−18	2.32E−09	7.07E−18	1.31E−15	8.46E−18	7.07E−18	3.13E−17	2.70E−08
F24	7.07E−18	6.72E−17	7.07E−18	1.84E−17	7.07E−18	7.07E−18	7.07E−18	3.79E−16
F25	7.07E−18	2.99E−04	7.07E−18	7.71E−08	7.07E−18	7.07E−18	**3.03E−01**	2.84E−04
F26	7.07E−18	1.28E−09	7.07E−18	1.32E−14	2.33E−17	3.97E−17	3.80E−13	5.90E−07
F27	7.07E−18	1.48E−12	7.07E−18	**9.19E−02**	7.07E−18	1.21E−17	8.32E−08	1.22E−05
F28	7.07E−18	2.23E−03	7.07E−18	2.73E−05	7.07E−18	7.07E−18	4.49E−08	1.98E−07
F29	7.07E−18	6.98E−13	7.07E−18	3.69E−07	1.91E−16	7.07E−18	3.12E−09	3.02E−02
F30	7.07E−18	1.32E−02	7.07E−18	8.46E−18	7.07E−18	7.07E−18	2.56E−15	1.54E−17

**Table 21:** Wilcoxon's rank sum test results between MBAs and SPO on 50-dimensional CEC2017.

F	SCA	RUN	AOA	INFO	SCHO	EDO	TTAO	QIO
F1	7.07E−18	7.07E−18	7.07E−18	7.07E−18	7.07E−18	7.07E−18	7.07E−18	7.07E−18
F2	7.07E−18	1.12E−14	7.07E−18	3.77E−15	7.07E−18	7.07E−18	1.69E−10	7.07E−18
F3	7.07E−18	**3.50E−01**	7.07E−18	1.11E−15	7.07E−18	7.50E−18	7.07E−18	7.07E−18
F4	7.07E−18	**7.64E−01**	7.07E−18	**3.23E−01**	7.07E−18	7.07E−18	4.76E−14	1.16E−13
F5	7.07E−18	7.07E−18	7.07E−18	7.97E−18	7.07E−18	7.07E−18	7.07E−18	3.53E−17
F6	7.07E−18	7.07E−18	7.07E−18	4.16E−08	7.07E−18	7.07E−18	6.72E−17	**9.42E−01**
F7	7.07E−18	7.07E−18	7.07E−18	7.07E−18	7.07E−18	7.07E−18	7.97E−18	9.54E−18
F8	7.07E−18	7.07E−18	7.07E−18	7.97E−18	7.07E−18	7.07E−18	7.07E−18	5.98E−17
F9	7.07E−18	7.07E−18	7.07E−18	5.02E−17	7.07E−18	7.07E−18	7.07E−18	4.50E−16
F10	7.07E−18	**8.60E−01**	7.07E−18	4.76E−03	7.07E−18	7.07E−18	7.07E−18	8.42E−09
F11	7.07E−18	1.50E−13	7.07E−18	6.27E−03	7.07E−18	7.07E−18	9.16E−06	6.01E−03
F12	7.07E−18	**8.77E−01**	7.07E−18	2.23E−09	7.07E−18	7.07E−18	**6.27E−01**	6.96E−03
F13	7.07E−18	**8.05E−02**	7.07E−18	3.02E−02	7.07E−18	7.07E−18	1.89E−12	1.58E−13
F14	7.07E−18	1.43E−13	7.07E−18	2.56E−11	7.07E−18	1.14E−17	7.07E−18	9.38E−16
F15	7.07E−18	6.32E−16	7.07E−18	**2.29E−01**	7.07E−18	7.07E−18	4.22E−04	1.77E−07
F16	7.07E−18	4.01E−09	7.07E−18	9.91E−09	7.07E−18	7.07E−18	2.42E−15	6.10E−05
F17	7.07E−18	6.88E−14	7.07E−18	4.71E−10	7.97E−18	7.07E−18	1.93E−10	4.68E−04
F18	7.07E−18	1.82E−14	7.07E−18	4.12E−07	7.07E−18	8.99E−17	3.58E−16	9.38E−16
F19	7.07E−18	6.59E−09	7.07E−18	5.15E−13	7.07E−18	7.07E−18	9.42E−14	2.17E−15
F20	7.07E−18	1.65E−04	7.50E−18	3.02E−15	1.73E−17	7.07E−18	1.76E−13	**1.23E−01**
F21	7.07E−18	7.07E−18	7.07E−18	7.07E−18	7.07E−18	7.07E−18	7.07E−18	1.43E−16
F22	7.07E−18	**3.98E−01**	7.07E−18	1.81E−03	7.07E−18	7.07E−18	7.55E−17	3.21E−03
F23	7.07E−18	7.97E−18	7.07E−18	1.73E−17	7.07E−18	7.07E−18	7.07E−18	8.11E−15
F24	7.07E−18	3.38E−16	7.07E−18	1.14E−17	7.07E−18	7.07E−18	7.07E−18	1.95E−17
F25	7.07E−18	1.18E−10	7.07E−18	1.04E−05	7.07E−18	7.07E−18	3.39E−11	2.56E−11
F26	7.07E−18	7.50E−18	7.07E−18	2.49E−08	7.07E−18	7.07E−18	4.18E−09	5.89E−04
F27	7.07E−18	1.55E−15	7.07E−18	1.37E−02	7.07E−18	1.84E−17	2.55E−12	7.58E−04
F28	7.07E−18	5.15E−13	7.07E−18	1.75E−06	7.07E−18	7.07E−18	2.81E−12	2.54E−16
F29	7.07E−18	5.14E−10	7.07E−18	8.71E−03	1.08E−17	7.07E−18	4.19E−06	2.27E−05
F30	7.07E−18	3.51E−04	7.07E−18	7.07E−18	7.07E−18	8.46E−18	7.07E−18	7.07E−18

**Table 22:** Wilcoxon's rank sum test results between MBAs and SPO on CEC2017 with 100 dimensional.

F	SCA	RUN	AOA	INFO	SCHO	EDO	TTAO	QIO
F1	7.07E−18	7.07E−18	7.07E−18	7.07E−18	7.07E−18	7.07E−18	7.07E−18	7.07E−18
F2	7.07E−18	2.85E−16	7.07E−18	8.99E−17	7.07E−18	7.07E−18	1.60E−16	1.08E−17
F3	7.07E−18	1.50E−03	1.73E−17	3.46E−10	7.50E−18	2.13E−08	7.07E−18	7.50E−18
F4	7.07E−18	7.07E−18	7.07E−18	7.07E−18	7.07E−18	7.07E−18	7.07E−18	7.07E−18
F5	7.07E−18	7.07E−18	7.07E−18	1.63E−17	7.07E−18	7.07E−18	7.07E−18	1.08E−17
F6	7.07E−18	7.07E−18	7.07E−18	4.69E−15	7.07E−18	7.07E−18	7.07E−18	2.56E−11
F7	7.07E−18	7.07E−18	7.07E−18	7.07E−18	7.07E−18	7.07E−18	7.50E−18	7.07E−18
F8	7.07E−18	7.07E−18	7.07E−18	8.46E−18	7.07E−18	7.07E−18	7.07E−18	7.50E−18
F9	7.07E−18	1.10E−12	7.07E−18	2.30E−08	7.07E−18	7.07E−18	7.07E−18	7.07E−18
F10	7.07E−18	1.49E−08	7.07E−18	1.13E−07	7.07E−18	7.07E−18	7.07E−18	9.02E−07
F11	7.07E−18	2.07E−02	7.07E−18	2.47E−17	7.07E−18	7.07E−18	7.07E−18	7.07E−18
F12	7.07E−18	**4.42E−01**	7.07E−18	3.46E−02	7.07E−18	7.07E−18	3.90E−02	1.01E−07
F13	7.07E−18	1.41E−10	7.07E−18	1.99E−12	7.07E−18	7.07E−18	7.07E−18	7.07E−18
F14	7.07E−18	2.42E−05	7.07E−18	1.19E−14	7.07E−18	8.99E−18	3.97E−17	3.74E−17
F15	7.07E−18	9.48E−04	7.07E−18	2.50E−03	7.07E−18	7.07E−18	9.53E−17	3.18E−05
F16	7.07E−18	1.12E−08	7.07E−18	**2.81E−01**	7.07E−18	7.07E−18	4.31E−10	**6.03E−02**
F17	7.07E−18	1.35E−16	7.07E−18	3.97E−17	7.07E−18	7.07E−18	8.86E−16	4.58E−12
F18	7.07E−18	3.69E−09	7.07E−18	2.29E−15	7.07E−18	7.07E−18	7.97E−18	7.50E−18
F19	7.07E−18	4.75E−02	7.07E−18	3.13E−17	7.07E−18	7.07E−18	1.44E−04	7.07E−18
F20	7.07E−18	2.22E−06	7.07E−18	6.98E−13	7.07E−18	7.07E−18	2.47E−17	1.47E−07
F21	7.07E−18	7.07E−18	7.07E−18	7.07E−18	7.07E−18	7.07E−18	7.07E−18	7.07E−18
F22	7.07E−18	**9.19E−02**	7.07E−18	**1.22E−01**	7.07E−18	7.07E−18	7.07E−18	1.17E−08
F23	7.07E−18	7.62E−10	7.07E−18	1.54E−17	7.07E−18	7.07E−18	1.01E−17	2.20E−17
F24	7.07E−18	7.07E−18	7.07E−18	7.07E−18	7.07E−18	7.07E−18	8.46E−18	7.07E−18
F25	7.07E−18	2.07E−17	7.07E−18	7.07E−18	7.07E−18	7.07E−18	8.99E−18	7.07E−18
F26	7.07E−18	6.34E−17	7.07E−18	4.25E−16	7.07E−18	7.07E−18	1.99E−12	7.14E−08
F27	7.07E−18	2.17E−15	7.07E−18	**9.48E−01**	7.07E−18	7.07E−18	1.80E−16	**2.96E−01**
F28	7.07E−18	7.07E−18	7.07E−18	7.07E−18	7.07E−18	7.07E−18	1.63E−17	7.07E−18
F29	7.07E−18	1.35E−16	7.07E−18	1.84E−04	7.07E−18	7.07E−18	2.67E−02	1.15E−12
F30	7.07E−18	3.43E−13	7.07E−18	7.07E−18	7.07E−18	7.07E−18	7.07E−18	7.07E−18

**Table 23:** Wilcoxon's rank sum test results between MBAs and SPO on CEC2019.

F	SCA	RUN	AOA	INFO	SCHO	EDO	TTAO	QIO
F1	1.39E−14	4.39E−19	**3.50E−01**	3.31E−20	3.31E−20	3.31E−20	2.33E−17	7.06E−18
F2	7.07E−18	7.07E−18	1.35E−16	7.07E−18	5.42E−19	1.23E−19	8.11E−15	7.07E−18
F3	7.07E−18	2.80E−03	7.07E−18	3.90E−04	1.84E−17	7.07E−18	1.51E−16	5.64E−17
F4	7.07E−18	6.32E−16	7.07E−18	1.24E−15	7.07E−18	7.07E−18	1.64E−15	1.52E−07
F5	7.07E−18	7.07E−18	7.07E−18	7.07E−18	7.07E−18	7.07E−18	1.21E−17	7.50E−18
F6	7.07E−18	7.07E−18	7.07E−18	1.95E−17	7.07E−18	7.07E−18	7.07E−18	1.05E−13
F7	8.99E−18	**2.63E−01**	1.70E−16	4.63E−06	8.59E−12	7.07E−18	1.41E−12	1.79E−02
F8	2.78E−17	**6.57E−01**	4.96E−15	9.45E−06	6.72E−17	1.08E−17	1.63E−06	2.48E−02
F9	7.07E−18	7.07E−18	8.46E−18	1.61E−03	7.55E−17	7.07E−18	4.76E−16	1.76E−11
F10	7.07E−18	1.01E−16	7.55E−17	1.14E−17	7.07E−18	7.55E−17	6.89E−15	4.90E−13

**Table 24:** Wilcoxon's rank sum test results between MBAs and SPO on CEC2020.

F	SCA	RUN	AOA	INFO	SCHO	EDO	TTAO	QIO
F1	7.07E−18	9.88E−10	7.07E−18	2.45E−06	7.07E−18	7.07E−18	7.07E−18	3.97E−17
F2	7.07E−18	1.02E−02	7.07E−18	**2.87E−01**	1.51E−16	7.07E−18	1.41E−10	4.46E−03
F3	7.07E−18	7.07E−18	7.07E−18	7.07E−18	7.07E−18	7.07E−18	7.07E−18	8.46E−18
F4	7.07E−18	1.21E−17	7.07E−18	3.19E−15	7.07E−18	7.07E−18	7.55E−17	1.43E−08
F5	7.07E−18	1.19E−14	7.07E−18	4.70E−11	7.07E−18	3.83E−02	1.37E−17	3.46E−14
F6	1.73E−17	**7.12E−01**	7.07E−18	3.66E−03	1.84E−11	1.21E−17	**2.37E−01**	4.00E−04
F7	7.07E−18	8.48E−17	7.07E−18	2.65E−10	7.50E−18	**2.93E−01**	1.73E−17	8.77E−09
F8	7.07E−18	7.56E−07	7.07E−18	1.52E−04	7.07E−18	7.07E−18	7.07E−18	2.20E−17
F9	7.07E−18	1.99E−12	7.07E−18	1.64E−15	7.07E−18	7.07E−18	7.27E−15	2.56E−15
F10	7.07E−18	3.56E−11	7.07E−18	4.69E−05	8.99E−18	9.54E−18	1.80E−09	1.07E−05

**Table 25:** Wilcoxon's rank sum test results between MBAs and SPO on CEC2022 with 10 dimensional.

F	SCA	RUN	AOA	INFO	SCHO	EDO	TTAO	QIO
F1	7.07E−18	7.07E−18	7.07E−18	3.18E−02	7.07E−18	7.07E−18	7.07E−18	7.07E−18
F2	7.07E−18	5.53E−15	7.07E−18	1.17E−16	7.97E−18	7.07E−18	5.04E−16	1.17E−15
F3	7.07E−18	9.54E−18	7.07E−18	**7.36E−02**	1.45E−17	7.07E−18	2.81E−06	2.20E−05
F4	7.07E−18	1.63E−17	7.07E−18	3.73E−17	7.07E−18	7.07E−18	3.53E−17	5.53E−15
F5	7.07E−18	7.07E−18	7.07E−18	7.07E−18	7.07E−18	7.07E−18	7.07E−18	3.46E−14
F6	7.07E−18	2.02E−16	7.07E−18	**8.23E−01**	7.07E−18	8.99E−18	4.51E−14	3.39E−11
F7	7.97E−18	3.59E−12	7.07E−18	**8.80E−02**	5.53E−15	1.84E−17	**9.31E−01**	8.89E−05
F8	7.07E−18	4.32E−08	8.99E−18	**2.63E−01**	4.21E−17	3.13E−17	2.02E−10	3.07E−04
F9	7.07E−18	4.38E−02	7.07E−18	1.49E−17	7.07E−18	7.07E−18	7.07E−18	**1.07E−01**
F10	1.12E−14	8.05E−14	1.43E−16	7.34E−13	5.29E−14	2.51E−14	1.84E−04	4.69E−05
F11	3.96E−02	3.98E−15	2.68E−12	4.17E−11	2.95E−11	1.20E−16	2.57E−05	1.60E−16
F12	7.07E−18	1.58E−07	7.07E−18	**1.18E−01**	7.07E−18	1.35E−02	3.32E−06	3.02E−16

**Table 26:** Wilcoxon's rank sum test results between MBAs and SPO on CEC2022 with 20 dimensional.

F	SCA	RUN	AOA	INFO	SCHO	EDO	TTAO	QIO
F1	7.07E−18	**2.18E−01**	7.07E−18	7.07E−18	7.07E−18	7.07E−18	7.07E−18	7.07E−18
F2	7.07E−18	5.28E−06	7.07E−18	4.97E−03	7.07E−18	7.07E−18	8.98E−08	4.55E−05
F3	7.07E−18	5.02E−17	7.07E−18	2.99E−04	7.07E−18	7.50E−18	3.61E−13	**6.47E−01**
F4	7.07E−18	7.07E−18	7.07E−18	4.21E−17	7.07E−18	7.07E−18	7.97E−18	8.97E−13
F5	7.07E−18	7.07E−18	7.07E−18	1.73E−17	7.07E−18	7.07E−18	8.48E−17	1.17E−15
F6	7.07E−18	4.44E−15	7.07E−18	1.65E−09	7.07E−18	7.07E−18	6.75E−12	1.65E−09
F7	9.54E−18	2.81E−06	7.50E−18	4.36E−03	6.53E−14	2.69E−16	1.22E−05	1.50E−13
F8	7.07E−18	2.23E−02	7.07E−18	**2.78E−01**	7.07E−18	7.07E−18	1.37E−17	3.07E−03
F9	7.07E−18	1.63E−12	7.07E−18	7.07E−18	7.07E−18	7.07E−18	4.52E−02	1.01E−17
F10	1.85E−10	1.18E−05	3.53E−17	1.11E−06	5.98E−17	2.42E−09	2.30E−08	6.14E−03
F11	7.07E−18	5.61E−10	7.07E−18	7.07E−18	7.07E−18	7.07E−18	2.96E−02	1.41E−12
F12	7.07E−18	3.55E−06	7.07E−18	5.64E−06	7.07E−18	1.94E−15	9.14E−05	2.05E−13

**Table 27:** Wilcoxon's rank sum test results between OBAs and SPO on CEC2017 with 30 dimensional.

F	PSO	GTO	DO	SO	FLA	HEOA	KOA	YDSE	GKSO
F1	6.72E−17	7.07E−18	7.07E−18	7.07E−18	7.07E−18	7.07E−18	7.07E−18	7.07E−18	3.32E−17
F2	7.07E−18	2.85E−16	1.09E−07	7.07E−18	3.74E−17	7.07E−18	7.07E−18	7.07E−18	**5.31E−02**
F3	7.07E−18	2.68E−12	7.07E−18	7.07E−18	7.07E−18	7.07E−18	7.07E−18	7.07E−18	**5.70E−01**
F4	8.31E−10	5.76E−03	1.20E−02	4.25E−16	2.15E−02	7.07E−18	7.07E−18	7.07E−18	**5.33E−01**
F5	7.39E−04	7.07E−18	7.50E−18	1.69E−10	3.79E−16	7.07E−18	7.07E−18	7.07E−18	2.07E−17
F6	3.65E−14	1.29E−17	3.74E−17	**3.57E−01**	2.27E−16	7.07E−18	7.07E−18	2.95E−17	2.02E−16
F7	1.84E−11	7.07E−18	7.07E−18	7.07E−18	8.99E−18	7.07E−18	7.07E−18	7.07E−18	1.01E−17
F8	4.16E−08	7.07E−18	8.46E−18	5.56E−12	1.08E−17	7.07E−18	7.07E−18	7.07E−18	7.50E−18
F9	**9.87E−02**	7.07E−18	7.07E−18	1.05E−13	6.72E−17	7.07E−18	7.07E−18	7.07E−18	7.97E−18
F10	2.87E−03	9.92E−16	3.95E−10	3.90E−02	1.05E−07	7.07E−18	7.07E−18	7.07E−18	5.93E−05
F11	5.67E−08	**5.46E−01**	**2.37E−01**	8.99E−17	1.41E−12	7.07E−18	7.07E−18	8.99E−18	**6.97E−01**
F12	5.27E−05	3.21E−03	1.98E−07	**1.25E−01**	3.54E−09	7.07E−18	7.07E−18	1.63E−17	1.19E−06
F13	5.82E−09	**5.65E−01**	3.02E−16	2.13E−08	7.07E−18	7.07E−18	7.07E−18	7.07E−18	9.95E−04
F14	8.99E−18	3.77E−12	7.07E−18	7.07E−18	7.07E−18	7.07E−18	7.07E−18	2.47E−17	4.96E−15
F15	1.93E−02	1.22E−02	1.07E−16	1.20E−02	7.07E−18	7.07E−18	7.07E−18	1.73E−09	4.45E−02
F16	**8.17E−01**	1.89E−04	3.84E−09	**6.37E−01**	1.32E−04	7.07E−18	7.07E−18	1.80E−16	**6.32E−02**
F17	1.21E−08	2.51E−14	1.12E−14	1.26E−11	4.71E−10	3.32E−17	7.07E−18	2.40E−16	1.63E−12
F18	7.07E−18	1.54E−10	7.50E−18	7.07E−18	7.07E−18	7.07E−18	7.07E−18	8.86E−16	1.16E−13
F19	2.92E−04	7.25E−14	1.91E−07	2.75E−09	8.17E−04	7.50E−18	7.07E−18	4.76E−16	1.20E−11
F20	**3.47E−01**	8.68E−10	6.80E−11	2.13E−07	3.21E−06	8.00E−17	7.07E−18	2.85E−16	1.95E−05
F21	5.40E−11	2.85E−16	7.50E−18	1.55E−14	2.20E−17	7.07E−18	7.07E−18	7.07E−18	1.21E−17
F22	7.07E−18	7.97E−18	7.07E−18	7.07E−18	7.07E−18	7.07E−18	7.07E−18	7.07E−18	1.89E−02
F23	1.20E−16	6.69E−16	1.45E−17	8.53E−13	3.80E−13	7.07E−18	7.07E−18	7.07E−18	3.38E−16
F24	7.07E−18	1.01E−17	7.07E−18	1.43E−16	7.07E−18	7.07E−18	7.07E−18	7.07E−18	1.73E−17
F25	3.23E−02	4.92E−04	7.29E−10	**6.62E−01**	3.58E−05	7.07E−18	7.07E−18	7.50E−18	1.64E−07
F26	2.42E−09	9.81E−11	3.26E−12	1.82E−14	1.12E−10	7.97E−18	7.07E−18	9.54E−18	1.55E−12
F27	2.44E−02	4.54E−09	2.81E−08	6.98E−13	1.50E−03	7.07E−18	7.07E−18	1.82E−14	3.79E−06
F28	9.92E−12	2.99E−04	**7.04E−02**	7.12E−17	6.86E−04	7.07E−18	7.07E−18	7.07E−18	8.37E−03
F29	2.84E−04	4.92E−11	9.69E−08	7.05E−07	**9.37E−01**	7.07E−18	7.07E−18	3.19E−16	1.98E−07
F30	6.10E−05	9.53E−17	3.19E−16	5.52E−03	1.65E−03	7.07E−18	7.07E−18	1.44E−04	1.12E−08

**Table 28:** Wilcoxon's rank sum test results between OBAs and SPO on CEC2017 with 50 dimensional.

F	PSO	GTO	DO	SO	FLA	HEOA	KOA	YDSE	GKSO
F1	7.07E−18	7.07E−18	7.07E−18	7.07E−18	7.07E−18	7.07E−18	7.07E−18	7.07E−18	7.07E−18
F2	7.07E−18	2.07E−17	4.36E−09	7.07E−18	3.32E−17	7.07E−18	7.07E−18	7.07E−18	2.64E−09
F3	7.07E−18	7.92E−05	8.46E−18	7.07E−18	7.07E−18	7.07E−18	7.07E−18	1.21E−17	1.99E−03
F4	2.51E−14	2.81E−11	2.31E−02	1.01E−17	2.92E−04	7.07E−18	7.07E−18	7.07E−18	2.04E−02
F5	4.06E−06	7.07E−18	7.07E−18	4.09E−11	7.07E−18	7.07E−18	7.07E−18	7.07E−18	7.07E−18
F6	9.43E−13	1.01E−17	7.07E−18	8.54E−03	1.43E−13	7.07E−18	7.07E−18	7.07E−18	2.20E−17
F7	3.02E−15	7.07E−18	7.07E−18	7.07E−18	7.07E−18	7.07E−18	7.07E−18	7.07E−18	7.07E−18
F8	1.58E−07	7.07E−18	7.07E−18	8.11E−13	7.07E−18	7.07E−18	7.07E−18	7.07E−18	7.07E−18
F9	5.30E−07	7.07E−18	7.07E−18	8.59E−04	7.07E−18	7.07E−18	7.07E−18	7.07E−18	4.73E−17
F10	**3.91E−01**	7.05E−07	4.60E−02	1.01E−05	3.00E−03	7.07E−18	7.07E−18	7.07E−18	**9.04E−01**
F11	1.77E−03	**4.26E−01**	3.51E−03	7.07E−18	4.76E−16	7.07E−18	7.07E−18	7.07E−18	6.32E−09
F12	2.26E−14	1.38E−05	6.81E−03	3.51E−03	3.90E−11	7.07E−18	7.07E−18	7.07E−18	6.85E−06
F13	1.92E−14	**3.57E−01**	7.07E−18	5.88E−14	7.07E−18	7.07E−18	7.07E−18	7.07E−18	**8.50E−01**
F14	2.33E−17	1.99E−12	7.97E−18	7.97E−18	7.07E−18	7.07E−18	7.07E−18	1.45E−09	1.80E−12
F15	1.41E−10	8.39E−05	3.32E−17	2.94E−13	7.07E−18	7.07E−18	7.07E−18	7.07E−18	**8.77E−01**
F16	1.17E−07	2.65E−13	1.15E−11	4.36E−09	4.16E−12	7.07E−18	7.07E−18	7.07E−18	4.28E−11
F17	1.85E−10	1.10E−12	1.50E−13	6.68E−10	8.01E−08	7.07E−18	7.07E−18	1.08E−17	8.97E−13
F18	1.29E−17	9.06E−10	8.99E−18	1.63E−17	7.07E−18	7.07E−18	7.07E−18	**1.36E−01**	5.49E−07
F19	**2.40E−01**	2.14E−14	1.50E−13	**8.54E−02**	1.08E−08	7.07E−18	7.07E−18	3.57E−15	1.10E−13
F20	1.74E−04	7.95E−10	4.69E−15	1.77E−07	2.42E−09	7.07E−18	7.07E−18	7.07E−18	1.12E−09
F21	6.17E−15	7.07E−18	7.07E−18	4.76E−16	7.07E−18	7.07E−18	7.07E−18	7.07E−18	7.07E−18
F22	**6.82E−02**	2.02E−10	1.31E−07	3.69E−09	3.11E−06	7.07E−18	7.07E−18	7.07E−18	**1.23E−01**
F23	7.07E−18	8.99E−18	7.07E−18	2.86E−15	2.95E−17	7.07E−18	7.07E−18	7.07E−18	8.00E−17
F24	7.07E−18	7.07E−18	7.07E−18	2.02E−16	7.07E−18	7.07E−18	7.07E−18	7.07E−18	1.08E−17
F25	4.11E−04	5.30E−12	3.96E−02	6.72E−17	**7.38E−01**	7.07E−18	7.07E−18	7.07E−18	**7.75E−01**
F26	**1.78E−01**	1.17E−09	2.94E−13	1.86E−02	**2.93E−01**	7.07E−18	7.07E−18	7.08E−16	5.74E−04
F27	1.18E−05	2.56E−11	1.93E−11	4.06E−14	1.70E−04	7.07E−18	7.07E−18	2.14E−16	6.56E−07
F28	8.05E−14	1.24E−15	**2.08E−01**	7.07E−18	1.27E−03	7.07E−18	7.07E−18	7.07E−18	**1.91E−01**
F29	1.12E−04	5.89E−04	8.80E−04	7.21E−04	1.04E−05	7.07E−18	7.07E−18	7.07E−18	3.29E−08
F30	1.35E−16	7.07E−18	7.12E−11	1.13E−16	7.07E−18	7.07E−18	7.07E−18	1.10E−03	2.05E−15

**Table 29:** Wilcoxon's rank sum test results between OBAs and SPO on CEC2017 with 100 dimensional.

F	PSO	GTO	DO	SO	FLA	HEOA	KOA	YDSE	GKSO
F1	7.07E−18	7.07E−18	7.07E−18	7.07E−18	7.07E−18	7.07E−18	7.07E−18	7.07E−18	7.07E−18
F2	2.47E−17	1.37E−17	3.08E−07	7.07E−18	6.13E−12	7.07E−18	7.07E−18	7.07E−18	1.64E−14
F3	7.07E−18	2.99E−09	7.07E−18	1.45E−17	1.08E−17	7.97E−18	7.07E−18	2.53E−02	2.04E−03
F4	7.07E−18	7.07E−18	1.12E−14	7.07E−18	8.99E−17	7.07E−18	7.07E−18	7.07E−18	5.84E−15
F5	1.70E−16	7.97E−18	7.50E−18	6.32E−16	7.07E−18	7.07E−18	7.07E−18	7.07E−18	8.99E−18
F6	6.14E−03	7.97E−18	1.29E−17	**3.26E−01**	**7.54E−01**	7.07E−18	7.07E−18	7.07E−18	1.29E−17
F7	3.38E−15	7.07E−18	7.07E−18	7.07E−18	7.07E−18	7.07E−18	7.07E−18	7.07E−18	7.07E−18
F8	8.38E−16	7.07E−18	7.07E−18	1.60E−16	7.07E−18	7.07E−18	7.07E−18	7.07E−18	7.07E−18
F9	7.07E−18	2.02E−16	7.07E−18	9.33E−08	7.07E−18	7.07E−18	7.07E−18	7.07E−18	1.36E−13
F10	4.16E−02	3.35E−03	1.47E−07	7.07E−18	6.12E−10	7.07E−18	7.07E−18	7.07E−18	1.39E−15
F11	7.07E−18	1.01E−17	1.29E−17	7.07E−18	7.07E−18	7.07E−18	7.07E−18	7.07E−18	2.76E−02
F12	7.07E−18	2.99E−04	3.07E−03	1.21E−17	3.10E−12	7.07E−18	7.07E−18	7.07E−18	1.30E−05
F13	7.07E−18	7.07E−18	7.07E−18	7.07E−18	7.07E−18	7.07E−18	7.07E−18	7.07E−18	7.68E−15
F14	8.46E−18	1.23E−09	1.29E−17	7.07E−18	7.07E−18	7.07E−18	7.07E−18	2.76E−04	2.71E−02
F15	1.06E−14	2.29E−04	7.07E−18	7.07E−18	7.07E−18	7.07E−18	7.07E−18	7.07E−18	3.42E−12
F16	**8.60E−01**	**4.76E−01**	1.58E−09	**1.49E−01**	2.63E−06	7.07E−18	7.07E−18	7.07E−18	**2.60E−01**
F17	8.99E−18	1.64E−15	3.98E−15	5.97E−16	1.63E−17	7.07E−18	7.07E−18	7.07E−18	2.29E−15
F18	7.07E−18	3.19E−16	7.07E−18	7.07E−18	7.50E−18	7.07E−18	7.07E−18	1.69E−06	1.15E−06
F19	2.95E−11	2.40E−16	7.34E−13	2.07E−05	3.46E−14	7.07E−18	7.07E−18	7.07E−18	7.55E−06
F20	8.55E−11	9.88E−10	1.64E−15	7.07E−18	1.15E−11	7.97E−18	7.07E−18	7.07E−18	9.81E−11
F21	7.07E−18	7.07E−18	7.07E−18	7.07E−18	7.07E−18	7.07E−18	7.07E−18	7.07E−18	7.07E−18
F22	4.66E−03	1.11E−02	**9.19E−02**	7.07E−18	1.69E−10	7.07E−18	7.07E−18	7.07E−18	3.29E−08
F23	7.07E−18	8.46E−18	2.62E−17	1.03E−10	4.36E−12	7.07E−18	7.07E−18	7.07E−18	2.07E−17
F24	7.07E−18	7.07E−18	7.07E−18	7.07E−18	1.37E−17	7.07E−18	7.05E−18	7.07E−18	7.07E−18
F25	7.07E−18	7.07E−18	8.94E−14	7.07E−18	7.50E−18	7.07E−18	7.07E−18	7.07E−18	2.31E−12
F26	2.18E−03	1.55E−15	2.05E−15	5.64E−03	1.48E−02	7.07E−18	7.03E−18	7.50E−18	1.10E−13
F27	**1.53E−01**	**5.84E−01**	**1.06E−01**	6.98E−10	2.09E−12	7.07E−18	7.07E−18	3.32E−17	1.30E−03
F28	8.00E−17	7.07E−18	3.12E−02	7.07E−18	1.80E−16	7.07E−18	7.07E−18	7.07E−18	3.51E−04
F29	4.90E−13	**8.71E−01**	**4.06E−01**	2.44E−02	1.02E−02	7.07E−18	7.07E−18	7.07E−18	1.02E−02
F30	1.70E−04	7.07E−18	1.82E−14	5.02E−17	7.07E−18	7.07E−18	7.07E−18	7.07E−18	7.50E−18

**Table 30:** Wilcoxon's rank sum test results between OBAs and SPO on CEC2019.

F	PSO	GTO	DO	SO	FLA	HEOA	KOA	YDSE	GKSO
F1	8.46E−18	3.31E−20	1.03E−10	8.03E−03	3.31E−20	3.31E−20	7.07E−18	2.57E−02	4.73E−20
F2	1.08E−17	6.23E−18	1.08E−17	2.37E−06	**1.99E−01**	3.33E−18	7.07E−18	1.84E−11	7.05E−18
F3	6.56E−07	5.46E−08	9.42E−14	3.53E−17	9.53E−17	3.02E−15	7.07E−18	7.07E−18	**6.82E−02**
F4	9.26E−03	8.38E−16	5.98E−17	6.31E−13	1.18E−10	7.07E−18	7.07E−18	5.02E−17	5.53E−15
F5	7.07E−18	7.07E−18	7.07E−18	7.50E−18	7.07E−18	7.07E−18	7.07E−18	7.07E−18	7.07E−18
F6	8.20E−03	7.07E−18	7.07E−18	7.50E−18	7.07E−18	7.07E−18	7.07E−18	7.07E−18	7.50E−18
F7	2.56E−03	6.80E−11	1.58E−09	**7.38E−01**	**1.17E−01**	3.77E−15	7.07E−18	4.76E−14	5.93E−05
F8	1.79E−02	2.64E−09	3.96E−12	8.05E−06	5.74E−04	2.33E−17	7.07E−18	5.64E−17	5.29E−03
F9	1.54E−02	1.54E−10	2.81E−11	5.98E−17	1.01E−17	2.71E−15	7.07E−18	1.95E−17	1.24E−06
F10	7.55E−17	3.26E−13	5.98E−17	7.07E−18	7.07E−18	1.45E−17	7.07E−18	7.07E−18	9.53E−17

**Table 31:** Wilcoxon's rank sum test results between OBAs and SPO on CEC2020.

F	PSO	GTO	DO	SO	FLA	HEOA	KOA	YDSE	GKSO
F1	2.95E−14	7.15E−09	7.07E−18	7.07E−18	7.07E−18	7.07E−18	7.07E−18	7.07E−18	1.29E−10
F2	1.63E−06	4.71E−10	**9.75E−01**	3.24E−11	1.13E−16	7.97E−18	7.07E−18	7.97E−18	2.99E−04
F3	2.79E−13	7.07E−18	7.07E−18	7.07E−18	3.78E−10	7.07E−18	7.07E−18	7.07E−18	7.07E−18
F4	1.99E−12	2.20E−17	3.57E−15	2.42E−12	4.68E−04	7.07E−18	7.07E−18	7.50E−18	1.76E−13
F5	7.07E−18	1.80E−09	7.07E−18	1.14E−17	7.07E−18	7.07E−18	7.07E−18	1.29E−17	3.48E−05
F6	**5.84E−02**	**6.32E−02**	4.18E−03	**7.49E−01**	2.65E−13	7.50E−18	7.07E−18	1.28E−06	**6.62E−01**
F7	8.46E−18	3.14E−03	7.50E−18	5.02E−17	7.07E−18	7.07E−18	7.07E−18	4.21E−15	6.27E−03
F8	4.65E−13	3.58E−05	7.07E−18	7.07E−18	7.07E−18	7.07E−18	7.07E−18	7.07E−18	5.89E−03
F9	1.51E−16	1.45E−17	7.07E−18	2.86E−15	7.07E−18	7.07E−18	7.07E−18	7.07E−18	1.84E−17
F10	4.30E−02	7.45E−09	**6.17E−01**	1.12E−04	**4.06E−01**	7.07E−18	7.07E−18	**9.31E−01**	3.29E−02

**Table 32:** Wilcoxon's rank sum test results between OBAs and SPO on CEC2022 with 10 dimensional.

F	PSO	GTO	DO	SO	FLA	HEOA	KOA	YDSE	GKSO
F1	7.07E−18	4.16E−12	7.07E−18	7.07E−18	7.07E−18	7.07E−18	7.07E−18	7.07E−18	9.38E−16
F2	9.42E−18	1.61E−17	3.32E−17	8.48E−17	7.07E−18	7.07E−18	7.07E−18	7.07E−18	2.14E−16
F3	8.38E−16	6.89E−15	1.50E−13	**3.29E−01**	**1.03E−01**	7.07E−18	7.07E−18	1.84E−17	8.41E−07
F4	2.03E−14	1.29E−17	7.97E−18	2.95E−17	7.07E−18	7.07E−18	7.07E−18	7.07E−18	2.33E−17
F5	3.58E−05	7.07E−18	7.07E−18	7.07E−18	7.07E−18	7.07E−18	7.07E−18	7.07E−18	7.07E−18
F6	1.07E−16	6.66E−05	7.07E−18	7.49E−16	7.07E−18	7.07E−18	7.07E−18	1.92E−14	3.85E−14
F7	1.47E−07	5.89E−03	4.27E−03	**8.12E−01**	2.56E−07	8.99E−18	7.07E−18	1.20E−11	**5.33E−01**
F8	**5.46E−01**	1.13E−02	8.19E−12	3.51E−03	**5.79E−01**	9.54E−18	7.07E−18	4.49E−11	**2.26E−01**
F9	**2.97E−01**	1.99E−09	3.90E−02	7.42E−06	4.73E−17	7.07E−18	7.07E−18	7.07E−18	4.90E−13
F10	1.12E−08	6.07E−09	6.00E−13	6.86E−09	1.93E−10	1.21E−17	8.48E−17	2.16E−13	**9.20E−01**
F11	5.05E−12	4.28E−14	3.56E−11	7.30E−07	1.40E−04	1.18E−05	7.07E−18	**2.11E−01**	1.68E−11
F12	1.12E−13	1.76E−02	1.74E−15	7.55E−17	5.43E−05	2.02E−16	7.07E−18	3.34E−02	2.45E−06

**Table 33:** Wilcoxon's rank sum test results between OBAs and SPO on CEC2022 with 20 dimensional.

F	PSO	GTO	DO	SO	FLA	HEOA	KOA	YDSE	GKSO
F1	7.07E−18	7.50E−18	7.07E−18	7.07E−18	7.07E−18	7.07E−18	7.07E−18	7.07E−18	2.76E−04
F2	1.35E−02	3.83E−02	1.85E−03	5.43E−05	2.99E−04	7.07E−18	7.07E−18	1.52E−11	**1.69E−01**
F3	1.69E−06	1.51E−16	1.51E−16	**5.66E−02**	2.02E−16	7.07E−18	7.07E−18	3.38E−16	4.90E−13
F4	2.42E−10	7.97E−18	1.54E−17	2.81E−12	7.07E−18	7.07E−18	7.07E−18	7.07E−18	3.74E−17
F5	4.76E−14	7.07E−18	7.07E−18	9.53E−17	7.50E−18	7.07E−18	7.07E−18	8.99E−18	1.54E−17
F6	8.56E−15	6.63E−13	5.15E−13	2.03E−14	7.07E−18	7.07E−18	7.07E−18	7.07E−18	2.03E−14
F7	2.14E−06	3.51E−03	7.80E−11	**4.88E−01**	**5.93E−01**	5.98E−17	7.07E−18	4.92E−11	**1.61E−01**
F8	8.42E−09	2.69E−04	5.64E−16	1.76E−13	3.42E−04	7.07E−18	7.07E−18	7.07E−18	3.18E−02
F9	9.16E−06	1.09E−11	3.29E−08	**3.16E−01**	6.32E−16	7.07E−18	7.07E−18	7.07E−18	2.32E−09
F10	3.08E−07	6.32E−09	5.37E−10	5.25E−08	4.06E−06	3.58E−16	5.32E−17	2.22E−11	**4.67E−01**
F11	7.07E−18	**7.33E−01**	3.02E−16	7.07E−18	7.07E−18	7.07E−18	7.07E−18	7.07E−18	7.55E−06
F12	1.65E−09	8.31E−10	1.28E−09	3.79E−16	**1.82E−01**	1.91E−16	7.07E−18	3.92E−05	6.69E−04

As can be seen from Tables 20, 21, 22, 23, 24, 25, 26, the number of cases in which the SPO algorithm has a significant advantage over other MBAs algorithms in the 30-dimensional CEC2017,50-dimensional CEC2017, and 100-dimensional CEC2017 tests are 228/240,229/240,223/240, that means in more than 95.8% of the cases, the SPO algorithm has a significant advantage. In CEC2019, CEC2020, CEC2022 in 10 dimensions and CEC2022 in 20 dimensions, the numbers with significant benefits are 77/80,76/80,89/96,93/96, respectively, which means The SPO algorithm has substantial advantages in 95.2% of the cases.

From Tables 27, 28, 29, 30, 31, 32, 33, it can be seen that the SPO algorithm has a significant advantage over the other OBAs algorithms in the 30-dimensional CEC2017,50-dimensional CEC2017, and 100-dimensional CEC2017 tests in the number of substantial benefits are 253/270,253/270, 258/270, that means in more than 94.3% of the cases, the SPO algorithm has a significant advantage. In CEC2019, CEC2020, 10-dimensional CEC2022, and 20-dimensional CEC2022, the considerable benefits are 86/90,82/90,98/108,99/108, respectively, which means The SPO algorithm has significant advantages in 92.2% of the cases.

In general, the SPO algorithm has a significant advantage in 94.6% of cases compared to all algorithms.

#### The Friedman test

**Figure 14:**
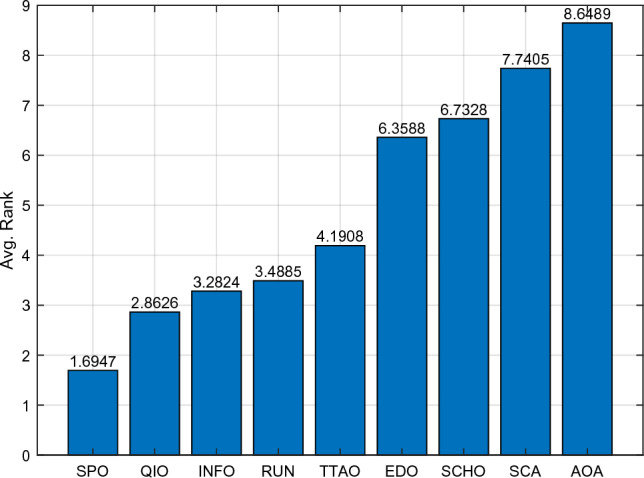
The overall Friedman rank of SPO and MBAs.

Figure 14 shows the Friedman test results of the SPO algorithm and 8 MBAs algorithms on all 131 test functions. As can be seen from the figure, the SPO algorithm won first place with an absolute advantage of 1.6947. The final ranking is SPO > QIO > INFO > RUN > TTAO > EDO > SCHO > SCA > AOA. Table 34 shows the specific Friedman test results on each test set. As the table shows, SPO has a clear advantage on all the test sets and is ranked first.

**Table 34:** Friedman test results with MBAs and SPO.

CEC	Type	SCA	RUN	AOA	INFO	SCHO	EDO	TTAO	QIO	SPO
2017 (30 dim)	Avg. rank	7.8276	3.6207	8.6552	3.1724	6.6897	6.4828	4.1034	2.6552	**1.7931**
	Overall rank	8	4	9	3	7	6	5	2	**1**
2017 (50 dim)	Avg. rank	7.7586	3.4483	8.7241	3.0690	6.5172	6.7931	4.3448	2.8621	**1.4828**
	Overall rank	8	4	9	3	6	7	5	2	**1**
2017 (100 dim)	Avg. rank	7.8966	3.1724	8.4483	3.3103	6.3793	7.0000	4.1724	3.0690	**1.5517**
	Overall rank	8	3	9	4	6	7	5	2	**1**
2019	Avg. rank	8.0000	3.4000	8.3000	3.6000	6.3000	6.0000	4.5000	2.8000	**2.1000**
	Overall rank	8	3	9	4	7	6	5	2	**1**
2020	Avg. rank	7.9000	3.7000	8.9000	3.6000	6.7000	5.4000	4.2000	2.8000	**1.8000**
	Overall rank	8	4	9	3	7	6	5	2	**1**
2022 (10 dim)	Avg. rank	7.1667	4.4167	8.7500	3.5833	7.8333	4.9167	3.8333	2.6667	**1.8333**
	Overall rank	7	5	9	3	8	6	4	2	**1**
2022 (20 dim)	Avg. Rank	7.3333	3.0000	8.9167	3.1667	7.5000	6.0000	4.1667	3.1667	**1.7500**
	Overall rank	6	2	8	3	7	5	4	3	**1**

**Figure 15:**
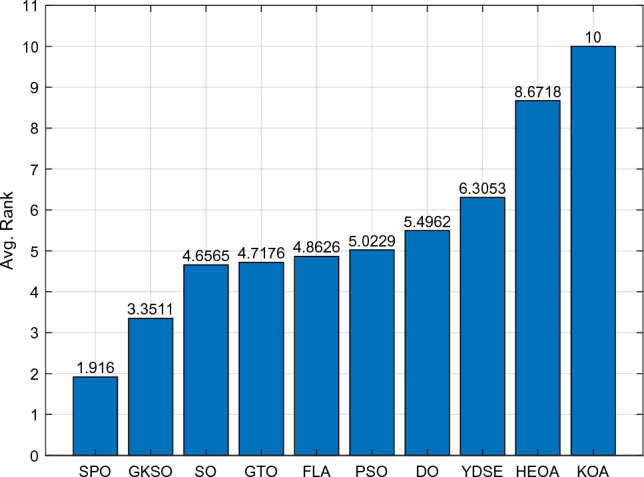
The overall Friedman rank of SPO and MBAs.

Figure 15 illustrates the Friedman test results for the SPO algorithm and the nine OBAs algorithms on all 131 test functions. Similar to the test results for the MBAs algorithms, the SPO algorithm again takes the first place by a wide margin, the GKSO algorithm takes the second place, and the SO, GTO, FLA, and PSO algorithms are closer to each other in terms of performance. The final ranking is SPO > GKSO > SO > GTO > FLA > PSO > DO > YDSE > HEOA > KOA. Table 35 demonstrates the results of Friedman's test for the SPO algorithm and OBAs algorithm on the CEC test function. The table shows that the SPO algorithm has a significant advantage over the other OBAs algorithms and has a much smaller final ranking on each test set.

**Table 35:** Friedman test results with OBAs and SPO.

CEC	Type	PSO	GTO	DO	SO	FLA	HEOA	KOA	YDSE	GKSO	SPO
2017 (30 dim)	Avg. rank	4.7241	4.6552	6.0690	4.2759	4.8966	9.0000	10.0000	6.0690	3.3103	**2.0000**
	Overall rank	5	4	7	3	6	8	9	7	2	**1**
2017 (50 dim)	Avg. rank	4.8966	4.9310	5.2414	4.4828	4.3448	8.8621	10.0000	7.0000	3.4138	**1.8276**
	Overall rank	5	6	7	4	3	9	10	8	2	**1**
2017 (100 dim)	Avg. rank	5.6897	4.3103	4.4138	5.0690	4.7931	8.5862	10.0000	7.1724	3.1724	**1.7931**
	Overall rank	7	3	4	6	5	9	10	8	2	**1**
2019	Avg. rank	4.7000	4.3000	6.7000	5.6000	4.8000	6.8000	10.0000	6.8000	3.4000	**1.9000**
	Overall rank	4	3	7	6	5	8	9	8	2	**1**
2020	Avg. rank	5.1000	5.8000	5.4000	4.1000	4.5000	8.8000	10.0000	5.1000	4.1000	**2.1000**
	Overall rank	4	6	5	2	3	7	8	4	2	**1**
2022 (10 dim)	Avg. rank	4.9167	4.4167	6.5833	4.7500	5.7500	9.0000	10.0000	4.0833	3.1667	**2.3333**
	Overall rank	6	4	8	5	7	9	10	3	2	**1**
2022 (20 dim)	Avg. rank	4.7500	5.0833	5.3333	4.5833	5.6667	8.7500	10.0000	5.9167	3.2500	**1.6667**
	Overall rank	4	5	6	3	7	9	10	8	2	**1**

## Engineering problems tests

Several common engineering problems are selected in this paper to verify the performance of SPO algorithms and verify their effectiveness in real engineering. Among the comparison algorithms, some algorithms are selected from the two types of algorithms in the previous section, and some algorithms that have been verified for a long time in real engineering are added. In the testing process, 50 rounds of the same test were performed for each algorithm with the same parameters as in the previous section.

### Tension/compression spring design problem

**Figure 16:**
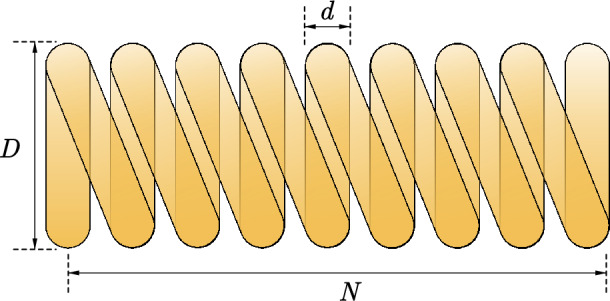
Schematic of the tension/compression spring design problem.

**Figure 17:**
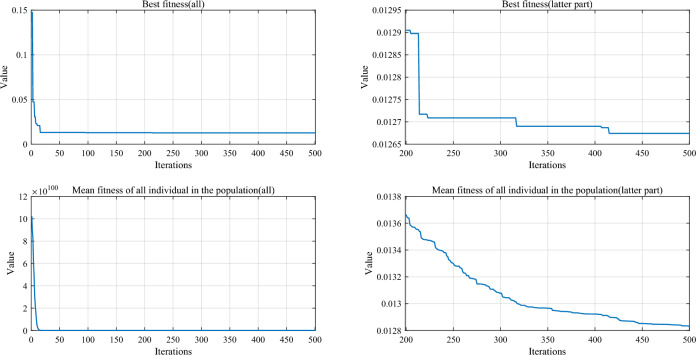
Convergence curves of the SPO algorithm for the tension/compression spring design problem.

The tension/compression spring design problem optimizes the spring-mass under four constraints. The problem schematic is shown in Fig. 16, and its optimization variables include the diameter of the spring *d*, the diameter of the coils *D,* and the number of loops *N*. The mathematical model can be referred to in the paper^77^. The experiment results are shown in Table 36. The best result has been marked in bold. The convergence curve of the SPO algorithm in the experiment is shown in Fig. 17.

**Table 36:** Results of the comparative algorithms for solving the tension/compression spring design problem.

Methods	*d*	*D*	*N*	Best fitness	Mean best fitness	Rank
PSO^29^	0.05170	0.35697	11.27420	**0.01267**	0.01297	7
HHO^78^	0.05201	0.36448	10.84789	**0.01267**	0.01395	9
SSA^79^	0.05183	0.36017	11.08925	**0.01267**	0.01400	10
GWO^80^	0.05210	0.36658	10.74693	0.01268	0.01280	5
GTO^72^	0.05162	0.35505	11.38733	**0.01267**	0.01277	4
SO^73^	0.05169	0.35676	11.28677	**0.01267**	0.01317	8
INFO^68^	0.05107	0.34191	12.21271	**0.01267**	0.01286	6
GKSO^75^	0.05172	0.35740	11.24884	**0.01267**	0.01275	3
TTAO^71^	0.05170	0.35708	11.26769	**0.01267**	0.01273	2
SPO	0.05188	0.36133	11.02805	**0.01267**	**0.01271**	1

The optimization results in Table 36 show that although most algorithms achieve the optimal value at the best time, the SPO algorithm is more stable than the others with the minimum mean best fitness. It can also be seen from the convergence curve in Fig. 16 that the SPO algorithm can converge quickly for the spring compression problem, and at the same time, it can perform a small range of optimization searches for the optimal position in subsequent iterations to continuously improve the optimal position.

### Gear train design problem

**Figure 18:**
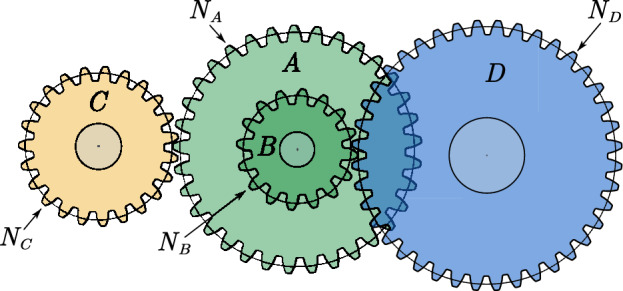
Schematic of the gear train design problem.

**Figure 19:**
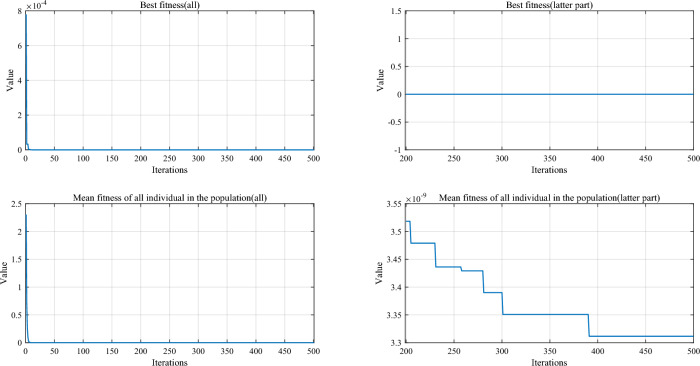
Convergence curves of the SPO algorithm for the gear train design problem.

The gear train design problem is a common problem in mechanical engineering. As shown in Fig. 18, its optimization variable is the number of gears of four gears. Its mathematical model can be referred to in the paper^81^. The experiment results are shown in Table 37. The best result has been marked in bold. The convergence curve of the SPO algorithm in the experiment is shown in Fig. 19.

**Table 37:** Results of the comparative algorithms for solving the gear train design problem.

Methods	*N*_*A*_	*N*_*B*_	*N*_*C*_	*N*_*D*_	Best fitness	Mean best fitness	Rank
PSO^29^	42.54635	18.69685	15.60369	49.44784	**2.70E−12**	2.08E−08	10
HHO^78^	43.44107	18.68396	15.92392	48.56077	**2.70E−12**	2.06E−09	8
SSA^79^	43.43379	15.74257	19.47048	48.93938	**2.70E−12**	5.87E−09	9
GWO^80^	42.92834	18.62713	16.29810	48.90087	**2.70E−12**	5.03E−10	4
GTO^72^	43.28484	15.56129	19.44880	49.32341	**2.70E−12**	1.32E−09	6
SO^73^	48.97904	16.40031	18.73988	42.79534	**2.70E−12**	3.58E−10	3
INFO^68^	49.19001	18.59330	16.06992	43.06100	**2.70E−12**	1.34E−09	7
GKSO^75^	48.76565	18.85895	16.08413	43.02876	**2.70E−12**	6.19E−10	5
TTAO^71^	48.53651	19.30084	16.28185	43.20750	**2.70E−12**	1.23E−10	2
SPO	42.52878	15.64835	19.11726	48.84986	**2.70E−12**	**5.15E−12**	1

From the results in Table 37, all the algorithms search for the optimal location for this problem in the best case, but the SPO algorithm is far better than the other algorithms in terms of the mean best of 50 round tests, and the mean best fitness is more than two orders of magnitude lower than the other algorithms. From the convergence shown in Fig. 19, the SPO algorithm found the optimal problem using only a smaller number of iterations.

### Pressure vessel design problem

**Figure 20:**
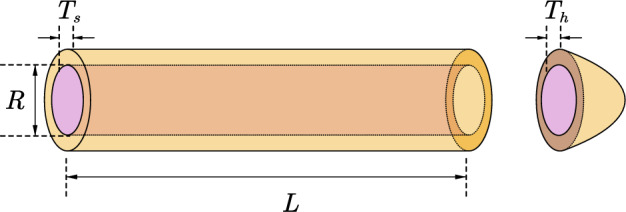
Schematic of the pressure vessel design problem.

**Figure 21:**
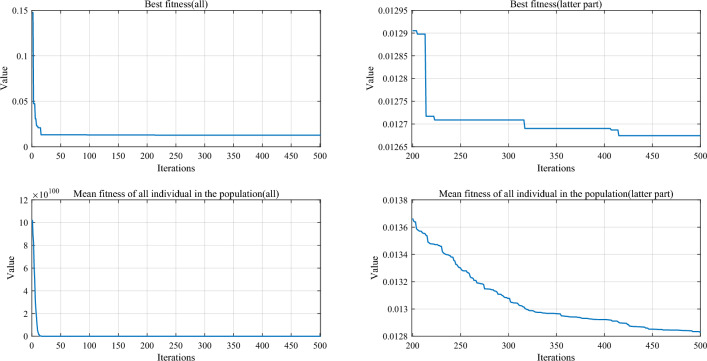
Convergence curves of the SPO algorithm for the pressure vessel design problem.

Pressure vessel design problems are common in the actual manufacturing process. It mainly solves using the minimum cost to withstand a certain pressure. The schematic diagram of the problem is shown in Fig. 20, and the optimization variables mainly include four, which are shell thickness (*T*_*s*_), head thickness (*T*_*h*_), diameter (*R*), and cylindrical length (*L*). Its mathematical model can be referred to in the paper^82^. The experiment results are shown in Table 38. The best result has been marked in bold. The convergence curve of the SPO algorithm in the experiment is shown in Fig. 21.

**Table 38:** Results of the comparative algorithms for solving the pressure vessel design problem.

Methods	*T*_*s*_	*T*_*h*_	*R*	*L*	Best fitness	Mean best fitness	Rank
PSO^29^	0.77817	0.38465	40.31962	200.00000	**5885.33277**	6270.45551	3
HHO^78^	0.85848	0.42453	44.35382	150.55896	6052.93173	8828.73772	10
SSA^79^	0.77939	0.38526	40.38315	199.11743	5887.43210	6464.07619	9
GWO^80^	0.78007	0.38640	40.41534	198.76865	5893.43547	6032.72757	2
GTO^72^	0.77817	0.38465	40.31962	200.00000	**5885.33277**	6334.98407	6
SO^73^	0.77817	0.38486	40.31962	200.00000	5885.94249	6362.46422	7
INFO^68^	0.77817	0.38465	40.31971	199.99881	5885.37703	6312.53855	5
GKSO^75^	0.78403	0.38754	40.62303	195.81900	5895.42723	6289.04805	4
TTAO^71^	0.78624	0.39477	40.70377	194.93313	5926.48904	6459.13174	8
SPO	0.78048	0.38808	40.39967	199.69642	5918.65205	**6016.49822**	1

From the results in Table 38, the SPO algorithm is ranked first and much better than the other algorithms in the mean best fitness of 50 round tests, although it does not perform as well as the PSO and GTO algorithms in the best case. Meanwhile, from the convergence curve situation in Fig. 21, it can be seen that the SPO algorithm can search near the optimal position very quickly and keep optimizing the optimal position in subsequent iterations, proving the SPO algorithm's effectiveness in this problem.

### Planetary-gear-train design optimization problem

**Figure 22:**
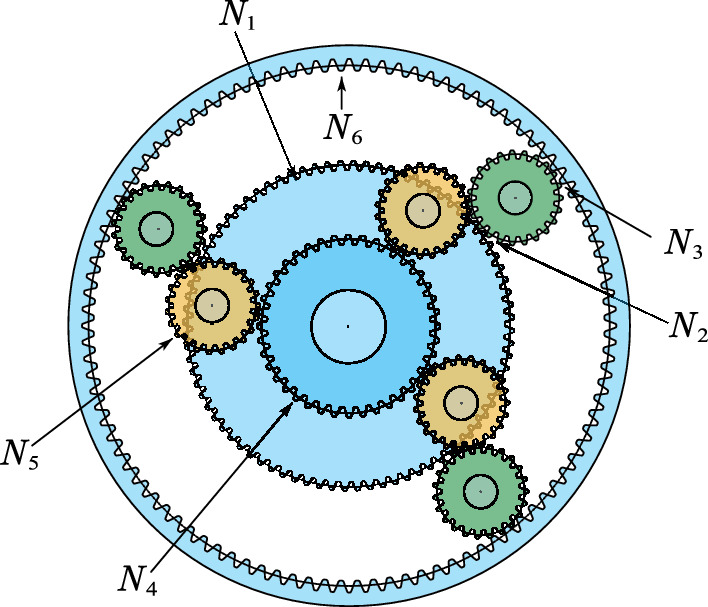
Schematic of the planetary-gear-train design optimization problem.

**Figure 23:**
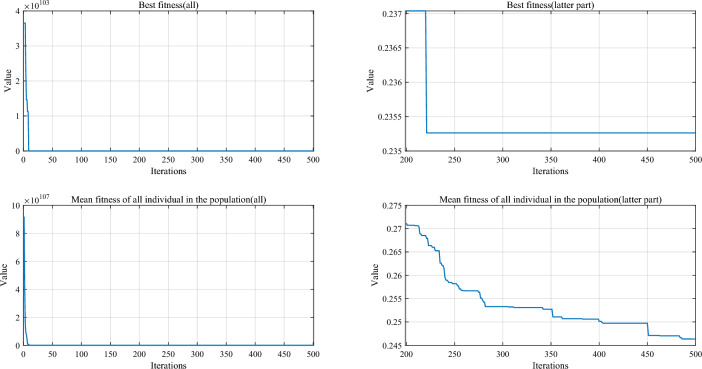
Convergence curves of the SPO algorithm for the planetary-gear-train design optimization problem.

The planetary-gear-train design optimization problem is a common problem in the automotive design process. The main objective is to reduce the maximum error of the transmission ratio during automobile use. The schematic diagram of the problem is shown in Fig. 22, and the optimization variables mainly include nine, six of which are the number of gears required to be integers. Its mathematical model can be referred to in the paper^83^. The experiment results are shown in Table 39. The best result has been marked in bold. The convergence curve of the SPO algorithm in the experiment is shown in Fig. 23.

**Table 39:** Results of the comparative algorithms for solving the planetary-gear-train design optimization problem.

Methods	*N*_1_	*N*_2_	*N*_3_	*N*_4_	*N*_5_	*N*_6_	*m*_1_	*m*_2_	*p*	Best fitness	Mean best fitness	Rank
PSO^29^	22.0541	23.6011	29.7718	31.6084	16.2517	92.3573	4.5719	2.3668	1.0742	0.23545	3.78E+99	9
HHO^78^	40.0445	31.7888	22.4154	32.4006	25.2903	92.3376	1.5934	1.8951	1.3978	0.23545	0.24157	5
SSA^79^	17.0000	14.0000	17.3210	23.7018	17.5120	69.3589	5.6720	1.2786	1.2867	0.23547	2.55E+102	10
GWO^80^	39.6710	31.7971	22.3549	32.0063	18.6973	92.2576	2.1119	2.1857	1.7922	0.23545	0.24026	2
GTO^72^	25.3144	23.1277	18.6416	23.5809	18.5473	68.7357	1.2450	1.2972	1.2625	0.23586	0.24362	7
SO^73^	42.5621	25.1962	16.0506	31.8812	14.9071	91.9831	1.8363	1.0200	2.4639	0.23535	0.24306	6
INFO^68^	21.7656	15.8270	15.1758	23.6826	15.2340	68.8519	4.3961	1.1148	1.4564	0.23545	0.24406	8
GKSO^75^	40.0963	31.6313	22.4440	31.7962	26.3233	91.8754	2.0014	2.0638	1.6825	0.23545	0.24080	4
TTAO^71^	39.8306	32.0584	22.1348	31.8638	15.0408	91.8701	1.1314	1.0000	1.7769	0.23545	0.24036	3
SPO	42.0244	28.7830	19.4186	31.7094	19.1782	91.8750	2.2316	2.0248	1.4861	**0.23526**	**0.23888**	1

From the results in Table 39, the SPO algorithm is ranked first in best fitness and mean best fitness. It can be seen that both PSO and SSA algorithms have an enormous value of average fitness, which indicates that these two algorithms have not been able to optimize the sub-problem effectively in some cases. The convergence curves in Fig. 23 show that the SPO algorithm can still converge quickly compared to the other three engineering problems despite increasing the number of variables. This result shows that the SPO algorithm also performs well when facing complex problems.

## Spacecraft trajectory optimization using SPO

**Figure 24:**
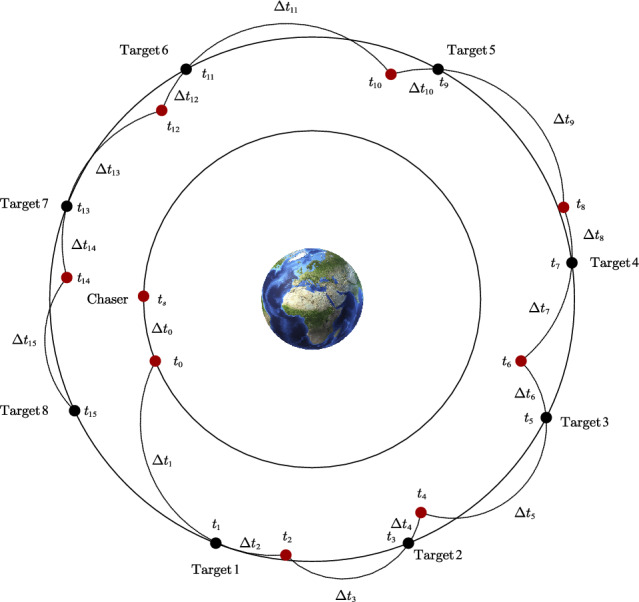
Schematic of the spacecraft trajectory optimization problem.

With the continuous development of aerospace technology, spacecraft has become an essential part of the combination of production and life. Research on spacecraft trajectory is also increasingly prosperous, and spacecraft trajectory optimization is necessary^84^. The spacecraft trajectory optimization problem referred to in this section relates to the trajectory optimization problem of a single spacecraft for multiple spacecraft in the same orbital plane to leap, and its schematic diagram is shown in Fig. 24.

As can be seen from the figure, the chaser needs to flyby the target sequentially, where the main problem is that all spacecraft are in motion, which means that the position of the target is different at each moment. At the same time, the main variable dt in the problem belongs to an extensive range of continuous variables, and the number of variables is large, which makes the spacecraft trajectory optimization problem highly complex.

The objective function of the spacecraft trajectory optimization problem can be expressed as

29 $$ \begin{array}{*{20}c} {{\text{Minimize}}} & f \\ \end{array} (x) = \sum\limits_{i = 0}^{n - 1} {dv_{2i} } $$

where *dv*_2*i*_ denotes the amount of change in the velocity of the chaser spacecraft at the moment *t*_2*i*_, which can be calculated by solving the Lambert problem consisting of the time interval *dt*_2*i*+1_ and the positions of the chaser spacecraft at the moment *t*_2*i*_, and *t*_2*i*+1_.

The constraints of the problem consist of three main categories. The first category is time constraints, where all intervals should exceed a specified maximum time.

30 $$ \Delta t_{min} \le \Delta t_{i} \le \Delta t_{max} $$

The second type of constraint is the position constraint, where the position of the two spacecraft should be less than the minimum requirement when the chaser flyby the target.

31 $$ \left\| {R_{chaser} - R_{target} } \right\| \le \varepsilon_{r} $$

The third type of constraint is the velocity constraint, where each velocity increment should be less than the maximum velocity increment the chaser can apply.

32 $$ \left| {dv_{i} } \right| \le dv_{max} $$

The initial values for all spacecraft are shown in Table 40. To further validate the performance of SPO, 11 recently published competitive algorithms are additionally selected for comparison in this section, all of which were published after 2023 and 4 of which were just published in 2024. The parameters of all the algorithms were selected according to the criteria of the published papers; the population size was set to 60, and the number of iterations was set to 300. In order to verify the robustness of the algorithms, we tested all the algorithms with 50 randomized tests. The specific test results are shown in Table 41.

**Table 40:** Results of the comparative algorithms for solving the spacecraft trajectory optimization problem.

Spacecraft	X (km)	Y (km)	Z (km)	Vx (km/s)	Vy (km/s)	Vz (km/s)
Chaser	12,189.070000	− 8989.090101	− 19,102.788842	3.501862	0.860841	1.829380
Target1	26,378.140000	0.000000	0.000000	0.000000	1.655126	3.517322
Target2	18,652.161669	7941.704182	16,876.980465	− 2.748727	1.170351	2.487122
Target3	0.000000	11,231.265762	23,867.654666	− 3.887287	0.000000	0.000000
Target4	− 18,652.161669	7941.704182	16,876.980465	− 2.748727	− 1.170351	− 2.487122
Target5	− 26,378.140000	0.000000	0.000000	0.000000	− 1.655126	− 3.517322
Target6	− 18,652.161669	− 7941.704182	− 16,876.980465	2.748727	− 1.170351	− 2.487122
Target7	0.000000	− 11,231.265762	− 23,867.654666	3.887287	0.000000	0.000000
Target8	18,652.161669	− 7941.704182	− 16,876.980465	2.748727	1.170351	2.487122

**Table 41:** Results of the comparative algorithms for solving the spacecraft trajectory optimization problem.

Methods	Year	Mean	Std	Best	Worst
SPO	–	**2.247381**	**0.155910**	**1.875606**	**2.919213**
RIME^85^	2023	6.088093	2.370950	2.327531	10.603060
SABO^55^	2023	9.047732	1.317916	4.277977	11.347119
CDO^86^	2023	9.008964	1.115954	6.738168	11.681142
EVO^87^	2023	9.182397	3.055184	3.050956	19.666239
GO^88^	2023	2.623891	1.020858	1.909703	7.385158
GAO^89^	2023	8.881956	0.627587	6.261991	10.633934
DSO^90^	2023	9.092956	1.619127	6.271599	12.685378
CPO^91^	2024	6.249072	1.028020	4.147711	8.526846
PO^92^	2024	6.225141	2.613692	2.262198	10.451013
NRBO^93^	2024	8.145156	2.018604	3.830352	11.633929
FTTA^94^	2024	6.339779	2.404930	2.371015	10.749497

As can be seen in Table 41, the SPO algorithm outperforms the other algorithms in four dimensions: mean, standard deviation, optimal result, and worst result. The optimization results of the SPO algorithm are much better than the other algorithms in terms of mean value, and only the optimization results of the GO algorithm are closer to the results of the SPO algorithm. From the standard deviation point of view, the SPO algorithm has strong robustness. From the perspective of optimal and worst results, the worst results of the SPO algorithm are even better than the optimal results of some algorithms. The results above prove the SPO algorithm has strong search ability and robustness.

**Figure 25:**
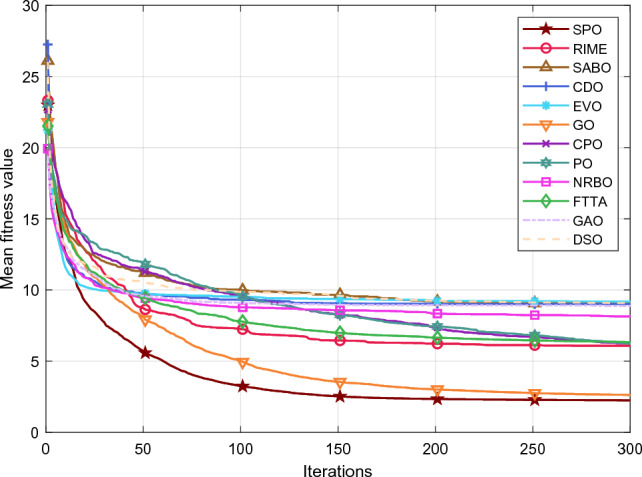
Mean convergence curves of each algorithm for the spacecraft trajectory optimization problem.

Figure 25 demonstrates the variation of the average fitness of all the functions. As can be seen from the figure, the SPO algorithm has the fastest rate of descent and convergence compared to the other algorithms. To analyze the convergence in-depth, we show the results of every 50 iterations in Table 42. The table shows that compared to different algorithms, the SPO algorithm can converge to better results faster and optimize the results continuously. All these show that the SPO algorithm has strong search capability.

**Table 42:** Results of the comparative algorithms for solving the spacecraft trajectory optimization problem.

Methods	50 iterations	100 iterations	150 iterations	200 iterations	250 iterations	300 iterations
SPO	**5.694379**	**3.255334**	**2.527333**	**2.343147**	**2.282162**	**2.247381**
RIME	8.730952	7.260992	6.460082	6.228749	6.125330	6.088093
SABO	11.244063	10.035926	9.652703	9.182677	9.068723	9.047732
CDO	9.720643	9.326924	9.063085	9.034216	9.008964	9.008964
EVO	9.740769	9.540687	9.369254	9.236747	9.211324	9.182397
GO	8.132490	5.027592	3.551335	3.017829	2.756796	2.623891
GAO	9.588816	9.093274	8.961006	8.934323	8.912699	8.881956
DSO	10.491133	9.856996	9.603108	9.257017	9.121431	9.092956
CPO	11.361176	9.602220	8.245777	7.387805	6.718003	6.249072
PO	11.829681	9.571721	8.275387	7.435847	6.821424	6.225141
NRBO	9.431107	8.790877	8.583863	8.343244	8.237440	8.145156
FTTA	9.478770	7.803040	6.997689	6.651905	6.462972	6.339779

## Conclusion and outlook

In this paper, the powerful mathematical tool Fourier series is successfully applied to the search process of metaheuristic algorithms. The search for the optimal position on a specific projective plane in space is realized by using the fundamental wave of the Fourier series, and the above process is completed quickly using three symmetry points. This search process is called the symmetric projection search method. Furthermore, a symmetric projection optimizer (SPO) is constructed. In SPO, both global and local search modes are accomplished using only one set of update procedures, which is achieved by controlling the distance between three points.

The SPO algorithm has been tested on seven types of CEC, including three dimensions of CEC2017, CEC2019, CEC2020, and two dimensions of CEC2022. It also has been tested on four real-world engineering projects. The powerful MBAs and OBAs, which have been proposed in recent years, were chosen for comparison experiments, respectively. The experiments show that the SPO algorithm ranks first in all tests compared to all other algorithms and performs even better in high-dimensional problems. Meanwhile, Wilcoxon's rank sum test results prove that the SPO algorithm has a significant advantage over all algorithms in 94.6% of all tests.

From the experimental results, it is necessary to explain the main findings of this article clearly:

1. Successful application of the fundamental wave of the Fourier series to the search process provides a new search mechanism for metaheuristic algorithms.
2. Using symmetric points in the same projection plane simplifies the computational process and improves computational efficiency.
3. The search process is simplified using the same formula to complete global exploration and local exploitation processes.
4. The SPO algorithm has few control parameters.

Although the SPO algorithm shows more excellent results in all aspects, it still has certain defects. The main limitation is in the local search. Although the SP search mechanism can complete the local search and work better, from the point of view of the optimal value of the search, the SPO algorithm still has room for improvement. Therefore, in future work, the primary research focuses on two aspects. One is to enhance the local search capability of the SPO algorithm by introducing other mechanisms, and the other is that the SPO algorithm will be fully applied in more spacecraft trajectory optimization problems.

## Data Availability

The datasets used and/or analysed during the current study available from the corresponding author on reasonable request.

## References

[1] Abualigah L, Diabat A, Thanh C-L, Khatir S (2023). Opposition-based Laplacian distribution with Prairie Dog Optimization method for industrial engineering design problems. Comput. Methods Appl. Mech. Eng..

[2] Piskin A, Baklacioglu T, Turan O (2022). Optimization and off-design calculations of a turbojet engine using the hybrid ant colony—particle swarm optimization method. Aircr. Eng. Aerosp. Technol..

[3] Xu Y, Tang H, Chen M (2022). Design method of optimal control schedule for the adaptive cycle engine steady-state performance. Chin. J. Aeronaut..

[4] Qiu S, Li Z, Wang D, Li Z, Tao Y (2023). Active optimization of chilled water pump running number: Engineering practice validation. Sustainability.

[5] Sun F-F, Yu R-J, Jia R-Z (2023). Practical optimal design method for multi-outrigger building structures under inter-story drift constraints. Adv. Struct. Eng..

[6] Delavar MR, Ramezanzadeh A, Gholami R, Sanei M (2023). Optimization of drilling parameters using combined multi-objective method and presenting a practical factor. Comput. Geosci..

[7] Razali MR, Mohd Faudzi AA, Shamsudin AU, Mohamaddan S (2023). A hybrid controller method with genetic algorithm optimization to measure position and angular for mobile robot motion control. Front. Robot. AI.

[8] Zhu Y, Qiu B, Li W (2023). Trajectory optimization method based on ellipse model for dynamic motion control of piezoelectric transducer in an optical resonance cavity. Precis. Eng..

[9] Shi B, Peng H, Wang X, Zhong W (2022). A symplectic direct method for motion-driven optimal control of mechanical systems. Commun. Nonlinear Sci. Numer. Simul..

[10] Hu G, Zhong J, Wei G, Chang C-T (2023). DTCSMO: An efficient hybrid starling murmuration optimizer for engineering applications. Comput. Methods Appl. Mech. Eng..

[11] Dan F, Bo L, Jian G (2023). An on-board task scheduling method based on evolutionary optimization algorithm. J. Circuits Syst. Comput..

[12] Chen Z, Wei P, Li Y (2022). Combining neural network-based method with heuristic policy for optimal task scheduling in hierarchical edge cloud. Digit. Commun. Netw..

[13] Faramarzi A, Heidarinejad M, Stephens B, Mirjalili S (2020). Equilibrium optimizer: A novel optimization algorithm. Knowl.-Based Syst..

[14] Smith KD, Bullo F (2023). Convex optimization of the basic reproduction number. IEEE Trans. Autom. Control.

[15] Kováčová G, Rudloff B (2022). Convex projection and convex multi-objective optimization. J. Glob. Optim..

[16] Mu R, Deng Y, Wu P (2023). Adaptive convex optimization guidance for lunar landing. Aerospace.

[17] Guan Z, Ren C, Niu J, Wang P, Shang Y (2023). Great Wall Construction Algorithm: A novel meta-heuristic algorithm for engineer problems. Expert Syst. Appl..

[18] Liu C (2023). An improved heuristic mechanism ant colony optimization algorithm for solving path planning. Knowl.-Based Syst..

[19] Kaveh M, Mesgari MS, Saeidian B (2023). Orchard Algorithm (OA): A new meta-heuristic algorithm for solving discrete and continuous optimization problems. Math. Comput. Simul..

[20] Katoch S, Chauhan SS, Kumar V (2021). A review on genetic algorithm: Past, present, and future. Multimed. Tools Appl..

[21] Price KV, Storn RM, Lampinen J (2005). Differential evolution—A practical approach to global optimization. Nat. Comput..

[22] Ma X (2019). A survey on cooperative co-evolutionary algorithms. IEEE Trans. Evol. Comput..

[23] Sulaiman MH, Mustaffa Z, Saari MM, Daniyal H, Mirjalili S (2022). Evolutionary mating algorithm. Neural Comput. Appl..

[24] Alagoz BB (2022). An Evolutionary field theorem: Evolutionary field optimization in training of power-weighted multiplicative neurons for nitrogen oxides-sensitive electronic nose applications. Sensors.

[25] Zamani H, Nadimi-Shahraki MH, Gandomi AH (2021). QANA: Quantum-based avian navigation optimizer algorithm. Eng. Appl. Artif. Intell..

[26] Fang C (2023). High-efficient memristive genetic algorithm for feature selection. IEEE Trans. Electron Devices.

[27] Chen B (2023). Prediction of an epidemic spread based on the adaptive genetic algorithm. Front. Phys..

[28] Zheng J, Zhong J, Chen M, He K (2023). A reinforced hybrid genetic algorithm for the traveling salesman problem. Comput. Oper. Res..

[29] Poli R, Kennedy J, Blackwell T (2007). Particle swarm optimization. Swarm Intell..

[30] Dorigo M, Birattari M, Stutzle T (2006). Ant colony optimization. IEEE Comput. Intell. Mag..

[31] Zamani H, Nadimi-Shahraki MH, Gandomi AH (2022). Starling murmuration optimizer: A novel bio-inspired algorithm for global and engineering optimization. Comput. Methods Appl. Mech. Eng..

[32] Zamani H (2024). An evolutionary crow search algorithm equipped with interactive memory mechanism to optimize artificial neural network for disease diagnosis. Biomed. Signal Process. Control.

[33] Zamani H, Nadimi-Shahraki MH, Mirjalili S (2024). A critical review of moth-flame optimization algorithm and its variants: Structural reviewing, performance evaluation, and statistical analysis. Arch. Comput. Methods Eng..

[34] Nadimi-Shahraki MH, Zamani H, Fatahi A, Mirjalili S (2023). MFO-SFR: An enhanced moth-flame optimization algorithm using an effective stagnation finding and replacing strategy. Mathematics.

[35] Nadimi-Shahraki MH, Zamani H, Asghari Varzaneh Z (2023). A systematic review of the whale optimization algorithm: Theoretical foundation, improvements, and hybridizations. Arch. Comput. Methods Eng..

[36] Zhao S, Zhang T, Ma S, Chen M (2022). Dandelion optimizer: A nature-inspired metaheuristic algorithm for engineering applications. Eng. Appl. Artif. Intell..

[37] Orujpour M, Feizi-Derakhshi M-R, Rahkar-Farshi T (2020). Multi-modal forest optimization algorithm. Neural Comput. Appl..

[38] Wang P, Huang J, He W, Zhang J, Guo F (2022). Maximum likelihood DOA estimation based on improved invasive weed optimization algorithm and application of MEMS vector hydrophone array. AIMS Math..

[39] Ghasemian H, Ghasemian F, Vahdat-Nejad H (2020). Human urbanization algorithm: A novel metaheuristic approach. Math. Comput. Simul..

[40] Lian J, Hui G (2024). Human evolutionary optimization algorithm. Expert Syst. Appl..

[41] Ahmadi S-A (2017). Human behavior-based optimization: A novel metaheuristic approach to solve complex optimization problems. Neural Comput. Appl..

[42] Fattahi E, Bidar M, Kanan HR (2018). Focus group: An optimization algorithm inspired by human behavior. Int. J. Comput. Intell. Appl..

[43] Zhang P (2023). A novel human learning optimization algorithm with Bayesian inference learning. Knowl.-Based Syst..

[44] Cheng S, Qin Q, Chen J, Shi Y (2016). Brain storm optimization algorithm: A review. Artif. Intell. Rev..

[45] Kirkpatrick S (1984). Optimization by simulated annealing: Quantitative studies. J. Stat. Phys..

[46] Gong L, Hou G, Huang C (2023). A two-stage MPPT controller for PV system based on the improved artificial bee colony and simultaneous heat transfer search algorithm. ISA Trans..

[47] Goodarzimehr V, Shojaee S, Hamzehei-Javaran S, Talatahari S (2022). Special relativity search: A novel metaheuristic method based on special relativity physics. Knowl.-Based Syst..

[48] Abdel-Basset M, El-Shahat D, Jameel M, Abouhawwash M (2023). Young's double-slit experiment optimizer: A novel metaheuristic optimization algorithm for global and constraint optimization problems. Comput. Methods Appl. Mech. Eng..

[49] Hashim FA, Mostafa RR, Hussien AG, Mirjalili S, Sallam KM (2023). Fick's Law Algorithm: A physical law-based algorithm for numerical optimization. Knowl.-Based Syst..

[50] Ghasemi M (2022). Application of Coulomb’s and Franklin’s laws algorithm to solve large-scale optimal reactive power dispatch problems. Soft Comput..

[51] Daoud MS (2023). Gradient-based optimizer (GBO): A review, theory, variants, and applications. Arch. Comput. Methods Eng. State Art Rev..

[52] Zhang Y, Jin Z, Mirjalili S (2020). Generalized normal distribution optimization and its applications in parameter extraction of photovoltaic models. Energy Convers. Manag..

[53] Rezaei F, Safavi HR, Elaziz MA, Mirjalili S (2023). GMO: Geometric mean optimizer for solving engineering problems. Soft Comput. Fusion Found. Methodol. Appl..

[54] Abualigah L, Diabat A, Mirjalili S, Abd Elaziz M, Gandomi AH (2021). The arithmetic optimization algorithm. Comput. Methods Appl. Mech. Eng..

[55] Pavel Trojovský MD (2023). Subtraction-average-based optimizer: A new swarm-inspired metaheuristic algorithm for solving optimization problems. Biomimetics.

[56] Zhao W (2023). Quadratic Interpolation Optimization (QIO): A new optimization algorithm based on generalized quadratic interpolation and its applications to real-world engineering problems. Comput. Methods Appl. Mech. Eng..

[57] Zhao S (2023). Triangulation Topology Aggregation Optimizer: A novel mathematics-based meta-heuristic algorithm for engineering applications. Expert Syst. Appl..

[58] Wu, G., Mallipeddi, R. & Suganthan, P. N. Problem definitions and evaluation criteria for the CEC 2017 competition on constrained real-parameter optimization. *National University of Defense Technology, Changsha, Hunan, PR China and Kyungpook National University, Daegu, South Korea and Nanyang Technological University, Singapore, Technical Report* (2017).

[59] Liang J-J, Qu B, Gong D, Yue C (2019). Problem Definitions and Evaluation Criteria for the CEC 2019 Special Session on Multimodal Multiobjective Optimization.

[60] Parmaksiz H, Yuzgec U, Dokur E, Erdogan N (2023). Mutation based improved dragonfly optimization algorithm for a neuro-fuzzy system in short term wind speed forecasting. Knowl.-Based Syst..

[61] Wu L (2023). Smooth Exploration System: A novel ease-of-use and specialized module for improving exploration of whale optimization algorithm. Knowl.-Based Syst..

[62] Webb M, Coppé V, Huybrechs D (2019). Pointwise and uniform convergence of Fourier extensions. Construct. Approx..

[63] Yang Y, Zhou C, Zhang H, Peng Y, Sun H (2023). Denoising CSEM data using least-squares method based on mixed basis of Fourier series and Legendre polynomials. IEEE Trans. Geosci. Remote Sens..

[64] Rodrigues LR (2023). A chaotic grey wolf optimizer for constrained optimization problems. Expert Syst..

[65] Du J (2023). An adaptive human learning optimization with enhanced exploration–exploitation balance. Ann. Math. Artif. Intell..

[66] Mirjalili S (2016). SCA: A sine cosine algorithm for solving optimization problems. Knowl.-Based Syst..

[67] Ahmadianfar I, Heidari AA, Gandomi AH, Chu X, Chen H (2021). RUN beyond the metaphor: An efficient optimization algorithm based on Runge Kutta method. Expert Syst. Appl..

[68] Ahmadianfar I, Heidari AA, Noshadian S, Chen H, Gandomi AH (2022). INFO: An efficient optimization algorithm based on weighted mean of vectors. Expert Syst. Appl..

[69] Bai J (2023). A Sinh Cosh optimizer. Knowl.-Based Syst..

[70] Abdel-Basset M, El-Shahat D, Jameel M, Abouhawwash M (2023). Exponential distribution optimizer (EDO): a novel math-inspired algorithm for global optimization and engineering problems. Artif. Intell. Rev..

[71] Zhao S, Zhang T, Cai L, Yang R (2023). Triangulation Topology Aggregation Optimizer: A novel mathematics-based meta-heuristic algorithm for engineering applications. Expert Syst. Appl..

[72] Abdollahzadeh B, SoleimanianGharehchopogh F, Mirjalili S (2021). Artificial gorilla troops optimizer: A new nature-inspired metaheuristic algorithm for global optimization problems. Int. J. Intell. Syst..

[73] Hashim FA, Hussien AG (2022). Snake optimizer: A novel meta-heuristic optimization algorithm. Knowl.-Based Syst..

[74] Abdel-Basset M, Mohamed R, Azeem SAA, Jameel M, Abouhawwash M (2023). Kepler optimization algorithm: A new metaheuristic algorithm inspired by Kepler’s laws of planetary motion. Knowl.-Based Syst..

[75] Hu G, Guo Y, Wei G, Abualigah L (2023). Genghis Khan shark optimizer: A novel nature-inspired algorithm for engineering optimization. Adv. Eng. Inform..

[76] Moriyama, T. & Maesono, Y. Smoothed nonparametric two-sample tests. *Statistics* (2017).

[77] Tzanetos A, Blondin M (2023). A qualitative systematic review of metaheuristics applied to tension/compression spring design problem: Current situation, recommendations, and research direction. Eng. Appl. Artif. Intell..

[78] Heidari AA (2019). Harris hawks optimization: Algorithm and applications. Future Gener. Comput. Syst..

[79] Xue J, Shen B (2020). A novel swarm intelligence optimization approach: Sparrow search algorithm. Syst. Sci. Control Eng..

[80] Mirjalili S, Mirjalili SM, Lewis A (2014). Grey Wolf optimizer. Adv. Eng. Softw..

[81] Pathak VK, Srivastava AK (2022). A novel upgraded bat algorithm based on cuckoo search and Sugeno inertia weight for large scale and constrained engineering design optimization problems. Eng. Comput..

[82] Yıldız BS (2023). A novel hybrid arithmetic optimization algorithm for solving constrained optimization problems. Knowl.-Based Syst..

[83] Savsani P, Savsani V (2016). Passing vehicle search (PVS): A novel metaheuristic algorithm. Appl. Math. Model..

[84] Su H, Dong Z, Liu L, Xia L (2022). Numerical solution for the single-impulse flyby co-orbital spacecraft problem. Aerospace.

[85] Su HRIME (2023). A physics-based optimization. Neurocomputing.

[86] Shehadeh HA (2023). Chernobyl disaster optimizer (CDO): A novel meta-heuristic method for global optimization. Neural Comput. Appl..

[87] Azizi M, Aickelin U, Khorshidi HA, Shishehgarkhaneh MB (2023). Energy valley optimizer: A novel metaheuristic algorithm for global and engineering optimization. Sci. Rep..

[88] Zhang Q, Gao H, Zhan Z-H, Li J, Zhang H (2023). Growth optimizer: A powerful metaheuristic algorithm for solving continuous and discrete global optimization problems. Knowl.-Based Syst..

[89] Alsayyed O, Hamadneh T, Al-Tarawneh H, Alqudah M, Gochhait S, Leonova I, Malik OP, Dehghani M (2023). Giant Armadillo optimization: A new bio-inspired metaheuristic algorithm for solving optimization problems. Biomimetics.

[90] Oladejo SO, Ekwe SO, Akinyemi LA, Mirjalili SA (2023). The deep sleep optimizer: A human-based metaheuristic approach. IEEE Access.

[91] Abdel-Basset M (2024). Crested Porcupine Optimizer: A new nature-inspired metaheuristic. Knowl.-Based Syst..

[92] Abdollahzadeh B, Khodadadi N, Barshandeh S (2024). Puma optimizer (PO): A novel metaheuristic optimization algorithm and its application in machine learning. Cluster Comput..

[93] Sowmya R (2024). Newton-Raphson-based optimizer: A new population-based metaheuristic algorithm for continuous optimization problems. Eng. Appl. Artif. Intell..

[94] Tian Z (2024). Football team training algorithm: A novel sport-inspired meta-heuristic optimization algorithm for global optimization. Expert Syst. Appl..

